# Systematics of giant neotropical fireflies and their kin (Lampyridae: Lampyrinae)

**DOI:** 10.1371/journal.pone.0354465

**Published:** 2026-08-03

**Authors:** William Lima, Viviane C. S. Nunes, Leandro Zeballos, Angie Gisseth Ladino Peñuela, Claudio Ruy Vasconcelos da Fonseca, Luiz Felipe Lima da Silveira

**Affiliations:** 1 Instituto Nacional de Pesquisas da Amazônia, Coordenação de Biodiversidade, Laboratório de Sistemática e Ecologia de Coleoptera, Petrópolis, Manaus, Brazil; 2 Institute of Evolution and Ecology, Evolutionary Biology of Invertebrates, University of Tübingen, Tübingen, Germany; 3 Grupo de Investigación en Sistemática Molecular, Maestría en Ciencias–Entomología, Universidad Nacional de Colombia, Sede Medellín, Antioquia, Colombia; 4 Biology Department, Western Carolina University, Cullowhee, North Carolina, United States of America; Universidade Federal de Minas Gerais, BRAZIL

## Abstract

The Lampyrinae tribe Cratomorphini includes some of the largest and most widespread New World fireflies. Their overlapping genus-level diagnoses are based on a few traits related to sexual signaling known to be fast-evolving and labile within-genus in other Lampyrinae taxa. To clarify the taxonomy and investigate the morphological evolution in Cratomorphini, Lamprocerini, and closely related taxa, we inferred the phylogenetic relationships of 50 species using 97 adult morphological characters contrasting the results of Maximum Parsimony Analysis and Bayesian Inference. Using a statistical framework, we demonstrate that terminalia and genitalic traits are less homoplastic than signaling or other somatic traits. Cratomorphini was recovered as polyphyletic, with four distinct clades, as follows: (i) *Cratomorphus* (partim: the type species, *C. splendidus*, and associated species) + *Erythrolychnia bipartita*; (ii) *Cratomorphus* (partim; transferred here to ***Nyctocera*** **gen. nov.** [Lamprocerini]); (iii) *Cratomorphus* (partim; transferred here to ***Bituca*** **gen. nov.** [Lampyrinae *incertae sedis*]) and (iv) (*Micronaspis* ((*Aspisomoides, Pyractomena*) *Aspisoma*)). Based on these results, we redefine Cratomorphini and Lamprocerini and present updated diagnoses for both tribes. We propose a new tribe, **Aspisomini trib. nov.** and establish two new genera: *Nyctocera* **gen. nov.** (within Lamprocerini **sensu nov.**) and *Bituca* **gen. nov.** (*incertae sedis*). Four species formerly assigned to *Cratomorphus* **sensu nov.** are transferred *–* three to ***Nyctocera*** **gen. nov.**, and one to ***Bituca*** **gen. nov.** – and all are redescribed based on type and additional material. We illustrate all diagnostic features, and provide updated keys to the genera of Cratomorphini **sensu nov.**, Aspisomini **trib. nov.**, and Lamprocerini **sensu nov.** Additionally, we identify and discuss synapomorphies for clades previously recovered only in DNA-based phylogenies. Our work stresses the need for thorough investigations of morphological data, especially of the terminalia and genitalia, to elucidate the phylogeny of fireflies.

## 1. Introduction

Lampyrinae Rafinesque, 1815 [1,2] is the largest subfamily of Lampyridae, and includes five tribes: Cratomorphini Green, 1948; Lamprocerini Olivier, 1907; Lampyrini Rafinesque, 1815; Lucidotini Lacordaire, 1857, and Pleotomini Summers, 1875. Among these, Cratomorphini stands out for including the largest (reaching up to 35 mm in total body length in the adult stage) and most widespread New World fireflies [3,4]. Distributed across the Nearctic and Neotropical regions, most species are associated with swamps and ponds, though some inhabit forests, brackish waters, or even rocky shores [3,5,6]. This ecological diversity is mirrored in the larval stage, which includes the only intertidal larvae in the New World and the sole larva with pronotal lanterns [see 7, 6 respectively; reviewed in 8].

Cratomorphini *sensu* Martin *et al*. [2] includes 130 species in seven genera [9]: *Aspisoma* Laporte, 1833 (58 spp.); *Aspisomoides* Zaragoza-Caballero, 1995 (2 spp.); *Cassidomorphus* Motschulsky, 1853 (1 sp.); *Cratomorphus* Motschulsky, 1853 (41 spp.); *Micronaspis* Green, 1948 (2 spp.); *Paracratomorphus* Zaragoza-Caballero, 2013 (1 sp.); and *Pyractomena* Melsheimer, 1845 (25 spp.) [see 10]. Adults are often large to very large (>25 mm), sometimes called giant fireflies [3], and can be diagnosed by small, glabrous mandibles with a distinct curvature, labrum not covering mandibles, compact and stout tarsi, terminal tarsomere extending slightly beyond the lobes of IV, dorsal abdominal spiracles, and winged and sexually monomorphic forms [11].

The definition and monophyly of Cratomorphini are contentious, as several taxa currently placed in Cratomorphini do not fit this diagnosis and morphological studies often conflict with molecular data. For example, *Cratomorphus besckei* Olivier, 1895 [12] and *C. picipennis* Gorham, 1881 (W.L., pers. obs.) have ventral abdominal spiracles, thus fitting the diagnoses of some Lamprocerini taxa (e.g., *Lucernuta* Laporte, 1833 – see [12]). Furthermore, tribal assignments have been unstable.

Kazantsev & Perez-Gelabert [13] considered some characteristics of the mandible (*viz*. glabrous and narrow apex) as possible synapomorphies, which subsidized the transfer of *Callopisma* Motschulsky, 1853 and *Erythrolychnia* Motschulsky, 1853 from Photinini LeConte, 1881 (= Lucidotini Lacordaire, 1857) to Cratomorphini. However, these transfers remain to be tested by phylogenetic analyses. Martin *et al.* [2] later transferred *Callopisma* and *Erythrolychnia* back to Photinini, although without justification. Some traits of *Erythrolychnia* (as seen in [13] and [14]) are consistent with their placement in Cratomorphini instead of Lucidotini, such as reduced mandibles, wide phallus, and membranous apices of the parameres (since all known Lucidotini have regular-sized mandibles and phallus narrower than or as narrow as parameres; see [14,15]).

Despite taxonomic efforts [e.g., [3,4,6,12,16–23]], a comprehensive review for Cratomorphini based on phylogenetic analyses is still lacking. Previous studies either focused on higher-level lampyrid relationships [e.g., [2,24,25]] or included limited taxon sampling to test the monophyly of this tribe, with only a few genera included [e.g., 2 [2 genera]; [25–27] [3 genera]; [28] [3 genera];). Furthermore, only 8 of the 130 Cratomorphini species have been included in phylogenies [2], largely neglecting its remarkable diversity. In addition to this limited taxonomic sampling, the lack of key taxonomic traits – such as terminalia and genitalic structures, which are useful for discriminating taxa across Lampyridae [e.g., [29,30]] – in previous comparative studies further complicates the systematics of the group [but see 7]. In fact, *Cratomorphus* Motschulsky, 1853 alone features an outstanding morphological diversity across its 41 species, which prompted Olivier [31] to classify them into four groups based on sternite VIII morphology: (I) bilobate with a medial filiform projection; (II) sharply bilobate; (III) gently bilobate/truncate; and (IV) medially projected. However, the phylogenetic validity of these groups remains untested.

The monophyly of Cratomorphini has always been challenged when tested, although taxonomic acts were not taken. Morphology-based phylogenies suggested its paraphyly or polyphyly, while molecular data consistently recover the tribe as polyphyletic. For example, Stanger–Hall *et al.* [28] found *Aspisoma*, *Micronaspis*, and *Pyractomena* scattered across Lampyrinae, and Martin *et al.* [25] recovered Cratomorphini as paraphyletic when only molecular data were analyzed, and as polyphyletic when both datasets were combined. A recent DNA-based phylogeny split the tribe into two separate clades: *Cratomorphus*, and (*Pyractomena*+*Aspisoma*) [2]. However, these results are yet to be contrasted with comprehensive and thorough morphology-based phylogenetic analysis.

Here, we tested the monophyly of Cratomorphini based on an extensive dataset of 97 morphological characters of 50 species, including 35 species representing 5 of 7 Cratomorphini genera, with a denser sampling of the two most speciose genera (*Cratomorphus* and *Aspisoma*), and representatives of all lampyrine tribes. Our phylogenetic analyses included previously neglected terminalia and genitalic structures, and contrasted the results of Bayesian Inference and Maximum Parsimony to establish a more robust systematic framework. Furthermore, we investigated character-state transformations – specifically distinguishing among independent gains, reversals, and losses – to assess character lability and stability across different taxonomic levels. Our results called for the taxonomic revisions proposed herein.

## 2. Materials and methods

### 2.1 *Material examined, terminology and preparation*

We studied specimens, including type material, from the following institutions: INPA – Coleção Sistemática de Entomologia do Instituto Nacional de Pesquisas da Amazônicas, Manaus, Amazonas, Brazil (Dr. Marcio Luiz de Oliveira); CZPB – Coleção Zoológica Professor Paulo Bührnheim, Universidade Federal do Amazonas, Manaus, Amazonas (Dr. Sérgio Luis Gianizella); MPEG – Museu Paraense Emílio Goeldi, Belém, Pará, Brazil (Dr. Orlando Tobias); DZRJ – Coleção Entomológica Professor José Alfredo Pinheiro Dutra, Universidade Federal do Rio de Janeiro, Rio de Janeiro, Brazil (Dr. José Ricardo Miras Mermudes); MZSP – Museu de Zoologia, Universidade de São Paulo, São Paulo, Brazil (Dr. Sônia Aparecida Casari); DZUP – Coleção Entomológica Pe. Jesus Santiago Moure, Universidade Federal do Paraná, Curitiba, Brazil (Dr. Lúcia Massutti Almeida); UFMT – Coleção zoológica da Universidade Federal de Mato Grosso, Brazil (Dr. Fernando Zagury Vaz–de–Mello); CEIOC – Coleção de Entomologia do Instituto Oswaldo Cruz, Rio de Janeiro, Brazil (Dr. Márcio Felix); MNHN – Muséum National d’Histoire Naturelle, Paris, France (Dr. Antoine Mantilleri); BMNH – The Natural History Museum, London, United Kingdom (Dr. Michael Geiser); USNM – National Museum of Natural History, Washington, D.C., United States of America (Dr. Marc Branham); MPUJ – Museo de Historia Natural de la Pontificia Universidad Javeriana, Bogotá, Colombia (Dr. Lucas Barrientos); MNH-ENT – Colección Entomologica del Museo de Historia Natural Luis Gonzalo Andrade de la Universidad Pedagogica y Tecnológica de Colombia (Dr. Fredy Molano Rendón); MTKD – Museum für Tierkunde Dresden, Dresden, Germany (Dr. Olaf Jäger); CNIN – National Insect Collection, Institute of Biology, Universidad Nacional Autónoma de México, México (Dr. Santiago Zaragoza-Caballero); WCCA – Western Carolina University Collection of Arthropods (Dr. Luiz F. L. Silveira); NCSU – North Carolina State University Insect Collection (Brian Wiegmann); NMB – Naturhistorisches Museum Basel, Switzerland (Dr. Matthias Borer).

For the anatomical comparisons, we softened and clarified specimens in a 10% potassium hydroxide (KOH) solution for 24h at room temperature, then specimens were dissected and examined under a Leica EZ4HD or Leica M205C stereoscopic microscopes. We obtained images and measurements in a Leica EZ4HD with LAS 4.2 software, or a Leica M205C coupled with a DF5400 camera, and later processed in LAS X software. We stacked and processed a series of images with the software Helicon Focus ® version 5.3, and later edited them in Adobe Photoshop® CC 2020, Adobe Illustrator® CC 2021, and Inkscape version 1.2. Distribution maps of the species were made from shapefiles available in Morrone *et al*. [32] using QGIS 3.10.13 [33] and edited using Illustrator® CC 2021. Drawings were made using Procreate v. 5.1.8, then vectorized and edited using Inkscape version 1.2.

For tribe-level classification, we followed Martin *et al.* [2], while the definitions of genera were based on McDermott [10] and Silveira *et al.* [12], compiled in [9]. Terminology for ovipositor morphology has often been inconsistent, due to the lack of a standardized homology framework in Lampyridae. In the present study, we adopt the homology scheme outlined by Lawrence *et al.* [34] and further detailed by Genevcius *et al.* [35]. According to this scheme, the ovipositor is composed of a paraproct (*viz.* modified tergite IX), including medially divided laterotergites and supporting baculi, a variably sclerotized proctiger (*viz.* tergite X), and paired gonocoxites, each bearing a gonostylus. Although gonocoxites may show a proximal-distal differentiation in some beetle groups [36], this framework provides a consistent basis for describing the rather simple ovipositor structure in Lampyridae. For other morphological characters, terminology and wing venation follow Silveira *et al.* [12] and Lawrence *et al.* [36], respectively.

### 2.2 *Taxon sampling and character coding*

**Fig 1:**
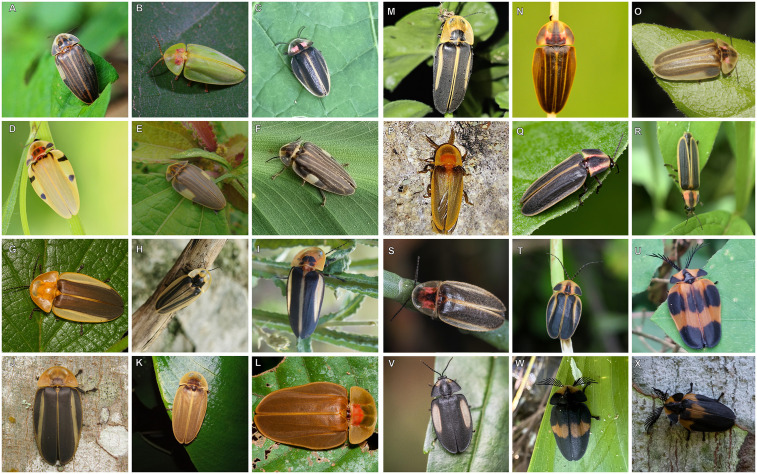
Representatives of the Aspisomini trib. nov., Cratomorphini sensu nov., and Lamprocerini sensu nov. A, *Aspisoma ignitum*; B, *Aspisoma physonotum*; C, *Aspisoma pulchellum*; D, *Aspisoma sticticum*; E, *Aspisoma lineatum*; F, *Aspisoma buyssoni*; G, *Cratomorphus albomarginatus*; H, *Cratomorphus signativentris*; I, *Cratomorphus distinctus*; J, *Cratomorphus splendidus*; K, *Cratomorphus bifenestratus*; L, *Nyctocera discorufa*; M, *Cratomorphus diaphanus*; N, *Aspisomoides bilineatum*; O, *Aspisomoides costatum*; P, *Pleotomus pallens*; Q, *Pyractomena borealis*; R, *Pyractomena lucifera*; S, *Micronaspis floridana*; T, *Tenaspis chamelensis;* U, *Lychnacris flabellata*; V, *Lucernuta savignii*; W, *Lucio pictum*; X, *Lamprocera flavofasciata*.

The terminals included in the phylogenetic analysis are listed in Table 1. To evaluate the monophyly of Cratomorphini within Lampyrinae, 35 species belonging to five of the seven genera of this tribe were selected as ingroup: *Aspisoma*, *Aspisomoides*, *Cratomorphus*, *Micronaspis,* and *Pyractomena* (Fig 1). *Cassidomorphus* and *Paracratomorphus* are only known from their lectotype and holotype respectively, and were not included in our sampling due to unavailability of material for comparisons. We also included genera representing all tribes of Lampyrinae – Lampyrini, Lucidotini, Pleotomini and Lamprocerini, to test the monophyly of Cratomorphini and to provide a comparative context for genera whose tribal placement has been historically contentious. (see Table 1). *Cladodes flabellatus*, the type species of the type-genus of Cladoninae, was chosen to root the trees because it is phylogenetically more distant from Cratomorphini than the other tribes [2]. Primary homology and character states were defined based on similarity, position, and composition [37,38], using direct observations of males and literature data. Our final matrix included 97 morphological characters (see results), coded as binary or multistate, and 50 taxa. The multistate characters were treated as unordered (non-additive). The characters and their states were written following the logical basis of Sereno [39].

**Table 1:** Taxa included in our phylogenetic analyses based on recently collected and musem-based material.

Taxa included in the phylogenetic analysis.				
Sufamily	Tribe	Genus	Species	New placement
Cladodinae		*Cladodes*	*Cladodes flabellata* Solier, 1848	
Lampyrinae	Lampirini	*Lampyris*	*Lampyris noctiluca* (Linnaeus, 1758)	
		*Microphotus*	*Microphotus octhartus* Fall, 1912	
	Photinini	*Photinus*	*Photinus pyralis* (Linnaeus, 1767)	
			*Photinus corruscus* (Linnaeus, 1767)	
		*Erythrolychnia*	*Erythrolychnia bipartita* (E.Olivier, 1912)	
	Pleotomini	*Pleotomus*	*Pleotomus pallens* LeConte, 1866	
		*Pleotomodes*	*Pleotomodes knulli Green,* 1949	
	Lamprocerini	*Lucernuta*	*Lucernuta savignii* (Kirby, 1818)	Lamprocerini **sensu nov.**
		*Tenaspis*	*Tenaspis angularis* (Gorham, 1880)	Lamprocerini **sensu nov.**
			*Tenaspis sinuosa* E.Olivier, 1899	Lamprocerini **sensu nov.**
			*Tenaspis chamelensis* Zaragoza-Caballero, López-Pérez & Rodríguez-Mirón, 2021	Lamprocerini **sensu nov.**
				Lamprocerini **sensu nov.**
		*Lychnacris*	*Lychnacris flabellata* (Fabricius, 1801)	Lamprocerini **sensu nov.**
		*Lucio*	*Lucio pictum* Gorham, 1880	Lamprocerini **sensu nov.**
		*Lamprocera*	*Lamprocera diluta* E.Olivier, 1885	Lamprocerini **sensu nov.**
		*Bituca* **gen. nov.**	*Bituca miltoni* **sp. nov.** Lima & Silveira	
		*Nyctocera* **gen. nov**.	*Nyctocera blattina* **sp. nov.** Silveira & Lima	
	Cratomorphini	*Cratomorphus*	*Cratomorphus fuscipennis Motschulsky, 1854*	*Nyctocera* **gen. nov**
			*Cratomorphus fasciatus* Gorham, 1884	*Nyctocera* **gen. nov**
			*Cratomorphus discorufus* Kirsch, 1865	*Nyctocera* **gen. nov**
			*Cratomorphus beskei* E.Olivier, 1895	*Bituca* **gen. nov.**
			*Cratomorphus picipennis* Gorham, 1881	Cratomorphini **sensu nov.**
			*Cratomorphus diaphanus*	Cratomorphini **sensu nov.**
			*Cratomorphus splendidus* (Drury, 1782)	Cratomorphini **sensu nov.**
			*Cratomorphus albomarginatus* (Laporte, 1840)	Cratomorphini **sensu nov.**
			*Cratomorphus bifenestratus* Gorham, 1880	Cratomorphini **sensu nov.**
			*Cratomorphus dorsalis* (Gyllenhal, 1817)	Cratomorphini **sensu nov.**
			*Cratomorphus signativentris* E.Olivier, 1895	Cratomorphini **sensu nov.**
			*Cratomorphus distinctus* E.Olivier, 1895	Cratomorphini **sensu nov.**
			*Cratomorphus leoneli* Lima, Silveira & Zaragoza-Caballero, 2021	Cratomorphini **sensu nov.**
		
		*Erythrolychnia*	*Erythrolychnia bipartita* (E.Olivier, 1912)	Cratomorphini **sensu nov**.
		*Aspisoma*	*Aspisoma ignitum* (Linnaeus, 1767)	Aspisomini **trib. nov.**
			*Aspisoma lineatum* (Gyllenhal, 1817)	Aspisomini **trib. nov.**
			*Aspisoma buyssoni* (E.Olivier, 1888)	Aspisomini **trib. nov.**
			*Aspisoma laetum* Berg, 1885	Aspisomini **trib. nov.**
			*Aspisoma maculatum* (De Geer, 1774)	Aspisomini **trib. nov.**
			*Aspisoma sticticum* (Gemminger, 1870)	Aspisomini **trib. nov.**
			*Aspisoma physonotum* (Gorham, 1884)	Aspisomini **trib. nov.**
			*Aspisoma gentile* E.Olivier, 1907	Aspisomini **trib. nov.**
			*Aspisoma aelianum* (Gorham, 1884)	Aspisomini **trib. nov.**
			*Aspisoma nigrum* E.Olivier, 1907	Aspisomini **trib. nov.**
			*Aspisoma pulchellum* (Gorham, 1880)	Aspisomini **trib. nov.**
			*Aspisoma lepidum* (Gorham, 1881)	Aspisomini **trib. nov.**
		*Aspisomoides*	*Aspisomoides billineatum* (Gorham, 1880)	Aspisomini **trib. nov.**
			*Aspisomoides costatum* (Gorham, 1880)	Aspisomini **trib. nov.**
		*Pyractomena*	*Pyractomena lucifera* Melsheimer, 1846	Aspisomini **trib. nov.**
			*Pyractomena borealis* (Randall, 1828)	Aspisomini **trib. nov.**
			*Pyractomena ecostata* (LeConte, 1878)	Aspisomini **trib. nov.**
		*Micronaspis*	*Micronaspis floridana* Green, 1948	Aspisomini **trib. nov.**
			*Micronaspis gabrielae* Vaz, Rocha & Silveira, 2021	Aspisomini **trib. nov.**

### 2.3 *Phylogenetic analyses*

**Fig 2:**
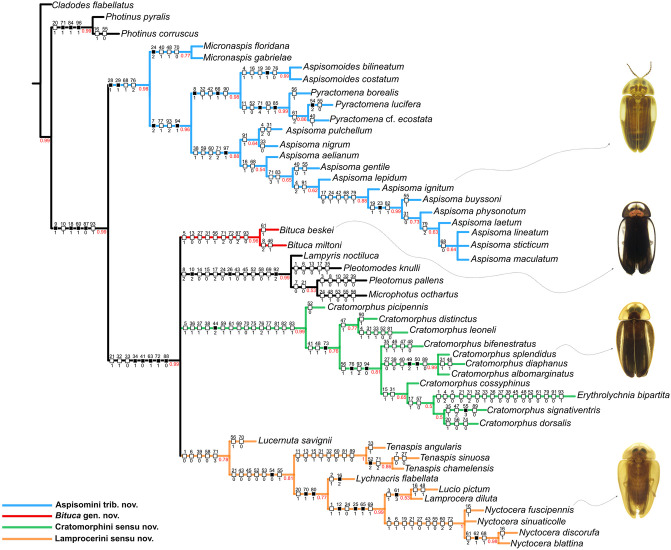
Unambiguous character evolution mapped onto the Bayesian inference consensus tree (see methods for details), with focal taxa color-coded. Non-homoplastic synapomorphies and homoplastic changes are indicated by black and white squares, respectively. Character numbers are shown above branches, while character states are reported below branches. Node support values based on posterior probabilities are given below nodes and highlighted in red.

**Fig 3:**
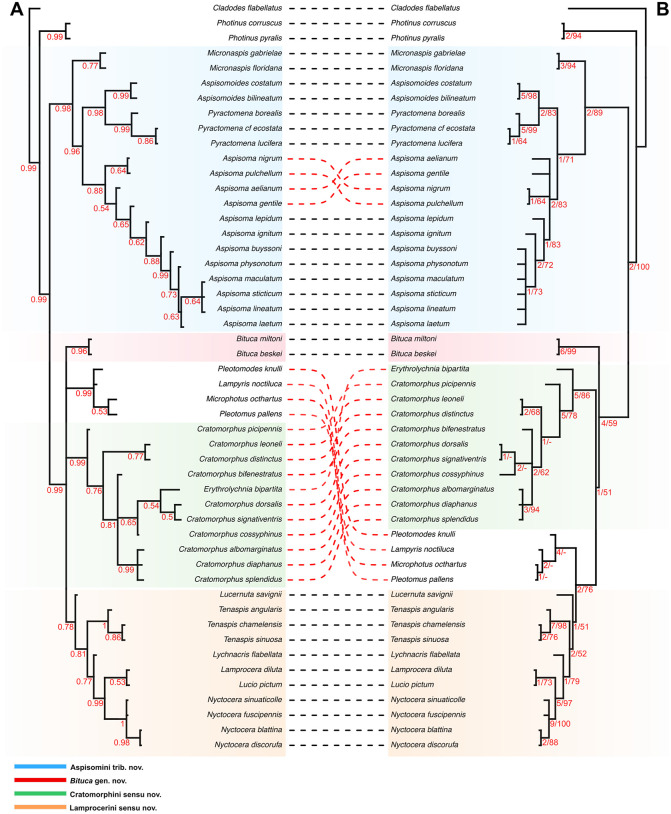
Tanglegram contrasting the results of the Bayesian inference consensus (A) and consensus of 120 most parsimonious trees obtained with Maximum Parsimony with equal weights (B). Posterior probabilities (A) and Bremer/bootstrap (B) supports are shown for each node. Nodes with bootstrap support lower than 50 are scored as “–.”.

Our dataset was assembled in a matrix using MESQUITE version 3.61 [40] (S1). We compared Maximum Parsimony (MP) and Bayesian Inference (BI) to assess topological congruence and robustness across different analytical frameworks. This contrast identifies stable clades recovered regardless of the optimality criterion and highlights nodes sensitive to specific model assumptions (Figs 2–3).

Maximum parsimony analyses were run in TNT [41]. Inapplicable data were scored as ‘–’ and missing data as ‘?’ in the character matrix. The analyses were run using New Technology heuristic searches, with default settings except for Sectorial search (CSS = 100 rounds), Ratchet, Drift (100 cycles), and Tree fusing search (=100 rounds) algorithms, and conducting a driven search with 15 initial addseqs and finding minimum length 50 times [41]. An initial run with equal weights (MPEW) was followed by analyses under implied weights (MPIW) [42] with concavity constants set at uneven intervals (K = 1, 3, 5, 10, and 20; S2) to estimate the effect of homoplastic characters [43]. The k values were not spaced at regular intervals because high k values tend to generate similar results between each other and to those obtained under MPEW analysis [43]. Using regular k values would therefore bias the analysis toward topologies favoured by high k values [44]. Node support was assessed using the Goodman-Bremer decay indices calculated from the unweighted analysis for the clades present in the strict consensus tree using TNT (BD; [45]), which indicate the number of extra steps needed to collapse each clade. Additional branch support was assessed using standard bootstrap resampling (BS), for the MPEW, and symmetric resampling (SR; reported as a range across K-values), for the MPIW, both performed with 1000 replicates. We assessed character stability by the number of extra steps (L) and the retention (RI) index for all 97 traits in R using TreeSearch [46–49], TreeTools [50], and Claddis [51–53] packages. For each character, L and RI was given as mean and standard deviation among the 120 equally most parsimonious trees recovered under MPEW (S3). The mode of L is also given for each character.

Bayesian inference (BI) analyses were implemented in MrBayes 3.2.6 [54], using 10 million generations with trees sampled every 1,000 generations, and the first 25% of trees were discarded as burn-in. We explored two partitioning strategies, since partitioning characters by level of homoplasy has been shown to produce more accurate topologies [55]: the first scheme had characters unpartitioned (S4), and the second one was partitioned by homoplasy (S5), with characters sorted by their average Retention Index (RI = 1 or < 1) across 120 equally most parsimonious trees obtained with the TNT routine described above. We used EW to avoid the arbitrary choice of a concavity constant (*k*) required by traditional Implied Weighting (IW) [55]. The RI = 1 and RI < 1 partitions had 26 and 71 characters, respectively. The Modelfinder [56] implemented in IQTREE2 [57] found a MkV model [58] with equal frequencies, four gamma categories, and controlling for ascertainment bias for the non-partinioned model. For the partitioned scheme, that same model was recovered for the RI < 1 partition, and a simple MkV model controlling for ascertainment bias for the RI = 1 partition. The fit of the two partitioning strategies was evaluated through a Bayes Factor analysis using steppingstone sampling (as proposed by Xie *et al.* [59]) in MrBayes 3.2.6 (S6), comparing likelihoods of each partition obtained over 50 steps sampled across 2,000,000 generations of two independent runs of chains each, and using a burn-in of 25%. Convergence was checked by difference between runs (not consistent, and always >0.002), and by path of the power schedule (log-likelihood trajectory was even and had tight final estimates). The marginal likelihood runs of each partition scheme were averaged and the resulting final marginal likelihood compared using the Bayes Factor formula 2*Δ lnL [60]. In our case, there was very strong evidence (43.68955) in support of the non-partitioned model (S7), which is the only Bayesian Inference reported here. Node support is reported based on the majority consensus tree as posterior probabilities (PP).

Because node support scales up with the number of characters in a matrix [60], they are better interpreted among nodes of a given topology. We used the following standard thresholds to interpret support, which are consistent with interpretations from matrices of similar size: Goodman-Bremer support of 1–2, 3–4, and ≥ 5 indicates weak, moderate and strong support, respectively; while bootstrap, symmetric resampling, or Bayesian PP supports are considered weak, moderate, and strong at ≥ 50–69%, ≥ 70–89%, and ≥90%, respectively [45,54,61]. Convergence was assessed on Tracer v1.6 [62] and resulting trees were visualized in FigTree version 1.4 [63]. Final trees were edited in Adobe Illustrator® CC 2021 and Inkscape version 1.2 for clarity and optimized for publication layout.

Unambiguous character states changes were optimized on the BI topology using Winclada [64] to identify reversals and convergences among the homoplasies.

### 2.4 *Statistical analysis of trait data*

We sought to assess the phylogenetic signal and stability of traits, and specifically compare the stability of the following groups of traits: signaling, terminalic and genitalic, or other somatic (see below). Since a Shapiro-Wilk’s test rejected normality, we compared the among-group difference of mean Retention indices by trait group with a Kruskal-Wallis test, and ran post-hoc pairwise comparisons using a Dunn’s test with Bonferroni correction for multiple group comparisons. To compare the resulting between-group difference to randomly assigned groupings of same sizes, we generated an empirical null distribution of 9999 permutations of group labels, yielding between-group differences. We report the standardized effect size defined as (observed − mean of null differences) / standard deviation of null differences (i.e., the difference between observed and average null proportional to the standard deviation). We also report a permutation p-value (two-tailed) as the proportion of permuted differences with values greater than or equal to the absolute observed difference. These analyses were implemented in R using the dunn.test package [64], and plotted with ggplot2 [65].

## 3. Results

### 3.1 *List of morphological characters*

We delimited 97 characters across the three tagmata: head (15), thorax (23), and abdomen (59). Notably, 32 of these comprise genitalic traits described here for the first time for Cratomorphini taxa. For each character, summary statistics of the number of steps (L) and the retention index (CI) are given in S3 based on 120 equally most parsimonious trees under equal‑weights maximum parsimony (MP-EW).

#### Head.

**Fig 4:**
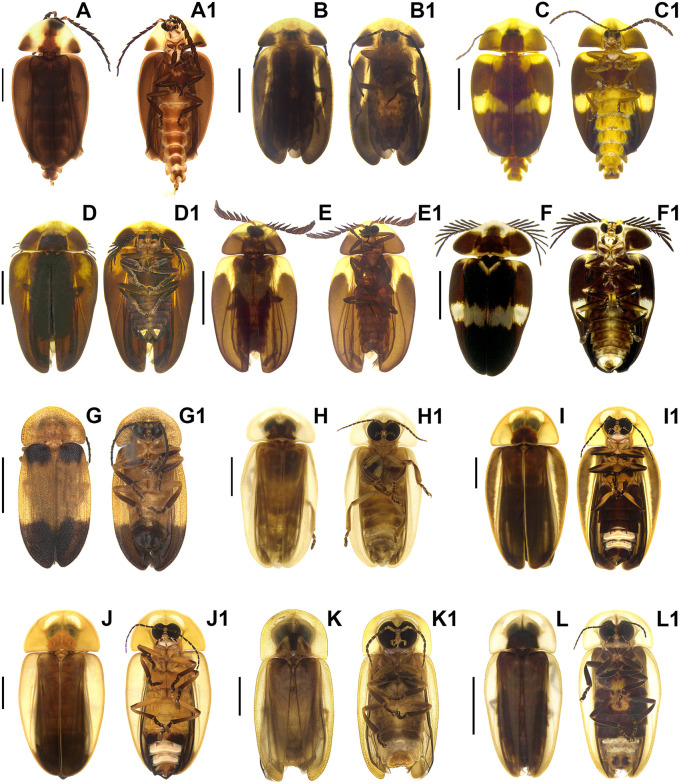
Male habitus, dorsal and ventral view. A, A1: *Tenaspis angularis*. B, B1: *Tenaspis chamelensis*. C, C1: *Tenaspis sinuosa*. D, D1: *Lamprocera diluta*. E, E1: *Lychnacris flabellata*. F, F1: *Lucio pictum*. G, G1: *Erythrolychnia bipartita*. H, H1: *Cratomorphus cossyphinus*. I, I1: *Cratomorphus splendidus*. J, J1: *Cratomorphus albomarginatus*. K, K1: *Cratomorphus dorsalis*. L, L1: *Cratomorphus signativentris*. Scale bar: 3mm.

**Fig 5:**
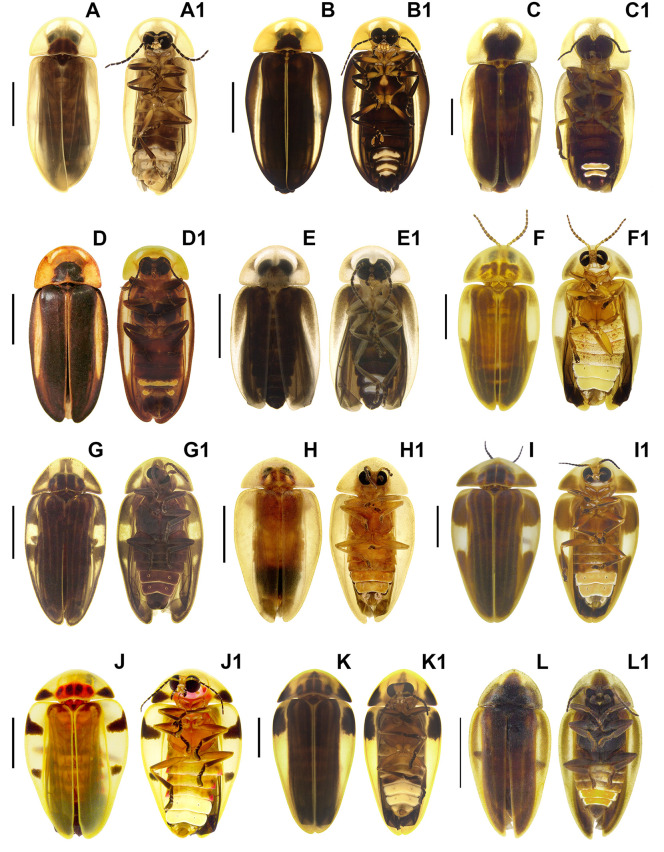
Male habitus, dorsal and ventral view. A, A1: *Cratomorphus bifenestratus*. B, B1: *Cratomorphus distinctus*. C, C1: *Cratomorphus leoneli*. D, D1: *Cratomorphus diaphanus*. E, E1: *Cratomorphus picipennis*. F, F1: *Aspisoma ignitum*. G, G1: *Aspisoma buyssoni*. H, H1: *Aspisoma laetum*. I, I1: *Aspisoma lineatum*. J, J1: *Aspisoma sticticum*. K, K1: *Aspisoma maculatum*. L, L1: *Aspisoma aelianum*. Scale bar: 3mm.

**Fig 6:**
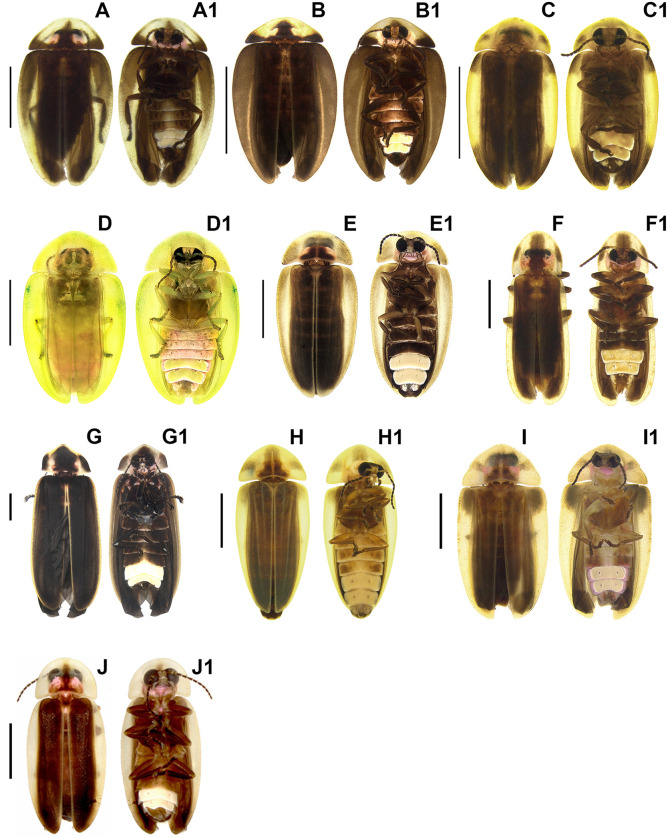
Male habitus, dorsal and ventral view. A, A1: *Aspisoma pulchellum*. B, B1: *Aspisoma nigrum*. C, C1: *Aspisoma lepidum*. D, D1: *Aspisoma physonotum*. E, E1: *Aspisoma gentile*. F, F1: *Pyractomena lucifera*. G, G1: *Pyractomena borealis*. H, H1: *Aspisomoides bilineatum*. I, I1: *Aspisomoides costatum*. J, J1: *Micronaspis floridana*. Scale bar: 3mm.

**Fig 7:**
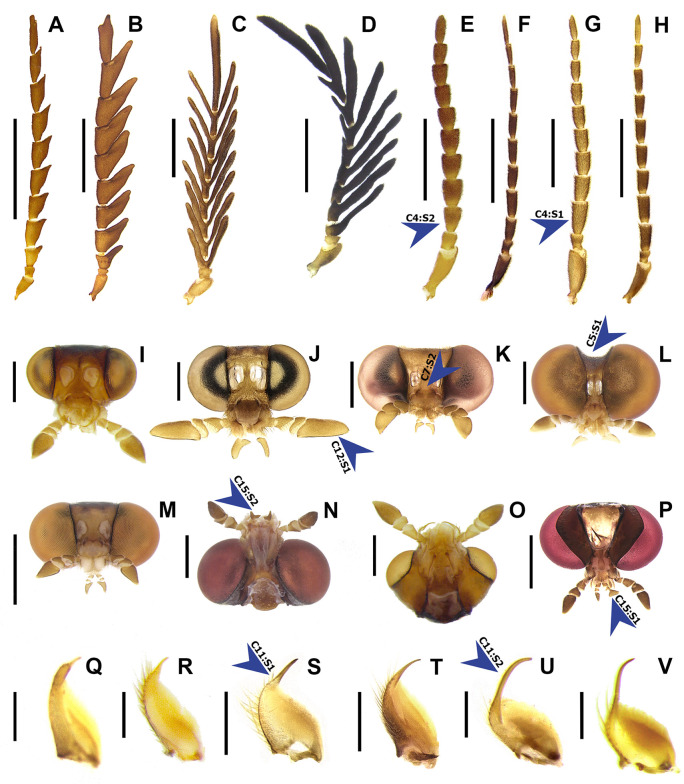
Male. Antenna, lateral view **(A–H)**; head, frontal view **(I–M)**; ventral view **(N–P)**; mandible, dorsal view (Q–V). A, I, O, *Tenaspis sinuosa*. B, Q, *Lychnacris flabellata*. C, *Lamprocera diluta*. D, *Lucio pictum*. E, *Aspisoma maculatum*. F, T, *Cratomorphus distinctus*. G, R, *Cratomorphus leoneli*. H, L, *Cratomorphus bifenestratus*. J, S, *Lamprocera diluta*. K, U, *Aspisoma laetum*. M, *Aspisomoides bilineatum*. N, *Aspisoma lineatum*. P, *Pyractomena* cf. *ecostata.* V, *Aspisoma ignitum*. Scale bar: 1mm **(A–H)**, 0,3mm (Q–V), 2mm **(I–P)**.

1. Head, antenna, antennomeres III–IX, core antennomere (i.e., not including any lamella), shape: (0) serrate (Fig 4A), (1) filiform (Fig 4E1–H1).
2. Head, antenna, antennomeres III–IX, single lamellae: (0) absent, (1) present.
3. Head, antenna, antennomeres III–IX, double lamellae: (0) absent, (1) present (Fig 4D1).
4. Head, antenna, antennomeres III, length relative to antennomere IV: (0) as long as (Fig 5A1), (1) at least a 1/5 longer, (2) a 1/4–1/5 shorter (Fig 5F1).
5. Head, vertex, shape: (0) slightly depressed to almost flat (Fig 6C1), (1) strongly depressed (Fig 7L).
6. Head, frons, distance between antennal sockets relative to socket width: (0) 1/2 as wide (Fig 7I), (1) 1/3–1/4 as wide (Fig 7J), (2) as wide (Fig 7K).
7. Head, labrum, connection to frons: (0) connate by median 1/3 (Fig 7I), (1) connected by membrane throughout (Fig 7L), (2) completely obliterate (Fig 7K).
8. Head, labrum, anterior margin, shape (dorsal view): (0) straight to weakly emarginate (Fig 7I), (1) deeply indented (Fig 7M), (2) rounded.
9. Head, mandibles, shape: (0) overlapping, (1) reduced, not overlapping (Fig 7Q–7V).
10. Head, mandible, apex, shape: (0) mildly blunt, (1) acute (Fig 7Q), (2) knob-like.
11. Head, mandible, stylet, length relative to base: (0) almost as long (Fig 7V), (1) almost 1/3 as long (Fig 7S), (2) almost 1/5 as long (Fig 7R).
12. Head, maxilla, palp, palpomere IV, shape: (0) evenly acuminate (Fig 7I), (1) parallel-sided up to apical 5th, then abruptly convergent (Fig 7J).
13. Head, labium, submentum, shape: (0) as wide as long (Fig 7O), (1) 2–3 × longer than wide (Fig 7N).
14. Head, labium, palp, palpomere III, sides, shape: (0) convergent, (1) divergent (Fig 7P).
15. Head, labium, palp, palpomere III, apex, shape: (0) pointy, (1) almost flat, (2) deeply emarginate (Fig 7N).

#### Thorax.

**Fig 8:**
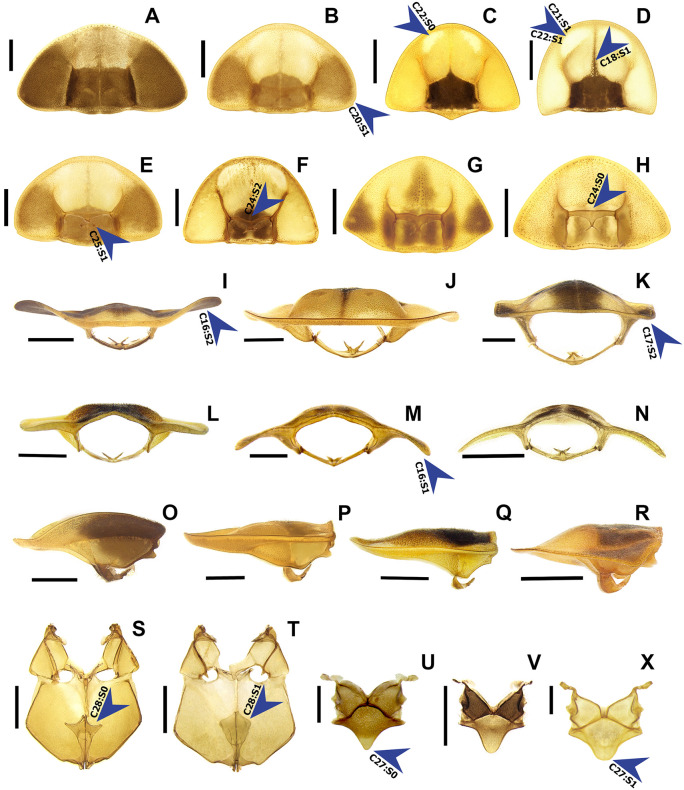
Male. Prothorax: dorsal view **(A–D)**; ventral view **(E–H)**; anterior view **(I–K)**; posterior view **(L–N)**; lateral view **(O–R)**. Metendosternite **(S–T)**. Mesoscutellum, dorsal view (U–X). A, *Lamprocera diluta*. B, E, *Nyctocera sinuaticolle* **comb. nov.** C, *Cratomorphus distinctus*. D, J, *Cratomorphus signativentris*. F, V, *Cratomorphus splendidus*. G, *Aspisoma maculatum*. H, *Aspisoma laetum*. I, O, *Lychnacris flabellata*. K, *Pyractomena* cf. *ecostata*. L, Q, *Cratomorphus leoneli*. M, X, *Aspisoma gentile*. N, T, *Aspisomoides costatum*. R, *Aspisoma lineatum*. S, P, *Cratomorphus albomarginatus*. U, *Tenaspis sinuosa*. Scale bar: 1mm (I–R, U– **X)**, 2mm (A–H, S–T).

**Fig 9:**
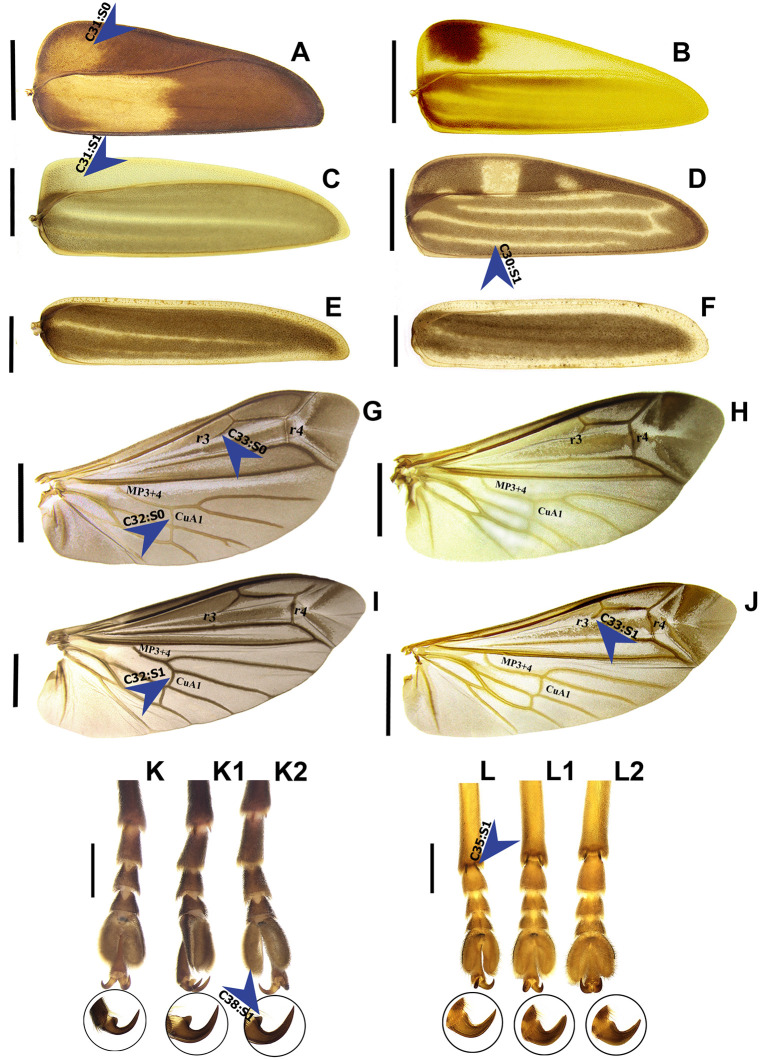
Male. Elytra, ventral view **(A–F)**: A, *Lamprocera diluta*. B, *Aspisoma maculatum*. C, *Aspisomoides bilineatum*. D, *Aspisoma buyssoni*. E, *Pyractomena* cf. *ecostata*. F, *Pyractomena lucifera*. Wing, dorsal view **(G–J)**: G, *Tenaspis sinuosa*. H, *Aspisoma ignitum*. I, *Cratomorphus albomarginatus*. J, *Aspisoma maculatum*. Tarsomeres and detail of tibial spurs **(K–L)**: *Cratomorphus splendidus* (K, proleg; K1, mesoleg; K2, metaleg); *Nyctocera fuscipennis* **comb. nov.** (L, proleg; L1 mesoleg; L2 metaleg). Scale bar: 1mm **(A–F)**, 2mm **(G–L)**.

1. Thorax, pronotum, lateral expansions, shape (frontal view): (0) straight (Fig 8J), (1) bent ventrally (covering hypomeron in lateral view) (Fig 8M), (2) bent dorsally (Fig 8I).
2. Thorax, pronotum, lateral expansion, width relative to disc: (0) almost as wide (Fig 8C), (1) nearly 1/2 as wide (Fig 4K), (2) 2/3–3/4 as wide.
3. Thorax, pronotum, anterior expansion, dorsomedian longitudinal keel: (0) absent, (1) present (Fig 8D).
4. Thorax, pronotum, lateral expansion, posterior angle, shape: (0) straight (Fig 8F), (1) rounded (Fig 8G).
5. Thorax, pronotum, posterior angle, notch: (0) absent (Fig 5A), (1) present (Fig 8B).
6. Thorax, pronotum, anterior expansion, vitreous spots (i.e., ample fusion of punctures): (0) absent (Fig 5A), (1) present (Fig 5D).
7. Thorax, pronotum, anterior expansion, vitreous spots (i.e., ample fusion of punctures), extent: (0) rudimentary (Fig 8C), (1) well developed (Fig 8D).
8. Thorax, pronotum, posterolateral angles, position relative to posterolateral angle of disc: (0) aligned with (Fig 8F), (1) more posterior than (Fig 8G).
9. Thorax, prosternum, anterior margin, medial region, shape: (0) straight (Fig 8E); (1) evenly emarginate throughout; (2) strongly emarginate (Fig 8F).
10. Thorax, prosternum, proendosternite, apex, shape: (0) entire (i.e., not bifid) (Fig 8H), (1) bifid (Fig 8E).
11. Thorax, prosternum, proendosternite, arm length relative to distance between arms: (0) longer (Fig 8H), (1) shorter (Fig 8F).
12. Thorax, mesoscutellum, posterior margin, shape: (0) pointed (Fig 8U), (1) rounded (Fig 8X), (2) truncate.
13. Thorax, metendosternite, region anterior to furcal arms, curvature: (0) strongly concave (Fig 8S), (1) straight to convex (Fig 8T).
14. Thorax, metendosternite, shape: (0) divergent up to basal 1/2, then convergent, (1) divergent up to basal 1/4, then convergent.
15. Thorax, elytron, dorsal surface, longitudinal keels: (0) absent (Fig 9A), (1) present (Fig 9D).
16. Thorax, elytron, humerus, lateral expansion, width relative to disc: (0) almost as wide (Fig 9A), (1) nearly 1/2 as wide (Fig 9D), (2) 1/3 as wide (Fig 9F).
17. Thorax, wing, position of MP3 + 4 split relative to the CuA1 crossvein: (0) more apical (Fig 9G), (1) more basal (9I).
18. Thorax, wing, r3 length relative to r4: (0) almost equal (Fig 9G), (1) 1/2–1/3 as long (Fig 9J).
19. Thorax, wing, radial cell segment between r3 and r4, width relative to r4 length: (0) as long as, (1) at least a 1/5 wider.
20. Thorax, leg, proleg, tibial spurs, count: (0) one (Fig 9K), (1) two (Fig 9L), (2) zero.
21. Thorax, leg, proleg, anterior claw, tooth: (0) absent, (1) present (Fig 9K).
22. Thorax, leg, mesoleg, anterior claw, tooth: (0) absent (Fig 9L1), (1) present (Fig 9K1).
23. Thorax, leg, metaleg, anterior claw, tooth: (0) absent (Fig 9L2), (1) present (Fig 9K2).

#### Abdomen.

**Fig 10:**
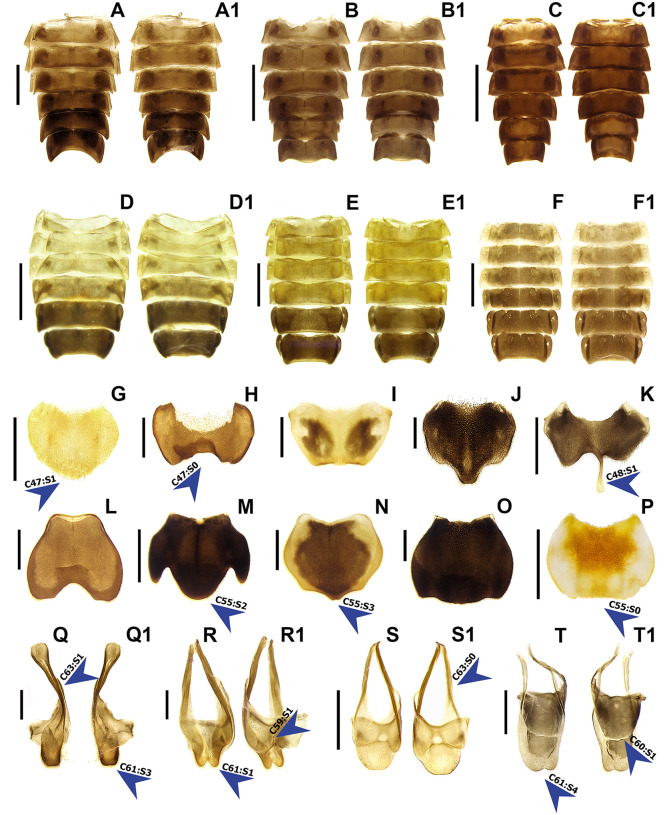
Male. Abdomen: dorsal view **(A–F)**; ventral view (A1–F1). Sternum VIII **(G–K)**; pygidium **(L–P)**; sternum IX **(Q–T)**; syntergite (Q1–T1). A, A1, Q, Q1, *Lamprocera diluta*. B, B1, K, *Cratomorphus splendidus*. C, C1, *Cratomorphus distinctus*. D, D1, *Aspisoma* *ignitum*. E, E1, *Aspisoma maculatum*. F, F1, *Aspisomoides costatum*. G, *Cratomorphus bifenestratus*. H, L, *Lychnacris flabellata*. I, N, *Cratomorphus signativentris*. J, O, *Cratomorphus distinctus*. M, R, R1, *Cratomorphus albomarginatus*. P, *Aspisoma lineatum*. S, S1, *Aspisoma laetum*. T, T1, *Pyractomena* cf. *ecostata*. Scale bar: 0,5mm **(G–P)**; 1mm **(Q–T)**; 2mm **(A–F)**.

**Fig 11:**
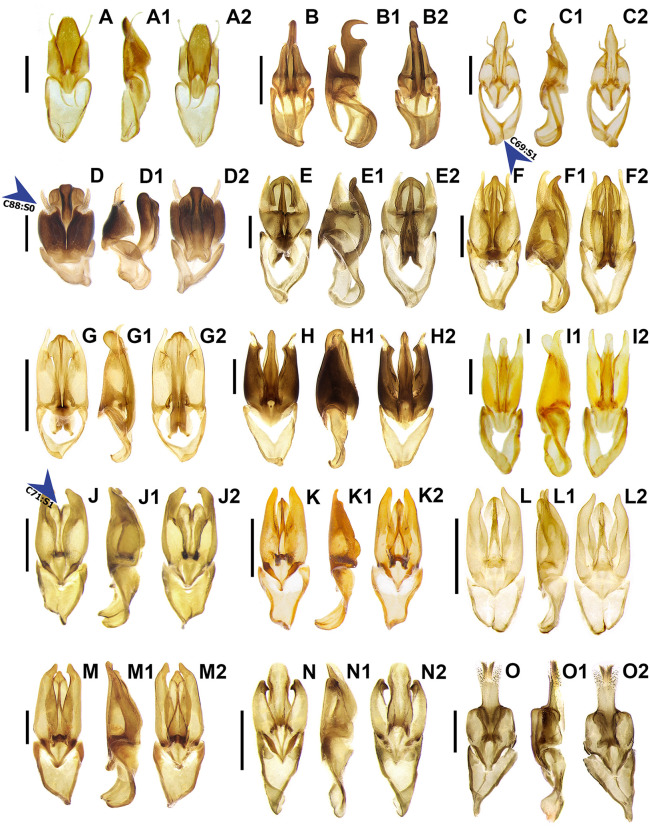
Aedeagus: dorsal view (A–O), lateral view (A1–O1), ventral view (A2–O2). A, A1, A2: *Tenaspis sinuosa*. B, B1, B2: *Lychnacris flabellata*. C, C1, C2: *Lucio pictum*. D, D1, D2: *Erythrolychnia bipartita*. E, E1, E2: *Cratomorphus splendidus*. F, F1, F2: *Cratomorphus signativentris*. G, G1, G2: *Cratomorphus bifenestratus*. H, H1, H2: *Cratomorphus distinctus*. I, I1, I2: *Cratomorphus leoneli*. J, J1, J2: *Aspisoma ignitum*. K, K1, K2: *Aspisoma lineatum*. L, L1, L2: *Aspisoma laetum*. M, M1, M2: *Aspisoma buyssoni*. N, N1, N2: *Aspisomoides costatum*. O, O1, O2: *Pyractomena* cf. *ecostata*. Scale bar: 0.5mm **(A–O)**.

**Fig 12:**
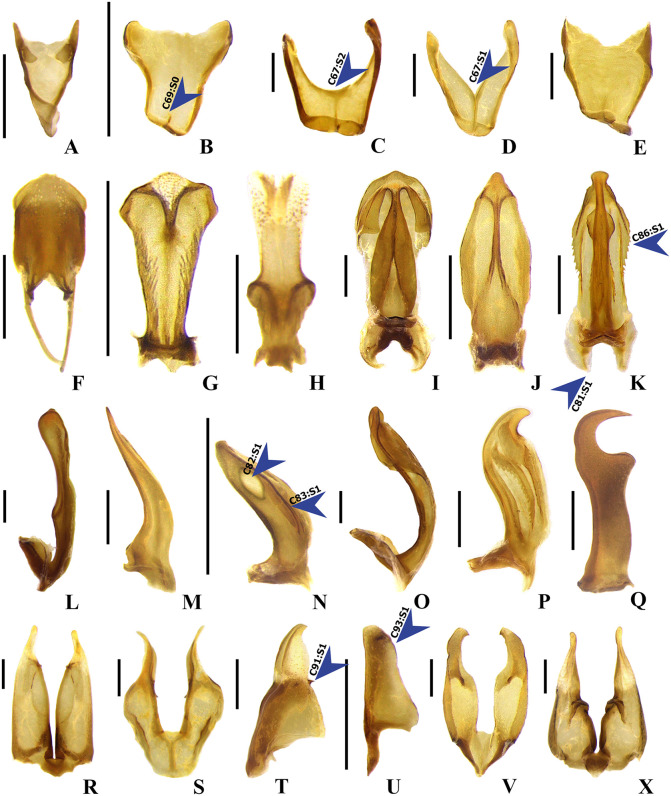
Aedeagus, details of: phallobase (A–E); phallus, ventral view (F–K); phallus, lateral view (L–Q); parameres: ventral view (R, S, V, X) and lateral view (U, V). A, *Aspisomoides costatum*. B, *Aspisoma maculatum*. C, *Nyctocera sinuaticolle* **comb. nov***.* D, *Cratomorphus dorsallis*. E, *Aspisoma ignitum*. F, *Tenaspis chamelensis*. G, *Aspisoma ignitum*. H, *Pyractomena* cf. *ecostata*. I, *Cratomorphus albomarginatus*. J, *Aspisoma buyssoni*. K, *Cratomorphus dorsalis*. L, *Cratomorphus bifenestratus*. M, *Lucio pictum*. N, *Aspisoma maculatum*. O, *Cratomorphus splendidus*. P, T, *Cratomorphus dorsalis*. Q, *Lychnacris flabellata*. R, *Cratomorphus bifenestratus*. S, *Nyctocera fuscipennis* **comb. nov**. U, *Aspisomoides bilineatum*. V, *Aspisoma ignitum*. X, *Cratomorphus signativentris*. Scale bar: 0.5mm (A–X).

**Fig 13:**
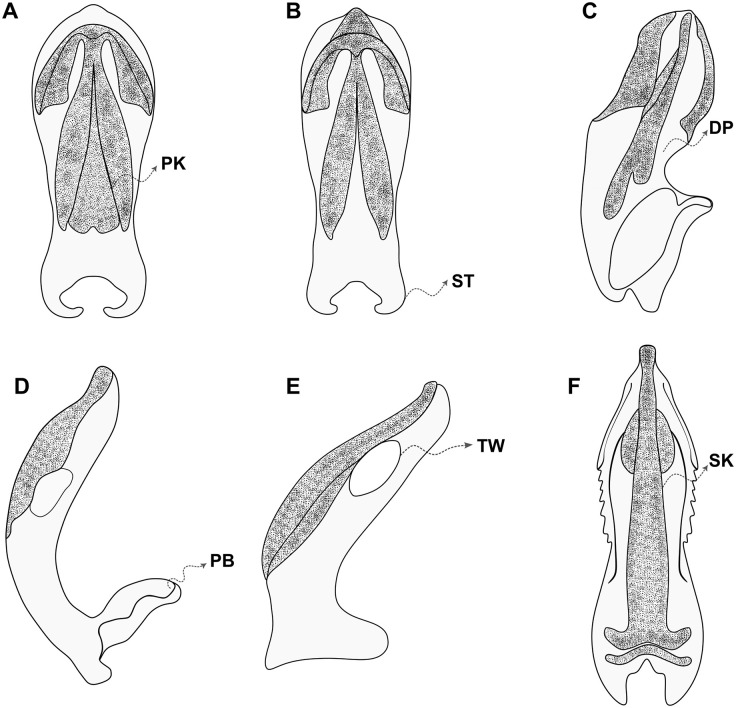
Schematic drawing of the phallus. A:*Cratomorphus splendidus* (dorsal). B: *Cratomorphus albomarginatus* (ventral). C: *Cratomorphus* s*plendidus* (oblique). D: *Cratomorphus albomarginatus* (lateral). E: *Aspisoma maculatum* (lateral). F: *Cratomorphus splendidus* (dorsal). PK (paired keel); ST (struts); DP (dorsal plate); PB (phallic bridges); TW (translucent window); SK (simple keel).

1. Abdomen, terga II–V, posterior angles, shape: (0) acute (Fig 10A), (1) right-angled (Fig 10C).
2. Abdomen, terga VII, posterior margin, central 1/3, shape: (0) emarginate (Fig 10A), (1) bisinuate (Fig 10B), (2) straight (Fig 10C).
3. Abdomen, spiracles, position: (0) ventral, (1) dorsal (Fig 10F).
4. Abdomen, spiracles, diameter relative to sternite length: (0) nearly 1/5 (Fig 10A1), (1) nearly 1/2–1/3 (Fig 10F).
5. Abdomen, sternum VI, lantern: (0) absent (Fig 10A1), (1) present (Fig 10E1).
6. Abdomen, sternum VI, lantern, shape: (0) circular or triangular (occupying 1/3), (1) tripartite, (2) transverse, (3) entire (occupying the whole sternum), (4) bipartite.
7. Abdomen, sternum VII, lantern: (0) absent (Fig 10A1), (1) present.
8. Abdomen, sternum VII, lantern, shape: (0) entire, (1) bipartite (as two lateral circles).
9. Abdomen, sternum VIII, posterior margin, shape: (0) emarginate (Fig 10H), (1) rounded (Fig 10G), (2) almost straight.
10. Abdomen, sternum VIII, posterior margin, projection: (0) absent, (1) present (Fig 10K).
11. Abdomen, sternum VIII, posterior margin, projection, length relative to sternum VIII: (0) rudimentary (mucronate), (1) 1/3–1/5 smaller, (2) nearly half (Fig 10K).
12. Abdomen, sternum VIII, posterior margin, projection, position: (0) central (Fig 10J), (1) offset (Fig 10K).
13. Abdomen, sternum VIII, lateral margins, shape: (0) convergent posteriorly from anterior 1/3 (Fig 10I), (1) rounded (Fig 10G).
14. Abdomen, pygidium, shape (proportion): (0) as wide as long (Fig 10L), (1) wider than long (Fig 10P).
15. Abdomen, pygidium, anterior margin, shape: (0) straight (Fig 10L), (1) emarginate (Fig 10P), (2) indented.
16. Abdomen, pygidium, sides, shape: (0) divergent posteriorly up to 1/2 the length of the pygidium (Fig 10L), (1) rounded (Fig 10N), (2) subparallel.
17. Abdomen, pygidium, posterior margin, central 1/3, shape: (0) straight (Fig 10P), (1) emarginate (Fig 10L), (2) rounded (Fig 10M), (3) mucronate (Fig 10N).
18. Abdomen, pygidium, posterior margin, posterolateral corners, degree of development: (0) rudimentary (Fig 10N), (1) well-developed (Fig 10M).
19. Abdomen, pygidium, posterior margin, indentation, depth: (0) shallow, (1) deep (at least a 1/4 pygidium length).
20. Abdomen, syntergite, connection to sternum IX, length relative to sternum IX: (0) nearly 1/3–1/4 the length, (1) half the length.
21. Abdomen, syntergite, sagittal suture: (0) absent, (1) present (Fig 10R1).
22. Abdomen, syntergite, transverse suture, condition: (0) obliterate, (1) membranous (Fig 10T1), (2) connate.
23. Abdomen, sternum IX, posterior margin, shape: (0) evenly rounded (Fig 10S), (1) indented (Fig 10R), (2) emarginate, (3) truncate (Fig 10Q), (4) sinuose.
24. Abdomen, sternum IX, posterior margin, transversal keel: (0) absent, (1) present.
25. Abdomen, sternum IX, posterior half, sclerotization pattern: (0) entire (Fig 10Q), (1) longitudinally split in two lateral rods (Fig 10R).
26. Abdomen, sternum IX, rods, width of anterior 1/3 relative to apical 2/3: (0) as wide, (1) wider.
27. Abdomen, sternum IX, symmetry: (0) slightly asymmetric (Fig 10R), (1) strongly asymmetric (Fig 10Q).
28. Abdomen, aedeagus, phallobase, lateral margin, apical 1/3, tooth-like projections: (0) absent, (1) present (Fig 12A).
29. Abdomen, aedeagus, phallobase, apex, shape: (0) slightly emarginate (Fig 11B), (1) emarginate v-shaped (Fig 11D), (2) emarginate u-shaped (Fig 11C).
30. Abdomen, aedeagus, phallobase, apical margin, shape: (0) rounded, (1) emarginate.
31. Abdomen, aedeagus, phallobase, sagittal line, extent: (0) not reaching apical margin (Fig 10A), (1) throughout phallobase (Fig 10C).
32. Abdomen, aedeagus, phallus, apex, apical 1/3, curvature (lateral view): (0) slightly curved dorsally (Fig 12L), (1) strongly sinuous (Fig 12Q), (2) almost straight (Fig 12N).
33. Abdomen, aedeagus, phallus, dorsal plate, apex, shape: (0) blunt, (1) clefted, (2) rounded, (3) acute (Fig 12M), (4) emarginate (Fig 12H).
34. Abdomen, aedeagus, phallus, dorsal plate, greater width relative to paramere greater width: (0) a 1/5 wider (Fig 11A), (1) at least a 1/5 narrower (Fig 11C), (2) as wide as.
35. Abdomen, aedeagus, phallus, dorsal plate, phallic bridge (a broad, basal bend of the dorsal plate more or less perpendicular to the parameres): (0) absent, (1) present (Fig 13D).
36. Abdomen, aedeagus, phallus, dorsal plate, base, phallic bridge, curvature (lateral view): (0) over 90 degrees (Fig 12P), (1) under 90 degrees (Fig 12O).
37. Abdomen, aedeagus, phallus, dorsal plate, pattern of sclerotization: (0) evenly sclerotized (Fig 12L), (1) with a central sclerotized shaft and a less sclerotized lateral expansion.
38. Abdomen, aedeagus, phallus, dorsal plate, basal 1/4, shape: (0) dorsally projected, (1) depressed (Fig 8E2), (2) flat (Fig 8I2).
39. Abdomen, aedeagus, phallus, dorsal plate, paired longitudinal keels: (0) absent, (1) present (Fig 13A).
40. Abdomen, aedeagus, phallus, dorsal plate, paired longitudinal keels, extent (dorsal view): (0) complete (Fig 12E2), (1) apical 1/3 (Fig 12G2), (2) middle 1/3, (3) basal 1/3.
41. Abdomen, aedeagus, phallus, dorsal plate, paired longitudinal keels, shape: (0) straight, (1) divergent outwards, (2) apically convergent (Fig 12E2), (3) medially fuse.
42. Abdomen, aedeagus, phallus, dorsal plate, single basal median keel: (0) absent, (1) present (Fig 13F).
43. Abdomen, aedeagus, phallus, dorsal plate, struts: (0) absent, (1) present (visible through the phallobase) (Fig 12A2).
44. Abdomen, aedeagus, phallus, dorsal plate, translucent window: (0) absent, (1) present (Fig 13E).
45. Abdomen, aedeagus, phallus, dorsal plate, lateral keel: (0) absent, (1) present (Fig 13N).
46. Abdomen, aedeagus, phallus, dorsal plate, ventrobasal processes: (0) absent, (1) present.
47. Abdomen, aedeagus, phallus, ventral plate: (0) absent, (1) present (8O2).
48. Abdomen, aedeagus, phallus, dorsal plate, lateral margin, texture: (0) smooth (Fig 12J), (1) serrated (Fig 12K).
49. Abdomen, aedeagus, phallus, ejaculatory duct opening, relative to dorsal plate: (0) subterminal, (1) extending a 1/5 beyond dorsal plate, (2) terminal.
50. Abdomen, aedeagus, paramere, pattern of sclerotization: (0) at least partially membranous (Fig 12X), (1) evenly sclerotized (Fig 12V).
51. Abdomen, aedeagus, paramere, pattern of sclerotization, extent of membranous part: (0) nearly 1/2 the length (Fig 11A), (1) nearly 1/4–1/5 the length (Fig 11D), (2) just the tip.
52. Abdomen, aedeagus, paramere, subapical ventral tooth, presence: (0) absent, (1) present (Fig 11H2).
53. Abdomen, aedeagus, paramere, middle 1/3 ventral, tooth: (0) absent, (1) present (Fig 12T).
54. Abdomen, aedeagus, paramere, subapical ventral tooth, shape: (0) ridge-like (Fig 11E), (1) pointy, (2) wide (tooth-like), (3) rudimentary.
55. Abdomen, aedeagus, paramere, apex, curvature (lateral view): (0) curved ventrally (Fig 11A1), (1) almost straight, (2) curved inwards (Fig 11D), (3) curved outwards.
56. Abdomen, aedeagus (dorsal view), basal projection between parameres, anterior margin, shape: (0) truncated (Fig 9R), (1) acute (Fig 8K), (2) rounded (Fig 9S).
57. Abdomen, aedeagus, connection between parameres: (0) fused, forming a straight line (Fig 12S), (1) evenly approximate towards the base, forming a V (Fig 12V).
58. Abdomen, aedeagus, parameres, orientation relative to phallus: (0) dorsal (Fig 11D1), (1) coplanar.
59. Abdomen, aedeagus, parameres, inner face, subapical region: (0) convex (Fig 12V), (1) grooved.

The above traits were separated in the following groups for downstream analyses. “Signaling” include those of the eyes, antennae, and lanterns (chars. 1–6; 43–46); “terminalia and genitalia” (T + G; chars. 58–97); or “somatic” (chars. 7–42; 47–57).

### 3.2 *Phylogenetic analyses*

All analyses (BI, MP–EW, and IW) agree in finding Cratomorphini polyphyletic, split across four well-supported major groups (Figs 2–3), as follows: (i) most *Cratomorphus* spp. (including the type *C. splendidus*) and *Erythrolychnia bipartita* (BD: 5, BS: 86, SR: 89[K1]–98[K5], PP: 1; Fig 3), heretofore Cratomorphini **sensu nov.,** supported by fifteen synapomorphies, fourteen of which are homoplastic (chars. 5:1, 36:1, 37:1, 38:1, 59:1, 61:1, 69:1, 70:0, 75:1, 76:2, 77:1, 81:1, 82:1, 83:1) and one is non-homoplastic: sternum VI with lantern as a transverse stripe (char. 44:2); (ii) *Cratomorphus* (partim: *C. fuscipennis*, *C. fasciata*, and *C. discorufa*), transferred here with a new species to *Nyctocera* **gen. nov.** (BD: 9, BS: 100, SR: 100 [K1–20], PP: 1) – placed with the Lamprocerini **sensu nov.** – supported by nine homoplastic synapomorphies (chars. 5:1, 6:1, 19:1, 21:1, 27:0, 43:1, 55:0, 60:2, 72:2); (iii) *Cratomorphus* (partim: *C. besckei*, transferred here with a new species to *Bituca* **gen. nov.**) (BD: 6, BS: 99, SR: 98[K3]–99[K1, 5–20], PP: 0.96) supported by nine homoplastic synapomorphies (chars. 5:1, 13:0, 27:0, 31:1, 56:1, 71:2, 72:0, 87:2, 93:0); (iv) and a clade including *Micronaspis* ((*Aspisomoides*, *Pyractomena*) *Aspisoma*), which will be transferred here to Aspisomini **trib. nov.** (BD: 2, BS: 89, SR:84[K1]–93[K10–20], PP: 0.99) supported by two homoplastic synapomorphies (chars. 68:1, 76:2) and two non-homoplastic synapomorphies (chars. 28:1, 29:1).

Aspisomini **trib. nov.** is consistently recovered sister to a broader clade that includes Cratomorphini **sensu nov.**, Lamprocerini **sensu nov.**, Lampyrini, and *Pleotomus*. This broader clade is supported by two homoplastic synapomorphies (chars. 10:1, 93:1) and four non-homoplastic synapomorphies (chars. 9:1, 18:1, 60:1, 87:0), generally showing high support values when homoplasy is accounted for (BD: 4, BS: 51, SR:69[K20]–82[K1], PP: 0.99). The analyses mainly differ whether *Bituca* is sister to Cratomorphini **sensu nov.** (MP–IWK1; no support) or sister to all Cratomorphini **sensu nov.**, Lamprocerini **sensu nov.**, Lampyrini, and *Pleotomus* (MP–EW and MP–IWK3–20; BD for the sister to *Bituca* **gen. nov.**). However, neither of these relationships showed strong statistical support (BS, SR, and PP < 50).

Other stable topologies that are nevertheless less central to the monophyly of Cratomorphini but relevant to the Lampyrinae phylogeny at large are the well-supported clustering of *Pleotomus* with the Lampyrini (BD: 4, BS: 76, SR: 90[K20]–96[K3–5], PP: 1.0), and Lamprocerini **sensu nov.** as sister to *Pleotomus* + Lampyrini (BD: 2, BS: –, SR: < 50[K10–20]–58[K1], PP: 0.59).

Within Cratomorphini **sensu nov.**, two clades were recurrent: (i) the well supported (*C. diaphanus* (*C. splendidus*, *C. albomarginatus*)) (BD: 3, BS: 94, SR:93[K1]–97[K20], PP: 1.0) with four homoplastic synapomorphies (chars. 27:0, 39:0, 43:1, 89:0) and two non-homoplastic synapomorphies (chars. 49:2, 50:1); (ii) and the moderately supported (*C. leoneli*, *C. distinctus*) (BD: 2, BS: 68, SR: 71[K1]–79[K20], PP: 0.77) with one homoplastic synapomorphy (char. 47:1).

The position of *Erythrolychnia* (currently placed in Lucidotini) was sensitive to homoplasy: BI and MPIW with lower K values (1–3) place it nested in *Cratomorphus*, variably clustered with *C. bifenestratus*, *C. splendidus*, *C. albomarginatus*, *C. diaphanus*, *C. dorsalis*, *C. signativentris*, and *C. cossyphinus*. However, MPIW with higher K values (5–20) and MPEW places it sister to *Cratomorphus* **sensu nov.**.

The clade ((*C. splendidus*, *C. albomarginatus*, *C. diaphanus*), *C. bifenestratus*, *C. dorsalis*, *C. signativentris*, *C. cossyphinus*) is recovered in MPEW and MPIW with weak to moderate support in moderate to high values of K (BD: 2, BS: 62, SR: < 50[K1–3]–66[K20], PP: < 0.5). *C. picipennis* is always recovered sister to all species within *Cratomorphus* **sensu nov.** and, when homoplasy is accounted for (BI and MPIWK1–3), also relative to *Erythrolychnia bipartita*. However, support for this placement is weak (BD: 1, BS: < 50, SR: < 50[K3–20]–62[K1], PP: 0.77). The resolution within *Cratomorphus* **sensu nov.** and *Aspisoma* **sensu nov.** was more labile, contingent on the phylogenetic optimality criterion used.

Lamprocerini **sensu nov.** is always recovered as monophyletic but poorly supported (BD: 1, BS: < 50, SR: < 50[K1–20], PP: 0.78), with five homoplastic synapomorphies (chars. 1:0, 6:0, 39:0, 58:0, 71:3). The basal relationships within Lamprocerini are unsteady and contingent on the analysis, where *Lucernuta* and *Tenaspis* alternate as the sister to the stable and moderately supported (*Lychnacris* (*Nyctocera* **gen. nov.** (*Lucio*, *Lamprocera*))) (BD: 2, BS: 79, SR: 73[K1]–82[K10–20], PP: 0.78).

The monophyly of the clade (*Nyctocera* **gen. nov.** (*Lucio*, *Lamprocera*)) (supported by three non-homoplastic synapomorphies: chars. 12:1, 25:1, 65:1; and three homoplastic synapomorphies: chars. 1:1, 24:0, 69:1; BD: 5, BS: 97, SR: 99[K1–20], PP: 1.0) and that of *Nyctocera* **gen. nov.** (supported by the following nine homoplastic synapomorphies: chars. 5:1, 6:1, 19:1, 21:1, 27:0, 43:1, 55:0, 60:2, 72:2; BD:9, BS: 100, SR: 100[K1–20], PP: 1.0) are both stable and well supported (Figs 2–3).

Aspisomini **trib. nov.** has moderate to strong support for *Micronaspis* being sister to the clade ((*Aspisomoides*, *Pyractomena*) *Aspisoma*) (one non-homoplastic synapomorphy: char. 28:1, 29:1; two homoplastic synapomorphies: chars. 68:1, 76:2; BD: 1, BS: 71, SR:72[K20]–76[K1], PP: 0.97). There is robust support for the *Aspisomoides + Pyractomena* clade (BD: 2, BS: 83, SR: 92[K20]–94[K3–5], PP: 0.99) supported by three homoplastic synapomorphies (chars. 32:1, 42:1, 90:1) and two non-homoplastic synapomorphies (chars. 8:1, 65:1).

All Aspisomini **trib. nov.** genera were recovered as monophyletic across analyses, but their support varied. There was strong support for *Pyractomena* (one non-homoplastic synapomorphy: 71:4, 85:1; three homoplastic synapomorphies: 11:1, 52:0, 83:1; BD: 5, BS: 99, SR: 99[K1–20], PP: 1.0), and for *Aspisomoides* (one non-homoplastic synapomorphy: char. 30:1; four homoplastic synapomorphies: chars. 4:1, 16:1, 19:1, 76:0; BD: 5, BS: 98, SR: 99[K1–20], PP: 1.0). Support for *Aspisoma* (one non-homoplastic synapomorphy: char. 97:1; four homoplastic synapomorphies: chars. 38:1, 59:1, 60:2, 71:2; BD: 2, BS: 83, SR: 67[K1]–89[K10], PP: 0.88) and *Micronaspis* (one non-homoplastic synapomorphy: char. 24:2; three homoplastic synapomorphies: chars. 40:1, 48:1, 70:0; BD: 3, BS: 94, SR: 97[K1, 10–20]–98[K3], PP: 0.78) was overall moderate but weaker on MPIW with lower K values.

Resolution within *Aspisoma* varied by optimality criteria, especially that of its backbone, which was never robustly supported. Two clades concerning the larger *Aspisoma* species were stable and moderately supported, as follows: (*A. ignitum* (*A. buyssoni*, *A. physonotum*, *A. laetum*, *A. lineatum*, *A. stictium*, *A. maculatum*))) (BD: 2, BS: 72, SR: 57[K1]–80[K20], PP: 0.89) supported by five homoplastic synapomorphies (char. 17:0, 24:1, 42:1, 88:1, 79:1); and (*A. buyssoni*, *A. physonotum*, *A. laetum*, *A. lineatum*, *A. stictium*, *A. maculatum*))) (one non-homoplastic synapomorphy: char. 23:1; two homoplastic synapomorphies; chars. 19:1, 82:1; BD: 1, BS: 73, SR: 85[K20]–92[K3], PP: 1.0). The topologies resolving this polytomy including *A. buyssoni*, *A. physonotum*, *A. lineatum*, *A. stictium*, *A. maculatum* were labile and/or lacked statistical support (Figs 2,3).

### 3.3 Terminalia and genitalia traits have higher average character congruence

The three trait groups yield statistically different average character congruences (represented by the Retention index) (Kruskal-Wallis chi-squared = 13.887, df = 2, p < 0.001). Terminalia and genitalia (T + G) traits have higher average character congruence than both Signaling (Dunn’s tests Z-statistic = 3.10, p = 0.002), and Somatic (Z = 2.55, p = 0.016) traits (Fig 25A), and this difference is robust to group-label permutations (SES = 3.8, p < 0.001; and SES = 2.67, p = 0.007, respectively; Fig 25B–25C).

**Fig 14:**
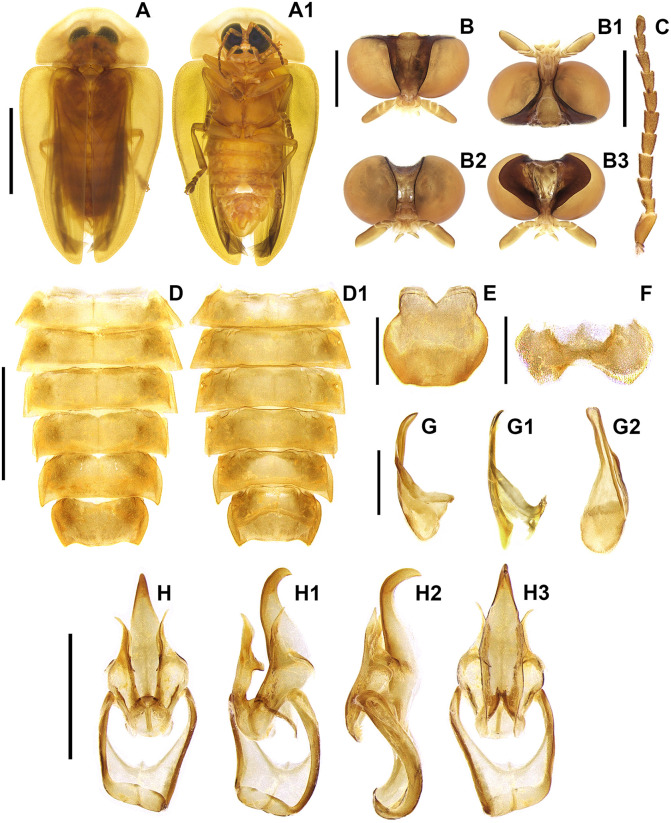
Male. *Nyctocera fuscipennis* **comb**. **nov.**, habitus: A, dorsal view; A1, ventral view. Head: B, dorsal view; B1, ventral view; B2, frontal view; B3, occipital view; C, antenna, lateral view. Abdomen: D, dorsal view; D1, ventral view. E, pygidium. F, sternum **VIII.** Syntergite and sternum IX: G, syntergite; G1, syntergite lateral; G2, sternum **IX.** Aedeagus: H, dorsal view; H1, oblique view; H2, lateral view; H3, ventral view. Scale bar: 2mm (B–C, E–H); 5mm **(A, D)**.

**Fig 15:**
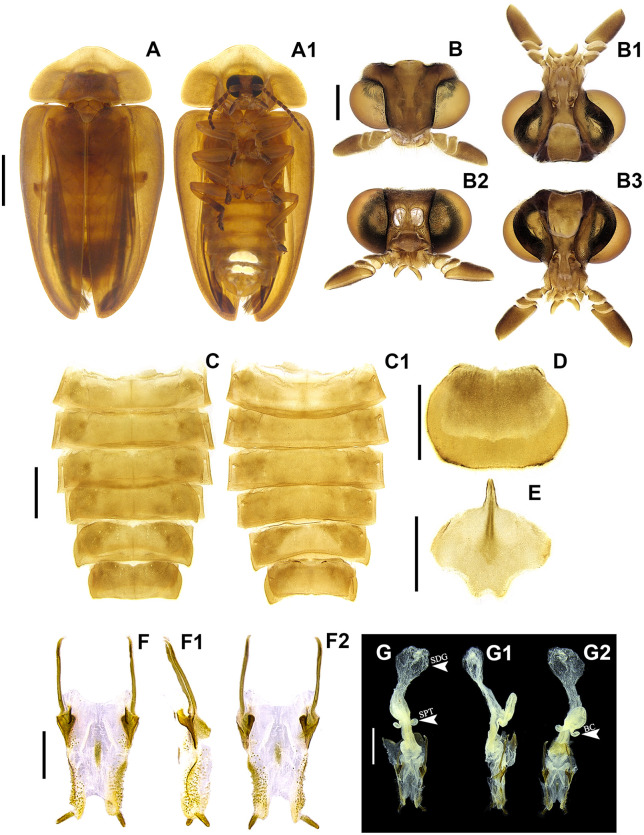
Female. *Nyctocera fuscipennis* **comb**. **nov.,** habitus: A, dorsal view; A1, ventral view. Head: B, dorsal view; B1, ventral view; B2, frontal view; B3, occipital view. Abdomen: C, dorsal view; C1, ventral view. D, pygidium. E, sternum **VIII.** Ovipositor: F, dorsal view; F1, lateral view; F2, ventral view. Internal genitalia: G, dorsal; G1, lateral; G2, ventral. Scale bar: 2mm (C, D, E, F, **G)**; 5mm **(A)**.

**Fig 16:**
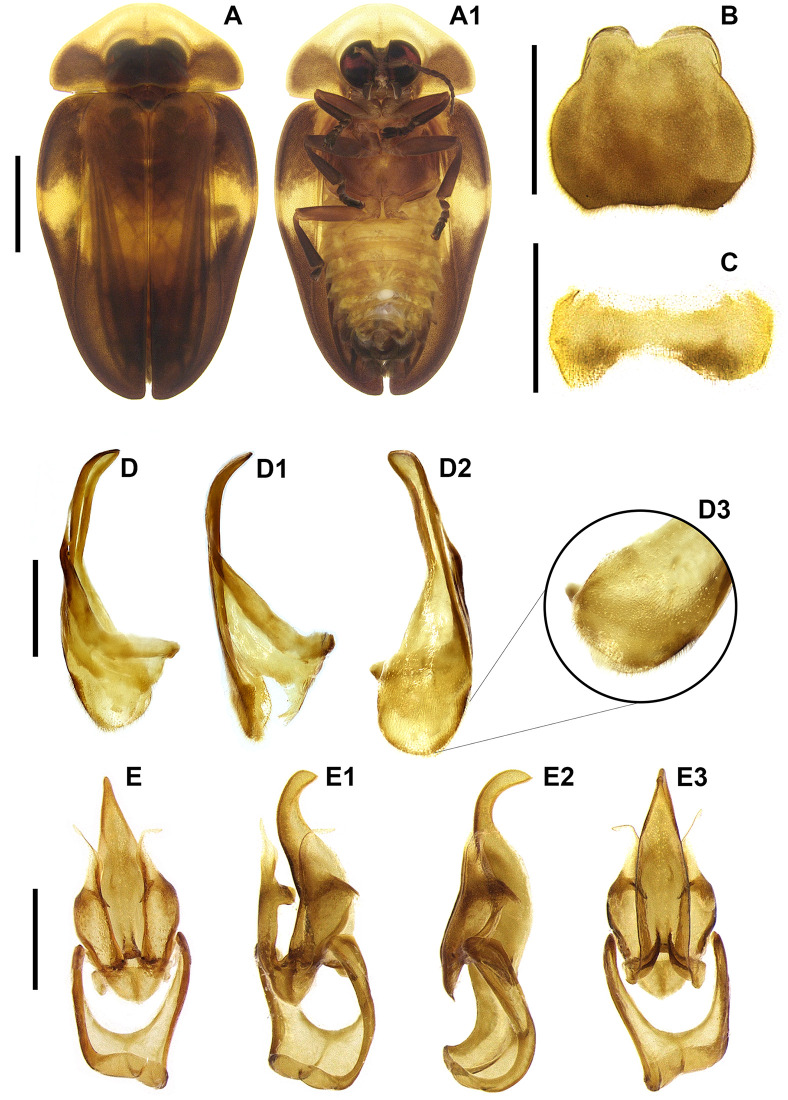
Male. *Nyctocera sinuaticolle* **comb**. **nov.**, habitus: A, dorsal view; A1, ventral view. B, Pygidium. C, sternum **VIII.** Sintergite and sternum IX: D, sintergite; D1, sintergite lateral; D2, sternum IX; D3, details of the posterior region of the sternum **IX.** Aedeagus: E, dorsal view; E1, oblique view; E2, lateral view; E3, ventral view. Scale bar: 1mm (B, C, D, **E)**; 5mm **(A)**.

**Fig 17:**
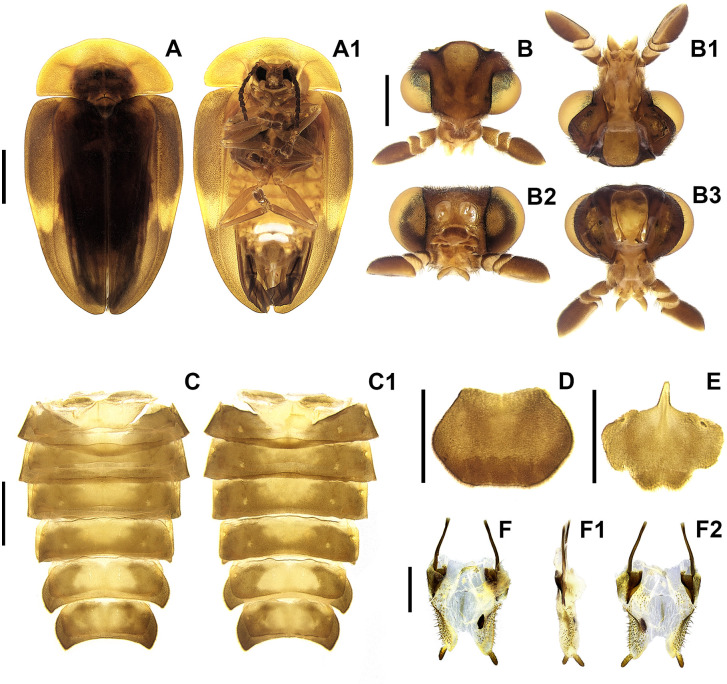
Female. *Nyctocera sinuaticolle* **comb**. **nov.,** habitus: A, dorsal view; A1, ventral view. Head: B, dorsal view; B1, ventral view; B2, frontal view; B3, occipital view. Abdomen: C, dorsal view; C1, ventral view. D, pygidium. E, sternum **VIII.** Ovipositor: F, dorsal view; F1, lateral view; F2, ventral view. Scale bar: 1mm (B, D, E, **F)**; 5mm **(A, C)**.

**Fig 18:**
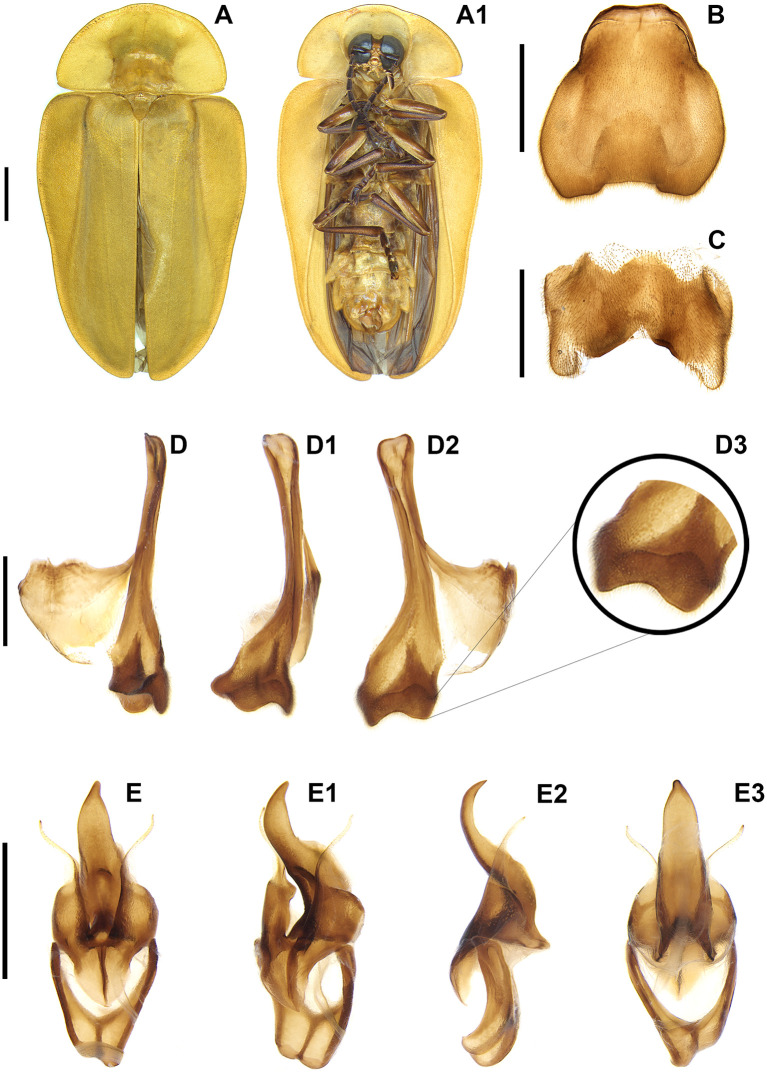
Male. *Nyctocera discorufa* **comb**. **nov.,** habitus: A, dorsal view; A1, ventral view. B, Pygidium. C, Sternum **VIII.** Sintergite and sternum IX: D, sintergite; D1, sintergite lateral, D2, sternum IX; D3, details of the posterior region of the sternum **IX.** Aedeagus: E, dorsal view; E1, oblique view; E2, lateral view; E3, ventral view. Scale bar: 2mm **(B–E)**; 5mm **(A)**.

**Fig 19:**
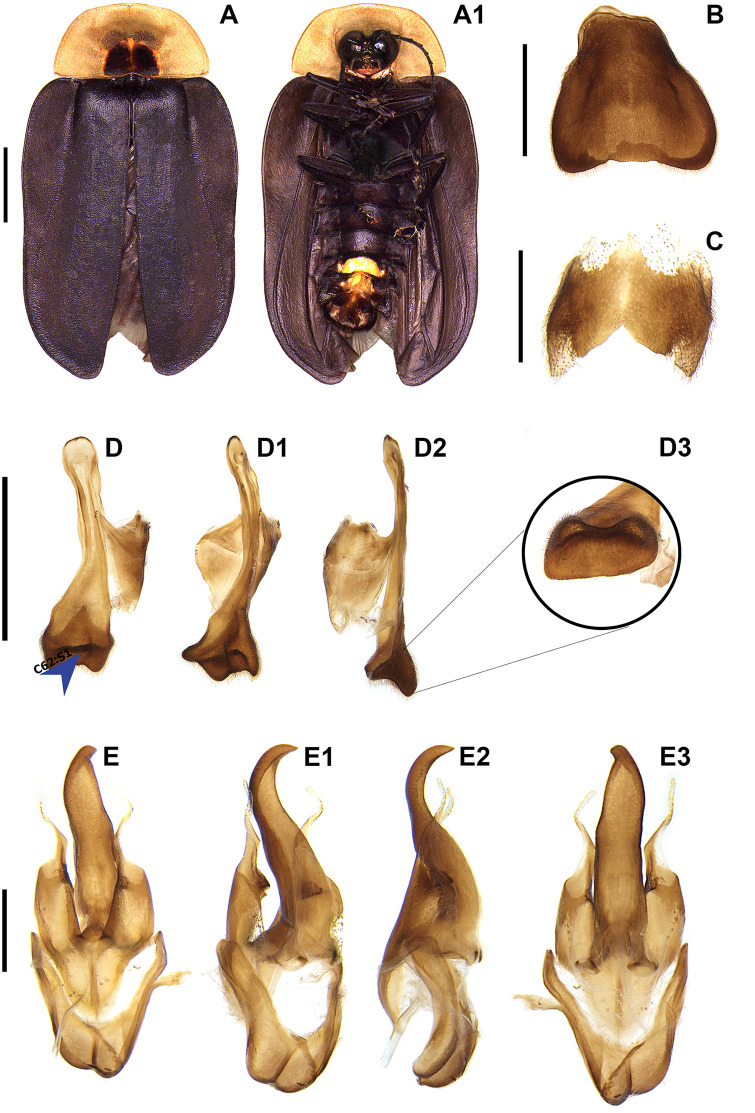
Male. *Nyctocera blattina* **sp**. **nov.,** habitus: A, dorsal view; A1, ventral view. B, pygidium. C, sternum **VIII.** Sintergite and sternum IX: D, sintergite; D1, sintergite lateral; D2, Sternum IX; D3, details of the posterior region of the sternum **IX.** Aedeagus: E, dorsal view; E1, oblique view; E2, lateral view; E3, ventral view. Scale bar: 1mm **(B–E)**; 10mm **(A)**.

**Fig 20:**
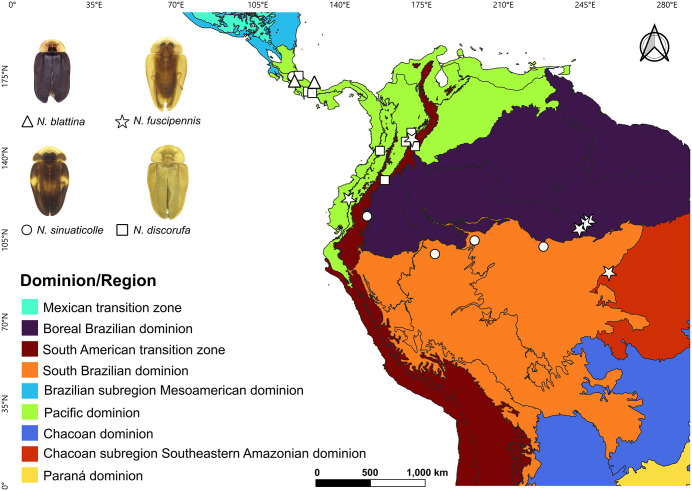
Distribution range of *Nyctocera* gen. nov. on a map superimposed with the biogeographic provinces delimited in Morrone *et al*. (2022).

**Fig 21:**
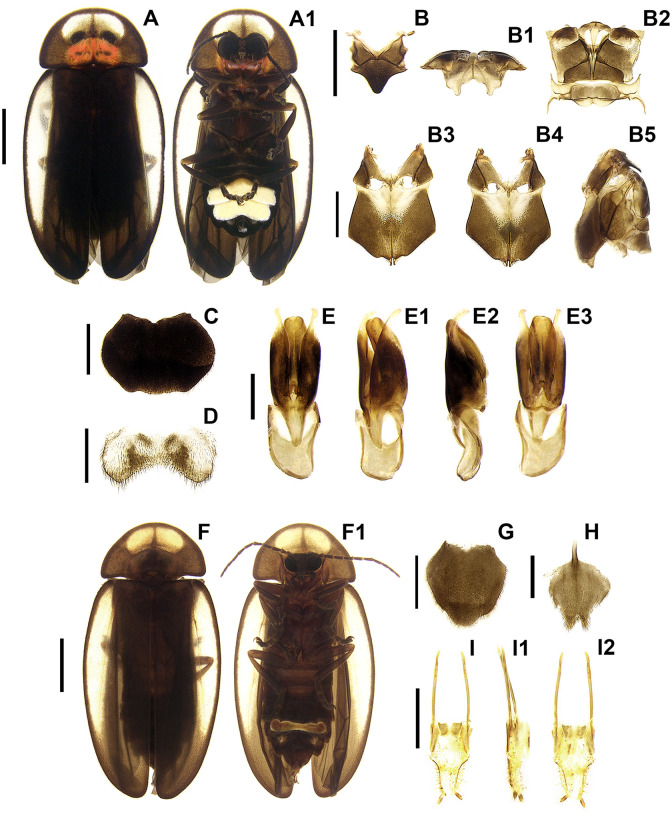
Male and Female. *Bituca besckei* **comb. nov.** Male. Habitus: A, dorsal view; A1, ventral view. Mesoscutellum: B, dorsal view; B1, metanotum, anterior view; B2, metanotum, dorsal view. Pterothorax: B3, ventral view; B4, metendosternite; B5, lateral view; C, pygidium. D, sternum **VIII.** Aedeagus: E, dorsal view; E1, oblique view; E2, lateral view; E3, ventral view. Female. Habitus: F, dorsal view; F1, ventral view. G, pygidium. H, sternum **VIII.** Ovipositor: I, dorsal view; I1, lateral view; I2, ventral view. Scale bar: 1mm (C, E, G, H, **I)**; 2mm (B–B2, B3–B5, **D)**; 3mm **(A, F)**.

**Fig 22:**
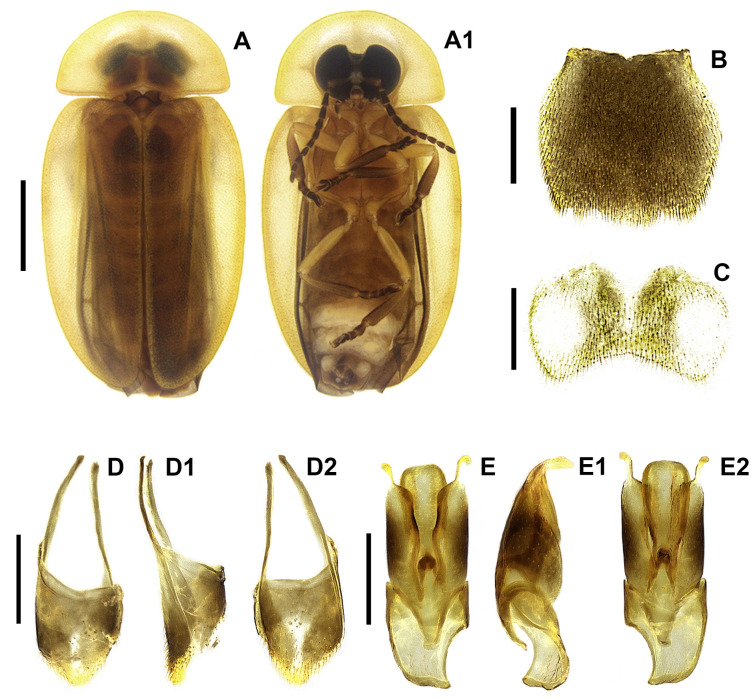
Male. *Bituca miltoni* **sp. nov.,** habitus: A, dorsal view; A1, ventral view. B, pygidium. C, sternum **VIII.** Sintergite and sternum IX: D, sintergite; D1, sintergite (lateral view); D2, sternum **IX.** Aedeagus: E, dorsal view; E1, lateral view; E2, ventral view.

**Fig 23:**
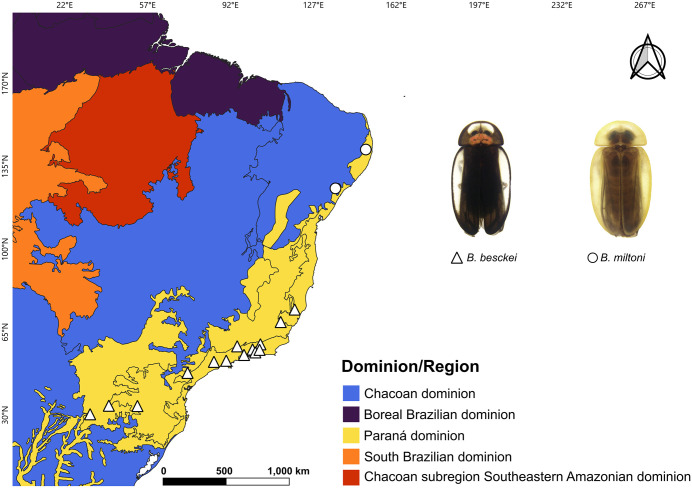
Distribution range of *Bituca* gen. nov. on a map superimposed with the biogeographic provinces delimited in Morrone *et al*. (2022).

**Fig 24:**
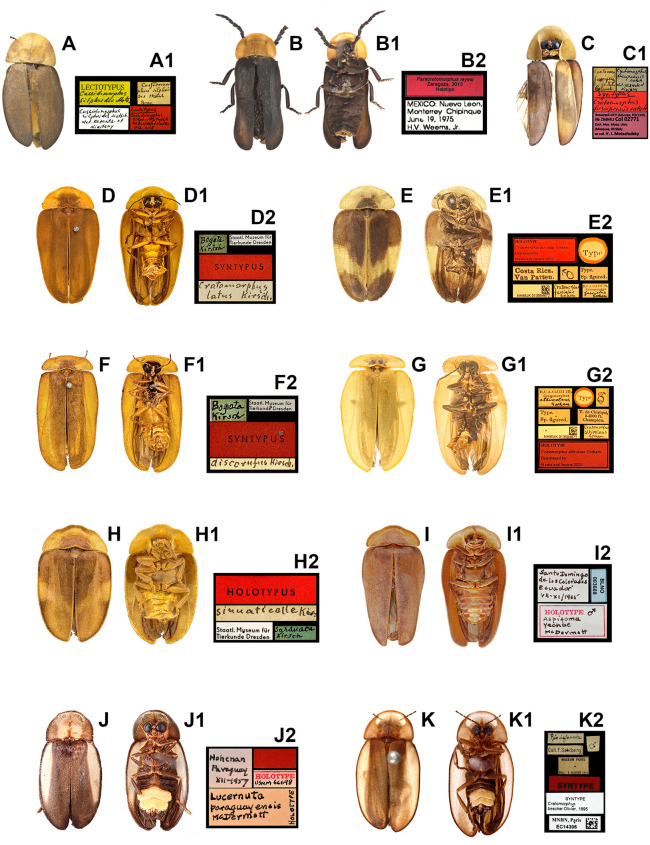
Type specimens of focal taxa examined for this work. Habitus: dorsal view **(A–K)**, ventral view (B1, C, D1–K1). Labels (A1, B2, C1, D2–K2). A: *Cassidomorphus silphoides*, lectotypus. B, B1: *Paracratomorphus reyesi*, holotype. C: *Cratomorphus fuscipennis*, syntype. D, D1: *Cratomorphus latus*, syntype. E, E1: *Cratomorphus fasciatus*, holotype. F, F1: *Cratomorphus discorufus*, syntype. G, G1: *Cratomorphus altivolans*, holotype. H, H1: *Aspisoma sinuaticolle*, holotype. I, I1: *Aspisoma yechae*, holotype. J, J1: *Lucernuta paraguayensis*, holotype. K, K1: *Cratomorphus besckei*, syntype.

**Fig 25:**
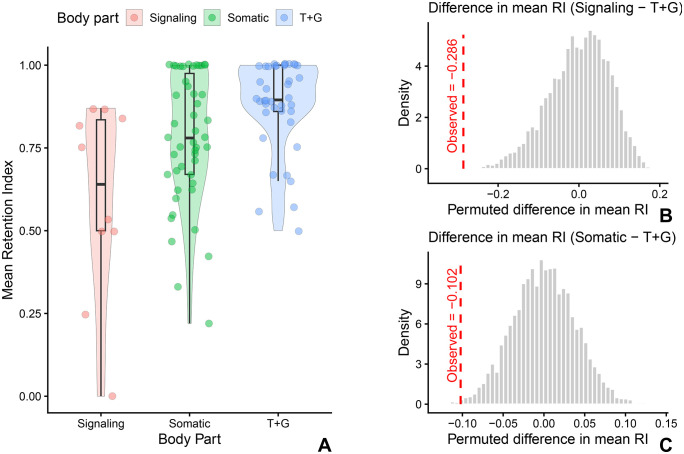
Terminalic and genitalic traits (T + G) are more stable than signaling, or othersomatic traits, as measured from Retention indices averaged across 120 equally most parsimonious trees from the Maximum Parsimony with Equal Weights reconstructions (A). The resulting pairwise average group differences are robust to group-label permutations **(B–C)**. See Material and Methods for analytical details, and Results for statistical reports and significance.

### 3.4 Taxonomy

Lampyridae Rafinesque, 1815

Lampyrinae Rafinesque, 1815

#### Cratomorphini Green, 1959 sensu nov.

**Type genus:** *Cratomorphus* Motschulsky, 1853

**Diagnostic redescription: Head**: Antenna with antennomeres III – IX, cylindrical (Fig 4H1) or serrate; vertex slightly concave (Fig 4J1) to almost flat or strongly depressed; distance between antennal sockets *ca.* 1/3–1/4 of socket width (Fig 7L); clypeus connected by membrane throughout; labrum with anterior margin straight to weakly emarginate (frontal view); mandibles reduced, not overlapping, apex acute, with stylet almost 1/3–1/5 as long as base (Fig 7R, T); maxillary palp 4-segmented, with palpomere IV evenly acuminate (Fig 7I); labial palp with apex of palpomere III almost flat or deeply emarginate. **Thorax**: Pronotum with lateral expansions (frontal view) straight (Fig 8L); dorsal surface with vitreous spots well developed (Fig 8D) or absent; prosternum with anterior margin evenly emarginated throughout (Fig 8F); proendosternite with apex entire (Fig 8F); metendosternite with anterior furcal arms concave, divergent up to its basal half, then convergent (Fig 8S); tibial spur formula: protibia 1 or 2 (Fig 9K), mesotibia 1, metatibia 1; proleg, mesoleg, and metaleg with or without anterior claw tooth (Figs 6K, 6K1, 6K2). **Abdomen**: Spiracles dorsal or ventral (in *C. picipennis*), spiracles with diameter nearly 1/5 of the length of the sternite; sternum VI with (Fig 4J1) or without (Fig 4G1) lantern; sternum VIII with posterior margin projection (Fig 10K) or absent, pygidium with posterior margin rounded, posterior margin with posterolateral corners well-developed (Fig 10M); syntergite with sagittal membranous suture; transverse suture membranous (Fig 10R1); sternum IX with posterior margin indented (Fig 10R) or evenly rounded, posterior half medially divided forming two plates. **Aedeagus**: Phallobase with apical margin emarginate, v-shaped (Fig 12D); basal margin rounded (Fig 11F), sagittal line throughout phallobase (Fig 12D); phallus with apical 1/3 slightly curved (Fig 12P), apical margin blunt with lateral margin smooth or serrated (Fig 12I, K), dorsal plate with phallic bridge curved to the base or curved to the apex (i.e., synapomorphy) (Figs 12L–P), apical margin rounded, with paired longitudinal keels apically convergent (Figs 12I, 13A), with struts visible through the phallobase (Fig 11E), with translucent window (Fig 13E) and lateral keel (Fig 12N), phallus with lateral margin smooth or serrated (Figs 12I, 12K); paramere with membranous part comprising nearly 1/2–1/4 of its length (Fig 12X), bearing subapical ventral teeth (Fig 11N), and a ventral tooth on its middle 1/3 (Fig 9T), apex curved inwards (lateral view) or almost straight (Fig 12T), anterior margin basal projection truncated or rounded.

**Distribution:** New World (Nearctic and Neotropical).

**Remarks:** Cratomorphini **sensu nov.** differs from other tribes of Lampyrinae by the following genitalic traits: dorsal plate of phallus with a phallic bridge curved to the base or curved to the apex (non-homoplastic synapomorphy) (Figs 9L, 9O, 9P; Fig 10D), with a translucent window (Fig 13E), with paired longitudinal keels, and a lateral keel (Fig 13); and phallic struts visible through the phallobase. *Erythrolychnia* and *Cratomorphus* **sensu nov.** have many similar characters related to terminalia and genitalia (see discussion). However, it is possible to distinguish *Erythrolychnia* from *Cratomorphus* **sensu nov.** by the following combination of traits: vertex slightly concave to almost flat (strongly depressed in *Cratomorphus* **sensu nov.**); pronotum without vitreous spots (present in *Cratomorphus* **sensu nov.**); wing with MP3 + 4 split more apical relative to the CuA1 crossvein (more basal in *Cratomorphus* **sensu nov.)**; proleg, mesoleg, and metaleg without anterior claw tooth (present in *Cratomorphus* **sensu nov.**); sternum IX with posterior margin evenly rounded (indented in *Cratomorphus* **sensu nov.**); dorsal plate with paired longitudinal keels straight (paired longitudinal keels apically convergent in *Cratomorphus* **sensu nov.**).

The status of *Paracratomorphus* and *Cassidomorphus* in Cratomorphini deserves further scrutiny. *Cassidomorphus* is represented by a single species, *C. silphoides* Motschulsky, 1853*,* from Brazil, and is known only by a female lectotype [13]. Furthermore, the original description is insufficient to determine its placement among lampyrids, and no other specimen was found despite an intensive search across several entomological collections (see Methods). *Cassidomorphus silphoides* may refer to a very rare species that has never been seen since 1853. However, we refrain from any taxonomics conclusions and until more specimens are found we tentatively maintain *Cassidomorphus* in Cratomorphini.

**Checklist of Cratomorphini genera**

*Cratomorphus* Motschulsky, 1853

*Cassidomorphus* Motschulsky, 1853

*Erythrolychnia* Motschulsky, 1853

*Paracratomorphus* Zaragoza, 2013

Key to Cratomorphini **sensu nov.** genera based on males (*Cassidomorphus* not included since it is only known from the female holotype)

1. Antennomeres III–IX serrate; vertex convex; sternum VI without lantern *Paracratomorphus*

1’. Antennomeres III–IX, cylindrical; vertex slightly concave to almost flat or strongly depressed (Fig 4H1); sternum VI with lantern (Fig 4I) 2

2. Vertex slightly concave to almost flat; pronotum without vitreous spots; wing with MP3 + 4 split more apical relative to the CuA1 crossvein (Fig 9G); proleg; mesoleg and metaleg without anterior claw tooth; sternum VIII without posterior margin projection; sternum IX with posterior margin evenly rounded; phallus, dorsal plate with paired longitudinal keels straight *Erythrolychnia*

2’. Vertex strongly depressed; pronotum with vitreous spots well developed (Fig 5B); wing with MP3 + 4 split more basal relative to the CuA1 crossvein (Fig 9I); proleg, mesoleg and metaleg with anterior claw tooth (Fig 6K); sternum VIII with (Fig 10K) or without (Fig 10G) posterior margin projection; sternum IX with posterior margin indented (Fig 7R); phallic dorsal plate with paired longitudinal keels apically convergent or straight rt *Cratomorphus*

#### *Cratomorphus* Motschulsky, 1853 sensu nov.

**(Fig 1I–L, 1I1–L1; 2A–2D, 2A1–2D1)**

**Type species** *Photinus fabricii* Guérin-Méneville, 1844 [= *Cratomorphus splendidus* (Drury, 1773)], by original designation.

**Diagnostic redescription: Head**: antenna with antennomeres III–IX, cylindrical, antennomere III as long as antennomere IV; vertex strongly depressed (7L). **Thorax**: Pronotum with lateral expansions (frontal view) straight (Fig 8L); dorsal surface with vitreous spots well developed (Fig 8D); wing with MP3 + 4 split more basal relative to the CuA1 crossvein; R3 almost as long as R4 (Fig 9I); prosternum with anterior margin evenly emarginate throughout (Fig 8F); proendosternite with apex entire (Fig 8F); metendosternite with region anterior to the furcal arms concave, divergent up to basal half, then convergent; Tibial spur formula: protibia 1 or 2, mesotibia 1, metatibia 1 (Fig 9K–K2); proleg, mesoleg, and metaleg with anterior claw tooth (Fig 6K–K2). **Abdomen**: Spiracles dorsal or ventral (in *C. picipennis*); sternum VI with a transverse and anteriorly sinuous lantern; sternum VIII with posterior margin projection (Fig 7K), pygidium with posterior margin rounded (Fig 7M), posterior margin of pygidium with deep indentation (extending at least 1/4 of the pygidium length) (Fig 10K); syntergite with sagittal membranous suture, anterior transverse suture membranous (Fig 10R); sternum IX with posterior margin indented, posterior half medially divided forming two plates (Fig 10R1). **Aedeagus**: phallobase with apical margin emarginate, v-shaped (Fig 12D), basal margin rounded, sagittal line throughout phallobase (Fig 12D); phallus with apical 1/3 slightly curved (Fig 12P), apical margin blunt with lateral margin smooth or serrated (Fig 12I, K), dorsal plate with phallic bridge curved towards the base or towards the apex (non-homoplastic synapomorphy) (Fig 12L, O), apical margin round, with paired longitudinal keels apically convergent (Fig 12I) or straight (Fig 12K) with struts visible through the phallobase (Fig 11G) with translucent window and lateral keel (Fig 12N); paramere with membranous part comprising nearly 1/2–1/4 of its length, bearing a subapical ventral tooth (Fig 12R), and a ventral tooth on middle 1/3, apex curved inwards (lateral view) or outwards.

**Distribution:** Neotropical (Caribbean, Central and South America).

Remarks: Motschulsky [65] originally designated *Photinus fabricii* Guérin-Méneville, 1844 as the type species for *Cratomorphus*. However, *P. fabricii* was later identified as a junior synonym of *Lampyris splendida* Drury, 1773 (now *Cratomorphus splendidus*). In accordance with the Principle of Priority [66, Art. 23], *C. splendidus* is the valid name for this taxon. For convenience, the valid name is used hereafter. Based on our analyses, four of the 40 *Cratomorphus* species (*C. fuscipennis, C. fasciatus*,*C. discorufus,* and *C. besckei*) were transferred to two new genera: *Nyctocera* **gen. nov.** and *Bituca* **gen. nov.** After studying the type materials, the following nomenclatural acts are proposed: *Cratomorphus latus* Kirsch, 1865 = *N. fusipennis* **comb. nov.**, and *C. altivolans* Gorham, 1884 = *N. discorufa* **comb. nov**. Upon reviewing the type material of *C. vittatus* Kirsch, 1865, we suggest it may belong to *Nyctocera* **gen. nov.**. However, as no material suitable for dissection is currently available, no formal nomenclatural changes will be taken until a more detailed review of this species is conducted.

*Checklist of Cratomorphus* **sensu nov.**

*C. aequalis* E. Olivier, 1895

*C. albomarginatus* (Laporte, 1840)

*C. anitae* Zaragoza, 1996

*C. ayalai* Zaragoza, 1996

*C. bifenestratus* Gorham, 1880

*C. castaneus* E. Olivier, 1909

*C. cinctipennis* E. Olivier, 1911

*C. concolor* (Perty, 1830)

*C. cossyphinus* (Perty, 1830)

*C. diaphanus* (Germar, 1824)

*C. dilutus* (E. Olivier, 1907)

*C. distinctus* E. Olivier, 1895

*C. dorsalis* (Gyllenhal, 1817)

*C. elevatus* E. Olivier, 1896

*C. frankeae* Bohórquez, 1993

*C. gemellus* Bohórquez, 1993

*C. gorhami* E. Olivier, 1911

*C. halffteri* Zaragoza, 2012

*C. hoffmannae* Zaragoza, 1996

*C. huautlaensis* Zaragoza, 1996

*C. leoneli* Lima, Da Silveira, Fonseca & Zaragoza-Caballero, 2021

*C. limai* Zaragoza-Caballero, Domínguez–León & González–Ramírez, 2021

*C. minutus* (Pic, 1930)

*C. ovatus* Gorham, 1884

*C. parmatus* Gorham, 1880

*C. pellucens* Kirsch, 1865

*C. picipennis* Gorham, 1881

*C. ramirezi* Zaragoza, 1996

*C. rectus* E. Olivier, 1911

*C. rodriguezae* Zaragoza, 1996

*C. signativentris* E. Olivier, 1895

*C. splendidus* (Drury, 1773)

*C. subcostatus* (Guérin–Méneville, 1855)

#### Aspisomini Lima & Silveira, trib. nov.

(Figs 4E–4L, 2E1–2L1; 3A–3I, 3A1–3I1)

**ZooBank LSID** urn:lsid:zoobank.org:act:CEB32CC8-E04A-4CB3-B190-950EA0D137F2

**Type genus:** *Aspisoma* Laporte, 1833

**Diagnostic description: Head**: antenna with antennomeres III–IX, cylindrical (Fig 4I1); vertex slightly concave to almost flat; distance between antennal sockets almost as wide as the socket width (Fig 7K); clypeus completely obliterated or connected by membrane throughout (Fig 7M); labrum with anterior margin straight to weakly emarginate or indented (frontal view) (Fig 7M); mandibles reduced, not overlapping; apex acute, with stylet subequal in length to the base (Fig 7U); maxillary palp 4-segmented, with palpomere IV evenly acuminate (Fig 7M); labial palp 3-segmented; palpomere III with apex deeply emarginate (Fig 7K). **Thorax**: Pronotum with lateral expansions (frontal view) straight or bent ventrally (covering hypomeron in lateral view); lateral expansion nearly as wide as the disc, or 1/3–1/4 narrower (Fig 8F, F), with posterior margin straight (Fig 8F) or rounded; posterolateral angles symmetrical, either aligned (Fig 6F) in position or more posterior relative to posterolateral angles of the disc (Fig 8G); prosternum with anterior margin straight, ranging from evenly emarginate throughout to strongly emarginate; proendosternite with apex entire; metendosternite with region anterior furcal arms convex; arms divergent up to basal 1/4, then convergent; Tibial spur formula: protibia 1, mesotibia 1, metatibia 1; proleg, mesoleg, and metaleg with anterior claw tooth. **Abdomen**: Abdominal spiracles dorsal, spiracles with diameter nearly 1/5 or nearly 1/2–1/3 the length of the sternite; sternum VI with lantern (Fig 5F1); sternum VIII with posterior margin emarginate (Fig 5I1); pygidium with posterior margin medially straight or emarginate (Fig 10P); posterior margin with posterolateral corners reduced (Fig 10P); syntergite with sagittal membranous suture (Fig 10S1); transverse suture membranous or connate (Fig 10R1); sternum IX with posterior margin evenly rounded, medially emarginate to sinuose (Fig 10S1, 10T1). **Aedeagus**: Lateral margin of the phallobase with apical 1/3 either with (Fig 12A) or without tooth-like projections; apical margin slightly emarginate, basal margin rounded or emarginate, sagittal line not reaching apical margin (Fig 12B); dorsal plate of phallus with apical 1/3 nearly straight (Fig 12N), apical margin variable in shape, either blunt, rounded, acute, or emarginate (Fig 12G, 12H, 12J); with lateral margin smooth (Fig 12J); basal 1/4 flat (Fig 12G), evenly sclerotized or with a central sclerotized shaft and a less sclerotized lateral expansion; paired longitudinal keels entire up to its middle and basal 1/3; aedeagus straight, divergent outwards, apically convergent and medially fused, with or without translucent window (Fig 9N); with or without lateral keel (Fig 12N); paramere evenly sclerotized; with (Fig 12V) or without subapical ventral tooth, with or without ventral tooth on its middle 1/3, apex almost straight or curved inwards; anterior margin basal projection acute or rounded, inner face with subapical region convex or grooved (Fig 12V).

**Distribution:** New World (Nearctic and Neotropical).

**Remarks:** Aspisomini **trib. nov.** can be differentiated from the other tribes of Lampyrinae by the following combination of characters: metendosternite anteriorly emarginate, sides divergent up to basal 1/4, then convergent (Fig 8T); and aedeagus with dorsal phallic plate flat on its basal 1/4 (Fig 12N).

*Micronaspis* is easily distinguished from other genera of Aspisomini **trib. nov.** by prosternum overall shape, anterior margin with medial region strongly emarginate (non-homoplastic synapomorphy); posterior margin of sternum VIII with rudimentary projection (mucronate), and apical 1/3 of dorsal plate slightly curved.

*Pyractomena* and *Aspisomoides* share the following traits: labrum with anterior margin indented (non-homoplastic synapomorphy) (Fig 4M), wing with MP3 + 4 split basal to CuA1 (Fig 9I); phallobase laterally indented (non-homoplastic synapomorphy) (Fig 12A), and paramere with a subapical ventral tooth. However, *Pyractomena* differs from *Aspisomoides* by the phallus bearing both a ventral and a dorsal plate, which is apically emarginate (both non-homoplastic synapomorphies), and the lack of raised elytral costae.

*Aspisoma* can be differentiated from other genera by the following set of traits: pronotum with posterolateral angles aligned with or more posterior to those of disc (Fig 8H); anterior claw of the metaleg with tooth; posterior margin of the pygidium with central 1/3 medially straight or emarginate; syntergite with sagittal membranous suture (Fig 7S1); syntergite with transverse suture connate (Fig 10S1); dorsal plate with apical margin round or acute; inner face of the parameres with subapical region grooved. When examining the holotypes and original descriptions of *Aspisoma sinuaticolle* Kirsch, 1873 and *Aspisoma yechae* McDermott, 1966, we concluded that both should be transferred to *Nyctocera* **gen. nov.** (Fig 14–19). The first is considered a synonym of  *Cratomorphus fasciatus*, here in transferred to *Nyctocera* as *N. sinuaticolle* **comb. nov.** (Fig 16–17) and the second is a synonym of *N. fuscipennis* **comb. nov.** (Fig 14–15).

**Checklist of Aspisomini genera**

***A****spisoma* Laporte, 1833

*Aspisomoides* Zaragoza-Caballero, 1995

*Micronaspis* Green, 1948

*Pyractomena* Melsheimer, 1846

Key to **Aspisomini trib. nov.** genera based on males

1. Frontoclypeal suture connected by membrane throughout (Vaz *et al.* [5]: 69, Fig 8E; prosternum with anterior margin strongly emarginate; posterior margin of sternum VIII with a medial projection; apical 1/3 of phallic dorsal plate slightly curved ventrally; dorsal plate without paired longitudinal keels; paramere with apex almost straight; basal projection of paramere with anterior margin rounded *Micronaspis*

1’. Frontoclypeal suture completely obliterated (Fig 7K); prosternum with anterior margin straight or evenly emarginate throughout (Figs 8F, 8H); posterior margin of sternum VIII without a medial projection; apical 1/3 of phallic dorsal plate almost straight (Fig 12N); dorsal plate with paired longitudinal keels (Fig 12I); paramere apex curved inwards; basal projection of paramere with anterior margin acute (Fig 12V) 2

2. Metaleg, anterior branch of claw with a tooth; anterior margin of labrum straight to weakly emarginate; syntergite with a sagittal membranous suture (Fig 10S1); syntergite without a transverse connate suture; anterior margin of phallic dorsal plate round or acute (Figs 12G, 12J); phallobase without tooth-like projections (Fig 12B) *Aspisoma*

2’. Metaleg, anterior branch of claw without a tooth; anterior margin of labrum indented; syntergite without sagittal membranous suture; syntergite with a transverse connate suture; anterior margin of phallic dorsal plate blunt or emarginate; phallobase with tooth-like projections 3

3. Elytron with raised costae (Figs 5G, 6H); anterior margin of phallic dorsal plate blunt (Fig 8N); phallus without ventral plate (Fig 11N2) *Aspisomoides*

3’. Elytron without raised costae (Fig 4E); anterior margin of phallic dorsal plate emarginate; phallus with ventral plate …….…………………………...…….. *Pyractomena*

#### *Aspisoma* Laporte, 1833.

**Type species:** *Cantharis ignita* Linnaeus 1758: 400, by subsequent designation ([1]; see below).

**Diagnostic redescription.** Prosternum with anterior margin straight or evenly emarginate (Fig 8F–8H); labrum straight to weakly emarginate (Fig 7K, 7M); metaleg claw with anterior branch bearing a tooth (Fig 9K2); syntergite with sagittal membranous suture, lacking transverse connate suture (Fig 10T1); elytra without longitudinal keels (Fig 9A); phallic dorsal plate with paired longitudinal keels (Fig 13A), apical margin rounded (Fig 12L) or acute (Fig 12M); phallus without ventral plate (Fig 11N–N2); phallobase unindented; parameres with apex directed inward and inner face grooved subapically (Fig 12V).

**Remarks.** *Aspisoma* differs from *Aspisomoides* and *Pyractomena* by the toothed metaleg claw (Fig 9L–L2) and the presence of a sagittal membranous suture on the syntergite (Fig 10R1), features absent in the latter two genera. The genus *Aspisoma* Laporte, 1833 was established without a type species. *Cantharis ignita* Linnaeus, 1758, later transferred to *Lampyris* by Linnaeus [66], was included by Laporte in his original list of *Aspisoma* species. Motschulsky [65] used *Nyctophanes* Dejean as an alleged older synonym of *Aspisoma*, but the former is a nomen nudum and thus unavailable. McDermott [1] designated *Lampyris ignita* L. 1967 as the type of *Aspisoma*. Despite the misuse of the original combination and year of publication, there is no doubt that he was referring to *Cantharis ignita* Linnaeus, and should be considered the valid type species of *Aspisoma* in accordance with the provisions of the article 67.7 of the ICZN [67].

#### *Aspisomoides* Zaragoza-Caballero, 1995.

Type species: *Aspidosoma bilineatum* Gorham, 1880: 86, by original designation [18]

**Diagnostic redescription.** Prosternum with anterior margin straight or evenly emarginate (Fig 8F–8H); labrum with anterior margin indented (Fig 7M); metaleg claw without tooth (Fig 9L2); syntergite with transverse connate suture (Fig 10S1), lacking sagittal membranous suture; elytra with raised longitudinal keels (Fig 9D); phallic dorsal plate blunt anteriorly, with paired longitudinal keels (Fig 13A); phallus lacking ventral plate (Fig 11N–N2); phallobase laterally indented (Fig 12A); parameres with subapical ventral tooth (Fig 11N).

**Remarks.** *Aspisomoides* shares with *Pyractomena* the indented labrum and phallobase bearing tooth-like projections (Fig 12A), but is distinguished by the presence of elytral keels (Fig 9C) and the absence of a ventral phallic plate.

#### *Micronaspis* Green, 1948.

**Type species:** *Micronaspis floridana* Green, 1948: 63, by monotypy.

**Diagnostic redescription.** Prosternum with anterior margin strongly emarginate medially (Fig 8F); labrum with anterior margin straight (Fig 7I); metaleg claw without tooth (Fig 9L2); syntergite without transverse connate suture (Fig 10T1), posterior margin of sternum VIII with rudimentary medial projection (Fig 10I); elytra without longitudinal keels (Fig 9A); phallic dorsal plate with apical 1/3 slightly curved ventrally, lacking paired longitudinal keels; phallus without ventral plate; phallobase unindented; parameres with apex nearly straight and basal projection rounded anteriorly (see Vaz *et al*. [5]).

**Remarks.** *Micronaspis* is unique in the tribe by the strongly emarginate prosternum and mucronate sternum VIII (see Vaz *et al*. [5]), features absent in *Aspisoma, Aspisomoides,* and *Pyractomena.*

#### *Pyractomena* Melsheimer, 1846.

Type species: *Pyractomena lucifera* Melsheimer, 1846: 304 – misspelled as *Pyratomena lucifera*.

**Diagnostic redescription.** Prosternum with anterior margin straight or evenly emarginate (Fig 8F–8H); labrum with anterior margin indented (Fig 7M); metaleg claw without tooth (Fig 9L2); syntergite with transverse connate suture (Fig 10S1), lacking sagittal membranous suture; elytra without longitudinal keels (Fig 9E-9F); phallic dorsal plate apically emarginate, with paired longitudinal keels (Fig 12H); phallus bearing both dorsal and ventral plates (Fig 11O–O2); phallobase laterally indented; parameres with subapical ventral tooth (Fig 11H2).

Remarks. *Pyractomena* is closely related to *Aspisomoides*, sharing the indented labrum (Fig 7M) and phallobase tooth-like projections (Fig 12A), but differs by the absence of elytral keels (Fig 9E–9F) and the presence of a ventral phallic plate.

#### Lamprocerini Olivier, 1907 sensu nov.

**Type genus**: *Lamprocera* Laporte, 1833

**Diagnostic redescription: Head**: antenna with antennomeres III–IX, serrate or cylindrical, with or without single lamellae and double lamellae (Fig 4E–4F); vertex slightly concave to almost flat or strongly depressed (Fig 14B2); distance between antennal sockets nearly 1/3–1/4 of socket width (Fig 14B2); clypeus connate by median 1/3 or connected by membrane throughout (Fig 15B2); labrum with anterior margin straight to weakly emarginate; mandibles reduced, not overlapping, apex acute, with stylet subequal, about 1/3 to almost 1/5 of base length; maxillary palp 4-segmented, with palpomere IV evenly acuminate or parallel-sided along apical 1/5, then abruptly convergent; labial palp 3-segmented, with apex of palpomere III deeply emarginate. **Thorax**: Pronotum with lateral expansions (frontal view) straight, bent ventrally (covering hypomeron in lateral view) to bent dorsally (Fig 8I); lateral expansion nearly as wide as the disc (Fig 8I), with posterior margin straight or rounded; posterior corner notched (Fig 8B), dorsal surface with or without rudimentary vitreous spots (Fig 8B) or well developed; posterolateral angles aligned with those of the disc (Fig 8B); prosternum with anterior margin straight or evenly emarginated throughout; proendosternite with apex entire or bifid (Fig 8E); mesoscutellum with posterior margin acute (Fig 8U) or rounded; metendosternite with anterior region of furcal arms concave or convex; sides divergent up to basal 1/4, then convergent or divergent up to basal 1/4, then convergent; Tibial spur formula: protibia 0, 1 or 2, mesotibia 2, metatibia 2; proleg, mesoleg, and metaleg without anterior claw tooth (Fig 6L–L2). **Abdomen**: terga II–V with posterior angles acute (Fig 10A); posterior margin emarginate or bisinuate (Fig 10A, B); spiracles ventral, spiracles with diameter nearly 1/5 sternite length (Fig 10A1); sternum VI with (Fig 14A1) or without lantern, lantern circular (occupying 1/3 of sternite length) (Fig 14A1); sternum VIII with posterior margin emarginate (Fig 14F); lateral margins convergent posteriorly (Fig 14F); pygidium with anterior margin medially straight, emarginate or indented (Fig 14E); posterior margin with posterolateral corners reduced or well-developed, indentation shallow; syntergite with transverse suture obliterate, membranous (Fig 14G) or connate; sternum IX with posterior margin evenly rounded (Fig 16D3), medially emarginate or truncated, posterior margin with (Fig 18D3) or without keel (Fig 14G2), posterior half entire or medially divided, forming two plates. **Aedeagus**: Phallobase with apical margin emarginate u-shaped throughout the phallobase (Fig 12C), basal margin rounded (Fig 12C) or emarginate, sagittal line either reaching the apical margin (Fig 18E1) or not (Fig 12C); phallic dorsal plate with apical 1/3 slightly curved (Fig 18E2), or strongly curved (Fig 12Q) to nearly straight (Fig 12M), apical margin rounded or acute; lateral margin smooth; basal 1/4 projected, with struts visible through phallobase (Fig 11A); paramere at least partially membranous; membranous portion nearly 1/2–1/3 or 1/4 of total length (Fig 16E3); ventral tooth on middle 1/3 present (Fig 12S) or absent, subapical ventral tooth pointed or broadened (tooth-like), apex curved ventrally, almost straight or curved outwards; basal projection present, anterior margin (dorsal view) rounded (Fig 16E), basal connection between parameres fused, V-shaped, and forming a straight line (Fig 16E) or evenly approximate towards the base.

**Distribution:** Neotropical (Fig 20).

**Remarks:** Lamprocerini was previously defined by morphological traits such as antennae, coloration, body shape, and especially the ventral position of the abdominal spiracles [see 1,67,68], but the present phylogenetic analysis called for a redefinition of this tribe. The ventral position of the abdominal spiracles (40:1) was homoplastic across Lampyrinae (e.g., *C. picipennis*, Pleotomini, and Lampirini). Lamprocerini **sensu nov.** includes the following genera: *Alecton* Laporte 1833, *Lucernuta* Laporte, 1833; *Tenaspis* LeConte, 1881; *Lychnacris* Motschulsky, 1853; *Lucio* Laporte, 1833; *Lamprocera* Laporte, 1833; and *Nyctocera* **gen. nov.**

Lamprocerini **sensu nov.** can be differentiated from other tribes of Lampyrinae by the following combination of characters: maxillary palp with palpomere IV evenly acuminate or parallel-sided up to the apical 5th, then abruptly convergent (Fig 14B); elytron with lateral expansion nearly equal in length relative to disc; terga II–V with acute posterior angles; pygidium with sides divergent posteriorly or rounded (Fig 14E), and phallic dorsal plate with acute apex (Fig 14H); apical margin rounded or acute; with lateral margin smooth; basal 1/4 projected, struts present (visible through the phallobase) (Fig 12F); paramere at least partially membranous; membranous portion extending nearly 1/2–1/3 or 1/4 of total length (Fig 18E3); ventral tooth on middle 1/3 present (Fig 12S) or absent; subapical ventral tooth pointy or broad (tooth-like), apex curved ventrally (Fig 12S), almost straight or curved outwards; basal projection present, anterior margin (dorsal view) rounded (Fig 18E), basal connection between parameres fused, basally acute and forming a straight line (Fig 18E) or evenly approximate towards the base.

**Checklist of Lamprocerini genera**

*Alecton* Laporte 1833

*Lamprocera* Laporte, 1833

*Lucernuta* Laporte, 1833

*Lucio* Laporte, 1833

*Lychnacris* Motschulsky, 1853

*Tenaspis* LeConte, 1881

*Nyctocera* Lima & Silveira **gen. nov.**

Key to Lamprocerini **sensu nov.** using males

1. Antenna with 11 or 12 antennomeres; if 11 antennomeres, antenna uniflabellate and apical antennomere bearing a subapical constriction (Nunes *et al.* [68]: 5, Fig 2); distribution restricted to Cuba *Alecton*

1’. Antenna with 11 antennomeres, shape variable; if uniflabellate, not occurring in Cuba 2

2. Antennomeres III–IX serrate (Fig 7A); palp with palpomere IV evenly acuminate (Fig 7K); prosternum with anterior margin evenly emarginate throughout; proendosternite with apex entire; sternum IX slightly asymmetric; phallobase with sagittal line not reaching apical margin 3

2’. Antennomeres III–IX cylindrical (Fig 7E); palp with palpomere IV parallel-sided up to apical 5th, then abruptly convergent (Fig 7J); prosternum with anterior margin medially straight; proendosternite with apex bifid (Fig 8E); sternum IX strongly asymmetric (Fig 10Q); phallobase with sagittal line throughout (Fig 11C) 4

3. Antennomeres III–IX lamellate (Fig 7C, D); pronotum with lateral expansions bent dorsally (Fig 8I); paramere with apex curved outwards; phallus with apex strongly curved; dorsal plate with single basal median keels *Lychnacris*

3’. Antennomeres III–IX serrate; (Fig 7A) pronotum with lateral expansions straight to bent downwards; phallus with apex slightly curved to almost straight; dorsal plate without single basal median keels 5

4. Antennomeres III–IX without double lamellae; vertex strongly depressed (Fig 7L); lateral expansion of the pronotum with posterior margin rounded; surface dorsal of the pronotum with vitreous spots rudimentary (Fig 16A); mesoscutellum with posterior margin pointed; sternum VI with lantern (Fig 16A1); sternum IX with posterior margin evenly rounded or medially emarginate (Fig 16D2) *Nyctocera* **gen. nov.**

4’. Antennomeres III–IX with double lamellae (Fig 7C); vertex slightly concave to almost flat; lateral expansion of the pronotum with posterior margin straight; dorsal surface of the pronotum without vitreous spots; mesoscutellum with posterior margin rounded; sternum VI without lantern; sternum IX with posterior margin truncated 6

5. Dorsal surface of the pronotum with vitreous spots; wing with MP3 + 4 split more basal relative to the CuA1 crossvein; proleg with one tibial spur; sternum VI with lantern; pygidium with anterior margin emarginate; pygidium with lateral margin rounded; posterior margin of the pygidium with central 1/3 rounded; pygidium with posterolateral corners well-developed; syntergite with a membranous transverse suture; phallic dorsal plate without struts; paramere with apex almost straight *Lucernuta*

5’. Dorsal surface of the pronotum without vitreous spots; wing with MP3 + 4 split more apical relative to the CuA1 crossvein; (Fig 9G); proleg with either zero or two tibial spurs; sternum VI without lantern; pygidium with anterior margin medially straight; pygidium with lateral margin divergent toward apex up to half of length; posterior margin of the pygidium with central 1/3 rounded to emarginate; pygidium with posterolateral corners reduced; syntergite with transverse suture obliterate; phallic dorsal plate with struts (visible through the phallobase); paramere apex curved ventrally *Tenaspis*

6. Mandibular stylet almost 1/5 as long as base; posterior corner of pronotum with a notch; posterior margin of the sternum VIII with a median projection *Lucio*

6’. Mandibular stylet almost 1/3 as long as base (Fig 7S); posterior corner of pronotum without a notch; posterior margin of the sternum VIII without a median projection *Lamprocera*

#### *Nyctocera* gen. nov. Lima & Silveira.

(Fig 14–19)

ZooBank LSID: urn:lsid:zoobank.org:act:16037D08-36D7-494C-A8E2-AE88C2190238

Type species: *Cratomorphus fuscipennis* Kirsch, 1865, by original designation.

Diagnosis description: Body overall yellowish (Fig 14A), brown to dark-brown (Fig 19A). **Head**: antennae filiform (Fig 14C), vertex strongly depressed (Fig 14B2), frontoclypeal suture membranous (Fig 14B2), apex of maxillary palpomere digitiform (side subparalleled, apically rounded) (Fig 14B2). **Thorax**: pronotum subtrapezoidal, 2x wider than long (Fig 18A), pair of parasagittal rudimentary vitreous spots (Fig 18A), anterior expansion medially elevated, almost longer than the disc, posterior angle with posterior corner notched (Fig 18A), hypomeron visible or covered by a lateral expansion (lateral view), proendosternite with apex bifid; with two tibial spurs on each leg (Figs 9L–L2). **Abdomen**: terga II–VII with posterior angles acute (Fig 14D), spiracles ventral (Fig 14D1); sternite VI with lanterns circular (Fig 14A1). **Aedeagus**: pygidium with indented (Fig 14E) or straight anterior margin (Fig 17D), posterior margin straight (Fig 17D); sternite VIII with posterior margin emarginate (Fig 16C); sternite IX with posterior half entirely sclerotized, posterior margin with (Fig 18D3) or without keels, rounded (Fig 16D3) or medially emarginate (Fig 18D3). **Phallus:** phallobase with apex deeply emarginate (u-shaped) (Fig 18E); apex strongly curved ventrally (Fig 18E2), dorsal plate with keel incomplete (basal); parameres with apices membranous (Fig 18E), curved ventrally (Fig 18E2); ventral tooth present. **Female:** pygidium slightly wider than long, lateral margin rounded, posterior margin straight (Fig 17D); sternum VIII as long as wide and spiculum ventrale 1/4 shorter than sternum length, with posterior margin emarginate (Fig 17E); ovipositor with baculi symmetrical and sclerotized; gonostylus minute; gonocoxite with proximal plate sclerotized, distal plate semi-membranous and bristled; proctiger plate elongate, weakly sclerotized (Fig 17F). Internal genitalia with a large and somewhat rounded spermatophore-digesting gland and two lump-like spermathecae; bursa copulatrix membranous (Figs 15G–G2).

**Etymology**: The genus name is composed of the Greek prefix *Nycto-*, which means “night” and -*cera*, meaning “horn”, or “upper part of the head”, indicating the typical antennae of nocturnal fireflies. Gender feminine.

Distribution: Western Amazonia (Northern Brazil, Colombia, Ecuador), and Chocó (Costa Rica and Panama) (Fig 20).

Remarks: *Nyctocera* **gen. nov.** is closely related to *Lamprocera* and *Lucio*, with which it shares the antenna with antennomeres III–IX cylindrical (Fig 14C); maxillary palp 4-segmented, with palpomere IV parallel-sided up to apical 5th, then abruptly convergent (Fig 14B2); prosternum with anterior margin straight (Fig 8E); proendosternite with apex bifid (Fig 8E). However, it is easily differentiated by the following combination of characters: vertex strongly depressed (Fig 14B2); distance between antennal sockets nearly 1/3–1/4 the socket width (Fig 14B2); pronotum with lateral expansion with posterior margin rounded; mesoscutellum with posterior margin pointed; dorsal surface of pronotum with rudimentary vitreous spots (Fig 14A); sternum VI with lantern (Fig 14A1); posterior margin of pygidium with central 1/3 medially straight (Fig 14E); syntergite with anterior transverse suture obliterate and dorsal plate as wide as greater width of paramere.

Checklist of ***Nyctocera* gen. nov.** species

*Nyctocera fuscipennis* (Motschulsky, 1854) **comb. nov.**

*Nyctocera sinuaticolle* (Kirsch, 1873) **comb. nov.**

*Nyctocera discorufa* (Kirsch, 1865) **comb. nov.**

*Nyctocera blattina* **sp. nov**. Lima & Silveira

Key to *Nyctocera* **gen. nov.** species based on males

1. Pygidium with anterior margin indented (Fig 14E); sternum IX with posterior margin evenly rounded and without a transverse keel (Fig 14G2); phallobase with basal margin rounded (Fig 14H) 2

1’. Pygidium with anterior margin medially straight (Fig 18B); sternum IX with posterior margin medially emarginate and with a transverse keel (Fig 18D); phallobase with basal margin emarginate (Fig 18E) 3

2. Terga VII with posterior margin emarginate (Fig 16) *Nyctocera sinuaticolle*

*2’*. Terga VII with posterior margin bisinuate (Fig 14) *Nyctocera fuscipennis*

3*.* Pronotum with lateral expansions straight (Fig 18) *Nyctocera discorufa*

3’. Pronotum with lateral expansions bent ventrally (Fig 19) *Nyctocera blattina* **sp. nov.**

#### *Nyctocera fuscipennis* (Motschulsky, 1854) comb. nov.

(Figs 14, 15)

*Cratomorphus fuscipennis* Motschulsky, 1854: 33 (description [desc.]); Gemminger, 1869: 1645 (catalog [cat.]) Olivier, 1907: 28 (systematics [syst.]); Olivier, 1910: 22 (Catalog [cat.]); Blackwelder, 1945: 356 (checklist); McDermott, 1966: 28 (Catalog [cat.]) **syn. sen. nov.**

*Cratomorphus latus* Kirsch, 1865: 72 (description [desc.]); Gemminger, 1869: 1645 (catalog [cat.]); E. Olivier, 1911: 78 (systematics [syst.]); McDermott, 1966: 28 (Catalog [cat.]) **syn. jun. nov.**

*Aspisoma yechae* McDermott, 1966: 132 (description [desc.]) **syn. jun. nov.**

**Diagnostic redescription:** Body overall brown (Figs 14, 15); antenna, fronto-clypeus, and mouthparts yellowish-brown (Fig 14A1); pronotum yellowish with brown disc (Fig 14A); scutellum yellowish-brown to brown (Fig 14A); elytra yellowish (Fig 14A); legs and mesoventer yellow to yellowish brown (Fig 14A1); abdomen yellowish, sternite VIII yellowish-brown (Fig 14F); pygidium light brown to yellowish (Fig 14E). **Male.** Pygidium (Fig 14E) wider than long, with indented anterior margin, lateral margin divergent toward apex up to half of length, posterior margin straight. Sternite VI (Fig 14D1) with luminescent organs circular (occupying 1/3 of sternite area). Sternite VIII (Fig 14F) with posterior margin emarginate, lateral margin rounded. Posterior half of sternite IX (Fig 14G2) entirely sclerotized, posterior margin without keels, rounded, strongly asymmetric, lateral processes widened toward the apex; syntergite (Fig 14G1) subtriangular, sagittal membranous suture absent, anterior transverse suture membranous. **Aedeagus:** well-sclerotized (Fig 14H–H3); phallobase (Fig 14H) with sagittal line conspicuous, apex deeply emarginate (u-shaped); parameres (Fig 14H1) symmetric, slightly smaller than phallus, with a ventral tooth on middle 1/3, apex distinctly membranous, acute and curved ventrally, basal projection with anterior margin rounded; phallus (Fig 14H2) almost 5x longer than wide, apex acute and strongly curved ventrally, dorsal plate with keel incomplete (basal 1/3), without ventral plate. **Female.** Pygidium (Fig 15D) slightly wider than long, lateral margin convex, anterior margin slightly emarginate, posterior margin flat. Sternites VI–VII (Fig 15C1) with luminescent organs (occupying 1/2 of the sternite surface). Sternite VIII (Fig 15E) wider than long, posterior margin emarginate, lateral margin rounded; spiculum ventrale short and slender, 1/4 shorter than sternite length. Ovipositor (Fig 15F–F2) with baculi symmetrical, gonostylus slender, gonocoxite with proximal plate sclerotized, distal plate semi-membranous and gently bristled; proctiger plate elongate, weakly sclerotized. Internal genitalia (Fig 15G–G2) with a large and somewhat rounded spermatophore-digesting gland and two lump-like spermathecae, bursa copulatrix membranous.

Remarks: An etymology for the specific epithet was not given by the author, however it is derived from the Latin adjective *fusci* (nominative plural of *fuscus*, meaning “dark”) and the noun *pennis* (dative plural of *penna*, meaning “wings”), probably alluding to the species’ comparatively darker elytron. This species can be recognized by the following combination characters: terga VII with posterior margin bisinuate (Fig 14D); pygidium with anterior margin indented (Fig 14E); sternum IX with posterior margin evenly rounded without keel (Fig 14G2); phallobase with basal margin rounded (Fig 14H).

Distribution: Ecuador**,** Colombia, Brazil (Acre, Amazonas, Pará) (Fig 20).

Type material: *Cratomorphus fuscipennis* Motschulsky, 1854. SYNTYPE (Fig 24C–C1) (1 ♂, pinned, ZMMU), label data: *Cratomorphus fuscipennis* Motsch, **Columb.** [yellow label, handwritten]. Syntypus, *Cratomorphus fuscipennis* Motsch [red label, handwritten]. *Cratomorphus fuscipennis* Motsch, det. Kazants: et. Nikitski [handwritten]. Boomyᴣeñ MRY (MOCKBa, POCCNR), nº ZMMU col 02771, Zool. Mus. Mosq. Univ., (Mosquae, ROSSIA), ex coll. V. I. Moltschulsky [pink label, typewritten].

*Cratomorphus latus* Kirsch, 1865. SYNTYPE (Fig 23D–D2) (1 ♂, pinned, MTKD), label data: **Bogota**, Kirsch [green label, handwritten], Staatl., Museum fűr Tierkunde Dresden [typewritten], SYNTYPUS [red label, typewritten], *Cratomorphus latus* Kirsch [handwritten], without date and collector.

*Aspisoma yechae* McDermott, 1966. HOLOTYPE (Fig 24I–I2) (1 ♀, pinned, USNM), label data: HOLOTYPE ♂ *Aspisoma yechae* McDermott [white label, typewritten]. **Ecuador**, **Santo Domingo de los Colorados**, vii.xi/1965 [handwritten]. BLNO 003688 [blue label, typewritten].

**Material examined: (**2 ♀, pinned, CNIN), label data: **Colombia**, **Calima**, Valle del Cauca, L. C. Pardo Locarno leg., 50231–50232 [typewritten]. (1 ♀, dissected and stored in microvial, CZPB), label data: **Brazil**, **Acre**, Rio Branco, Aeroporto Velho, 18–19/i/1984, P. Bührnheim col. [handwritten]. (1 ♂, dissected and stored in microvial, INPA), label data: **Brazil**, **Amazonas**, Manaus, 2F–2Km – 14, torre 40m, 2°35’21”S/60°06’55”W, 21–24.i.2004, Luz mista lençol+BLB + BL, Motta, C. S., Trovisco, S. F., Xavier, F. F. F., Filho, A. S. Col. [typewritten]. (1 ♂ pinned, INPA), label data: **Brazil**, **Amazonas**, Manaus, BR– 174 Km 50, ZF–2 Km2, 02°38’16”S/60°09’26”W, 13–27/xii/2012, F. F. Xavier F; G. Z. Lopes, A. L. Aguiar, A. L. Rodrigues, J. R. de Oliveira, Armadilha de Luz Mista [typewritten]. (1 ♂, pinned, CZPB), label data: **Brazil**, **Amazonas**, Rio Urubu, 2°10’S/59°49’W, 13–14/03/83 – P. Bührnheim, N. Otaviano e S. Leite col. [handwritten]. (1 ♀ pinned, INPA), label data: **Brazil**, **Amazonas**, Presidente Figueredo, Est. Da Balbina Km 24, Com. S. Fco. De Assis, 11.iii.2002, Pereira, E. S. Leg., Rede Entomológica, Coleoptera: Lampyridae, Pereira, E. S. Det. v.2002 [typewritten]. (1 ♂, pinned, DZUP), label data: **Brazil**, **Pará**, Jacareacanga, xii–1968, M. Alvarenga, Coleção M. Alvarenga, DZUP 091680 [typewritten].

#### *Nyctocera sinuaticolle* (Kirsch 1873) comb. nov.

(Figs 16, 17)

*Aspisoma sinuaticolle* Kirsch, 1873: 392 (description [desc.]); Olivier, 1907: 30 (systematics [syst.]); Olivier, 1910: 24 (Catalog [cat.]); Blackwelder, 1945: 357 (checklist); McDermott, 1966: 32 (Catalog [cat.]) **syn. sen. nov.**

*Cratomorphus fasciatus* Gorham, 1884: 271 (description [desc.]); Olivier, 1907: 28 (systematics [syst.]); Olivier, 1910: 28 (Catalog [cat.]); Blackwelder, 1945: 356 (checklist); McDermott, 1966: 28 (Catalog [cat.]) **syn. jun. nov.**

Diagnostic description: Body brown to dark brown (Fig 16A); antenna, fronto-clypeus, and mouthparts (Fig 15A1) yellowish-brown; pronotum (Fig 16A) with yellowish-brown disc, brown lateral margins; scutellum (Fig 16A) yellowish-brown to dark brown; elytra (Fig 16A) dark brown or brown with a transverse yellow stripe (fasciate); legs and mesoventer yellow to yellowish brown; abdomen yellowish; sternite VIII (Fig 16C) yellowish brown; pygidium (Fig 16B) light brown to yellowish. **Male.** Pygidium (Fig 16B) wider than long, with indented anterior margin, lateral margin divergent toward apex up to half of length, posterior margin straight. Sternite VI (Fig 16A1) with luminescent organs circular (1/3 as wide as sternite). Sternite VIII (Fig 16C) with posterior margin emarginate, lateral margin rounded. Posterior half of sternite IX (Fig 16D–D2) entirely sclerotized, posterior margin without keels, rounded, strongly asymmetric, lateral processes widened toward the apex; syntergite (Fig 16D) subtriangular, sagittal membranous suture absent, anterior transverse suture membranous. **Aedeagus:** well-sclerotized (Fig 16E–E3); phallobase (Fig 16E) with sagittal line conspicuous, apical margin deeply emarginate (u-shaped); parameres (Fig 16E1) symmetric, slightly smaller than phallus, with a ventral tooth on middle 1/3; apex distinctly membranous, acute and curved ventrally, basal projection with anterior margin rounded; phallus (Fig 16E2) almost 5x longer than wide, apex acute and strongly curved ventrally, dorsal plate with keel incomplete (basal 1/3), without ventral plate. **Female.** Pygidium (Fig 17D) almost 2x wider than long, lateral margin rounded, anterior margin slightly emarginate, posterior margin straight; sternites VI–VII (Fig 17C1) with luminescent organs (1/2 as wide as sternite); sternite VIII (Fig 17E) wider than long, posterior margin emarginate, lateral margin rounded; spiculum ventrale short and slender, 1/4 shorter than sternum length. Ovipositor (Fig 17F–F2) with baculi symmetrical, sclerotized; gonostylus minute; gonocoxite with proximal plate sclerotized, distal plate semi-membranous and bristled; proctiger plate elongate, weakly sclerotized.

**Remarks:** An etymology for the specific epithet was not given by the author, but “sinuaticolle” is composed of the latin adjective “*sinuatus*”, meaning “curved” or “sinuate”, and the noun “*collum*”, meaning “neck” (referring to the pronotum), likely alluding to the sinuate margins of the prothorax in this species. This species can be recognized by the following combination characters: terga VII with posterior margin emarginate; pygidium with anterior margin indented (Fig 15B); sternum IX with posterior margin evenly rounded without keel (Fig 16D3); phallobase with basal margin rounded (Fig 16E).

Distribution: Brazil (Amazonas), Ecuador and Costa Rica. (Fig 20)

Type material: *Aspisoma sinuaticolle* Kirsch, 1873. HOLOTYPE (Fig 24H–H2) (1 ♀, pinned, MTKD), label data: sinuaticolle Kir [typewritten], Holotype [red label, typewritten]; Staatl. Museum fűr Tierkunde, Dresden [typewritten], **Sarayaku**, Kirsch [typewritten].

*Cratomorphus fasciatus* Gorham, 1884. HOLOTYPE (Fig 24E–E2) (1 ♂, pinned, BMNH), label data: **Costa Rica**, Van Patten. [typewritten]. HOLOTYPE, *Cratomorphus fasciatus* Gorham, Determined by Martin, and Saxton 2020 [red label, typewritten], Type [typewritten], ♂ [handwritten], Type, Sp. figured. [typewritten], NHMUK 013584672 [typewritten], *Cratomorphus fasciatus* Gorham [handwritten], B. C. A. col. III (2) *Cratomorphus* [typewritten], fasciatus / Gorham [handwritten]. Without date and collector.

**Material examined:** (1 ♂, dissected and stored in microvial, CZPB), label data: **Brazil, Amazonas**, Coari, Ig. Marta, –34°50’073”S/65°02’37”W, 14–25/viii/1993, P. F., Bührnheim et. al col., armadilha luz mista mercúrio [typewritten]. (1 ♀, pinned, MZSP), label data: **Brazil, Amazonas**, Benjamin Constant, Rio Javari, i.1951 Dirings [typewritten], MZSP 49230 [typewritten]. (1 ♀, dissected and stored in microvial, INPA), label data: **Brazil, AM**, São Paulo de Olivença 03°28’50”S –68°55’25”W, campina, 11–14.ix.2005, Malaise, J. A. Rafael & F. F. Xavier Fº [typewritten].

#### *Nyctocera discorufa* (Kirsch, 1865) comb. nov.

(Fig 18)

*Cratomorphus discorufus* Kirsch, 1865: 72 (description [desc.]); Gemminger, 1869: 1645 (catalog [cat.]); Gorham, 1891: 48 (systematics [syst.]); Olivier, 1907: 28 (systematics [syst.]); Olivier, 1910: 22 (Catalog [cat.]); Blackwelder, 1945: 356 (checklist); McDermott, 1966: 28 (Catalog [cat.]) **syn. sen. nov.**

*Cratomorphus altivolans* Gorham, 1884: 270 (description [desc.]); Olivier, 1907: 28 (systematics [syst.]); Olivier, 1910: 22 (Catalog [cat.]); McDermott, 1966: 27 (Catalog [cat.]) **syn. jun. nov.**

**Diagnostic description:** Body overall yellowish (Fig 18A); antenna, fronto-clypeus, and mouthparts (Fig 18A1) yellowish-brown; pronotum (Fig 18A) yellowish with brown disc; scutellum (Fig 18A) yellowish-brown; elytra (Fig 18A) yellowish; legs and mesoventer yellow to yellowish brown; abdomen yellowish, sternite VIII (Fig 18A1) yellowish brown; pygidium (Fig 18B) light brown to yellowish. **Male.** Pygidium (Fig 18B) wider than long, lateral margin divergent toward apex up to half of length, posterior margin straight; sternite VI (Fig 18A) with luminescent organs circular (1/3 as wide as sternite); sternite VIII (Fig 18C) with posterior margin emarginate, lateral margin rounded; Posterior half of sternite IX (Fig 18D) entirely sclerotized, posterior margin with keels, medially emarginate, strongly asymmetric, lateral processes widened toward the apex; syntergite (Fig 18D2) subtriangular, sagittal membranous suture absent, anterior transverse suture membranous. **Aedeagus:** well-sclerotized (Fig 18E–E3); phallobase (Fig 18E) with sagittal line conspicuous, apical margin deeply emarginate (u-shaped), basal margin emarginate; parameres symmetric (Fig 18E), slightly smaller than phallus, with ventral tooth on middle 1/3, apex distinctly membranous, acute and curved ventrally, basal projection with anterior margin rounded; phallus (Fig 18E2) almost 5x longer than wide, apex acute and strongly curved ventrally, dorsal plate with keel incomplete (basal 1/3), without ventral plate. **Female:** unknown.

**Remarks:** An etymology for the specific epithet was not given by the author, but it likely stems from the Greek word “*disc*”, which means a round plate, and the Latin word “*rufa*”, which refers to the color red or brown, possibly in relation to the color of the disc on the pronotum of this species. This species can be recognized by the following combination characters: pronotum with lateral expansions straight; pygidium with anterior margin medially straight (Fig 18B); sternum IX with posterior margin medially emarginate with keel (Fig 18D3); phallobase with basal margin emarginate (Fig 18E1).

**Distribution:** Colombia, Panamá, and Costa Rica (Fig 20).

Type material: *Cratomorphus discorufus* Kirsch, 1865. SYNTYPE (Figs 24F-F2) (1 ♂, pinned, MTKD), label data: **Bogota**, Kirsch [green label, handwritten], Staatl., Museum fűr Tierkude Dresden [typewritten], SYNTYPUS [red label, typewritten], *discorufus* Kirsch [handwritten], without date and collector.

*Cratomorphus altivolans* Gorham, 1884. HOLOTYPE (Figs 24G–G2) (1 ♂, pinned, BMNH), label data: **Panama, Volcan de Chiriqui**, 2–3000 ft., Champion. [typewritten], HOLOTYPE, *Cratomorphus altivolans* Gorham, Determined by Martin, and Saxton 2020 [red label, typewritten], Type [typewritten], ♂ [handwritten], Type, Sp. figured. [typewritten], NHMUK 013584671 [typewritten], *Cratomorphus altivolans* Gorham [handwritten], B. C. A. col. III (2) *Cratomorphus* [typewritten], *altivolans* Gorham [handwritten]. Without date and collector.

Material examined: (1 ♂ dissected and stored in microvial, MPUJ), label data: **Colombia, San Cayetano**, Cundinamarca, Vda. Los rios La Quinta. El encanto 2200–2400 m.s.n.m., abril 14 de 1992. Leg. A. mera, MHN–ENT 15.006. (1 ♂ dissected and stored in microvial, MPUJ), label data: **Colombia, Jama**, Vahr B. Calima, Bajo Calima, Centro for. V.T., 70 m, 23.03.95, *Cratomorphus* sp. (Molschulsky, 1853), ident: A. Ladino P., 2019, MPUJ_ENT 0067604. (1 ♂, pinned, DZUP), label data: **Colombia**, **Guyabedal** [handwritten, back of the label: Ma 48, 12.59], DZUP 091300 [typewritten], Coleção Jorge Diniz [typewritten]. MZUF (Museo Zoologico «La Specola» dell’Università di Firenze). (1 ♂, pinned, NCSU), label data: Costa Rica, Turrialba, 16.xii.1968 [typewritten], F. Ferrer, lighttrap [typewritten], NCSU [typewritten]. (1 ♂, pinned, WCCA), label data: **Costa Rica, Turrialba**, vi.2.1986, S. Pessoa [handwritten]. (1 ♂, pinned, WCCA), label data: **Panama**, **Coclé Prov**., Cerro Gaital, 2300’, V–28–1994, F. Andrews & A. Gilbert [typewritten]. (1 ♂, pinned, WCCA), label data: Costa Rica, Cartago Province, 1.2 mi. SE Tuis, V–18/21–1992, F. Andrews & A. Gilbert [typewritten]. (1 ♂, dissected, in ethanol, WCCA), label data: **Panama, Chiriqui** Prov. Reserva Fortuna Continental Divide Trail, v.25.1993, F. Andrews & A. Gilbert [typewritten].

#### *Nyctocera blattina* sp. nov. Lima & Silveira.

(Fig 19)

**ZooBank LSID:** urn:lsid:zoobank.org:act:A3567628-E286-44C7-B9F1-4A50493FA8F4

Diagnostic description: Body dark to dark brown (Fig 19A); antenna, fronto-clypeus, and mouthparts dark to dark-brown (Fig 19A1); pronotum (Fig 19A) yellowish with brown disc; scutellum dark (Fig 19A); elytra dark (Fig 19A); legs and mesoventer dark to dark-brown; abdomen dark, sternite VIII brown (Fig 19A1); pygidium light brown (Fig 19B). **Male.** Pygidium (Fig 19B) wider than long, with anterior margin, lateral margin divergent toward apex up to half of length, posterior margin straight; sternite VI (Fig 19A1) with luminescent organs circular (1/3 as wide as sternite); sternite VIII (Fig 19C) with posterior margin emarginate, lateral margin rounded; Posterior half of sternite IX (Fig 19D–D3) entirely sclerotized, posterior margin with keels, medially emarginate, strongly asymmetric, lateral processes widened toward the apex; syntergite (Fig 19D2) subtriangular, sagittal membranous suture absent, anterior transverse suture membranous. **Aedeagus:** well-sclerotized (Fig 19E–E3); phallobase with sagittal line conspicuous (Fig 19E2), apical margin deeply emarginate (u-shaped), basal margin emarginate; parameres (Fig 19E) symmetric, slightly smaller than phallus, with ventral tooth on middle 1/3, apex distinctly membranous, acute and curved ventrally, basal projection with anterior margin rounded; phallus (Fig 19E1) nearly 5x longer than wide, apex acute and strongly curved ventrally, dorsal plate with keel incomplete (basal 1/3), without ventral plate. **Female:** unknown.

**Etymology:** The specific epithet is derived from the Latin word “*blatta*,” which means cockroach, referring to the general body shape of this order of insects.

Remarks: This species can be distinguished from others by its colour pattern (body dark to dark-brown and pronotum yellowish with brown disc) (Fig 19A), pronotum with lateral expansions bent ventrally; pygidium with anterior margin medially straight (Fig 18B); sternum IX with posterior margin medially emarginate with keel (Fig 19D–D3); phallobase with basal margin emarginate (Fig 19E1).

Distribution: Costa Rica and Panama (Fig 20).

Type material: HOLOTYPE (1 ♂, pinned, USNM), label data: **Costa Rica: Cartago**, Re. Tapantí, unnamed tribs., ca. 9 km (road) NW tunnel, 9.72 N, 83.78 W, 8–9.vi.1988, el 1400 m, C.M. & O. S. Flint, Holzenthal [typewritten]. PARATYPES: (1 ♂, pinned, USNM), label data: F. Nevermann, **Costa Rica**, Ex collzeledon [green label, typewritten], USNM [typewritten], without other data. (1 ♂, pinned, USNM), label data: **Panama: Prov. Bocas del Toro**; Cayo Nancy, 7.3 Km ESSE Bocas del Toro, 29 Feb. 1988, F. M. Greenwell [typewritten], USNM [typewritten].

**Lampyrinae Rafinesque, 1815 *incertae sedis***

#### *Bituca* gen. nov. Lima & Silveira.

(Figs 21, 22)

ZooBank LSID: urn:lsid:zoobank.org:act:84CB0BDA-3ED6-4E4E-853F-2540FE82D9E2

Type species: *Cratomorphus besckei* Olivier, 1895 **comb. nov.**, by original designation.

Diagnostic description: Body overall brown to dark-brown and yellowish; (Figs 21A; 22B) antennae filiform, head with vertex strongly depressed (Fig 21A1), frontoclypeal suture membranous, submentum as wide as long; maxillary palpomere with sides divergent, apex deeply emarginate; labrum with anterior margin round to straight or to weakly emarginate; pronotum semioval, 2x wider than long, pair of parasagittal vitreous spots (Figs 21A; 22A), lateral expansions straight, prosternum with anterior margin evenly emarginated throughout, mesoscutellum with posterior margin pointed (Fig 21B); hypomeron visible (lateral view), elytron with lateral expansion nearly half as long as disc; proendosternite with apex entire; tibial spurs absent; terga II–VII with posterior angles right-angled, spiracles ventral. **Male:** pygidium with anterior margin emarginate, posterior margin rounded, posterolateral corners well-developed (Figs 21C; 22B); sternum VII with lantern bipartite as two lateral circles (Fig 22A1) or entire (Fig 21A1); sternite VIII with posterior margin emarginate (Figs 21D; 22C); sternite IX with posterior margin evenly rounded or indented (Fig 22D–D2); aedeagus with phallobase (Figs 21E, 22E); with sagittal line not reaching apical margin, apical margin deeply emarginate (u-shaped); phallus (Figs 21E–E3; 22E–E2) with apical 1/3 almost straight, dorsal plate with keel incomplete (basal) and apical margin rounded; paramere with apex curved ventrally (Figs 21E2; 22E1). **Female:** pygidium (Fig 21G) as long as wide, with lateral margins convergent anterad, posterior margin rounded; sternum VIII (Fig 21H) as long as wide and spiculum ventrale 1/4 shorter than the sternum length, with posterior margin deeply emarginate; ovipositor (Fig 21I–I2) with baculi symmetrical, conspicuously slender and elongate, gonostylus slender; gonocoxite with proximal plate sclerotized, distal plate semi-membranous and densely bristled; proctiger membranous, weakly sclerotized medially.

Etymology: The name of the genus is a proud tribute to the Brazilian singer Milton Nascimento, who turned 80 in 2022. The artist is also affectionately known as Bituca, a nickname he received as a child for “pouting” when contradicted. Gender neutral.

Distribution: Argentina, Brazil (Pernambuco, Sergipe, Minas Gerais, Espírito Santo, Rio de Janeiro, São Paulo, Santa Catarina) and Paraguay (Fig 23).

Remarks: *Bituca* **gen. nov**. can be distinguished from genera of Lampyrinae by having a vertex strongly depressed (Figs 21A1; 22A1), submentum as wide as long, mesoscutellum with posterior margin pointed (Fig 21B), elytron with lateral expansion nearly half as long as disc (Fig 21A1), pygidium with well-developed posterolateral corners and phallus with dorsal plate apically rounded (Figs 21C; 22B).

Checklist of ***Bituca* gen. nov.** species

*Bituca besckei* (Olivier, 1895) **comb. nov.**

*Bituca miltoni* Lima & Silveira **sp. nov.**

#### *Bituca besckei* (Olivier, 1895) comb. nov.

(Fig 21)

*Cratomorphus besckei* Olivier, 1895: 146–147 (description [desc.]); Olivier, 1907: 28 (systematics [syst.]); Olivier, 1910:22 (Catalog [cat.]); Bruch, 1915: 240 (Catalog [cat.]); Blackwelder 1945: 355 (Catalog [cat.]); McDermott, 1966:27 (Catalog [cat.]); Santos *et al.* 2016: 5–6 (systematics [syst.]). **syn. sen. nov.**

*Lucernuta paraguayensis* McDermott, 1960: 82 (description [desc.]); McDermott, 1966: 26 (Catalog [cat.]) **syn. jr.**

Diagnostic redescription: Male: labrum with anterior margin straight to weakly emarginate; sternites VI and VII (bearing lanterns) longer than the preceding sternites (Fig 21A1); sternum VII with lantern entire (Fig 21A1); pygidium (Fig 21C) with anterior margin emarginate, posterior margin rounded; sternite VIII (Fig 21D) with posterior margin emarginate; sternite IX with posterior margin indented; aedeagus (Fig 21E–E3) with phallobase bearing a sagittal line not reaching the apical margin, apical margin deeply emarginate (u-shaped); phallus with apex 1/3 almost straight, dorsal plate with keel incomplete (basal); paramere with apex curved ventrally. **Female:** pygidium (Fig 21G) as long as wide, with lateral margins convergent anterad, posterior margin rounded; sternum VIII (Fig 21H) as long as wide and spiculum ventrale 1/4 shorter than the sternum length, with posterior margin deeply emarginate; ovipositor (Fig 21I–I2) with baculi symmetrical, conspicuously slender and elongate, gonostylus slender; gonocoxite with proximal plate sclerotized, distal plate semi-membranous and densely bristled; proctiger membranous, weakly sclerotized medially.

**Remarks**: According to Papavero [69], two individuals named “Bescke” collected in South America – the father Christian Friedrich Carl Bescke and his son, Carl Heinrich Bescke. The first traveled in 1821 to the cities of Buenos Aires, Rio de Janeiro in Guanabara (sic; corresponds to modern day Rio de Janeiro state), and Salvador in Bahia, and the second settled in the State of Rio de Janeiro between 1831 and 1851, especially in Nova Friburgo. The available information does not clarify which of the two sent material to E. Olivier (who refers to one of them as a traveler), and the reference to “Rio de Janeiro” leaves uncertainty as to whether the type locality corresponds to the city of Rio de Janeiro or Nova Friburgo.

This species was recently redescribed in detail [see 12]. Although externally similar to some *Cratomorphus* spp., *B. besckei* **comb. nov.** can be distinguished by having sternum VII with lantern entire; phallobase with sagittal line not reaching the apical margin, apical margin deeply emarginate (u-shaped); dorsal plate of the phallus with keel incomplete and basal, and paramere with apex curved ventrally. In addition, *B. besckei* **comb. nov.** is also behaviorally very distinctive due to its yellow flashes in trains of five pulses, while *Cratomorphus* males often produce long glows during flight [12].

**Distribution:** Argentina, Paraguay, and Brazil (Minas Gerais, Espírito Santo, Rio de Janeiro, São Paulo, Santa Catarina) (Fig 23).

Type material: *Cratomorphus besckei* Olivier (1895). SYNTYPE (Fig 24K–K2) (1 ♂, pinned, MNHN), label data: **Rio de Janeiro** [handwritten], ♂ [handwritten], Without date and collector.

*Lucernuta paraguayensis* McDermott. HOLOTYPE (Fig 24J–J2) (1 ♂, pinned, USNM 66698), label data: **Paraguay**: Itapúa, Hohenau, XII/1959, 1 Male, w/o coll. (USNM 66698).

**Material examined: Brazil: Espírito Santo**: Santa Teresa, 1 male, 25.i.2014, no collector’s name, active search (DZRJ); **Minas Gerais:** Alto Caparaó, Parque Nacional do Caparaó, Trilha entre Tronqueira e o terreiro, 1 male, 19.i.2014, A. Santos *et al*., active search (DZRJ); **Rio de Janeiro:** Guapimirim, Parque Nacional Serra dos Órgãos, 2 males, 15–18.ii. 2015, L. Silveira, active search (DZRJ). **Itatiaia,** Penedo, Três Bacias, Rio das Pedras, 1 male, 06.iii.2008, J. Nessimian, Light (DZRJ). **Rio de Janeiro,** Camorim, Parque Estadual da Pedra Branca, Trilha do açude, 1 male, 04.ix.2017, A. Ferreira, active search (DZRJ), 1 male, 12.iv.2017, L. Silveira, S. Vaz, A. Ferreira, L. Campello, active search (DZRJ). **Rio de Janeiro,** Taquara, Parque Estadual da Pedra Branca, Cachoeira do Sininho, 1 male, 06.iv.2017, A. Ferreira, active search (DZRJ). **Teresópolis**, Parque Nacional da Serra dos Órgãos, Trilha Suspensa, 1 female, 12.i. 2015, S. Vaz, active search (DZRJ). **Rio de Janeiro,** Angra dos Reis – Jussaral, Trav. & Almeida, 8–4–1935 (CEIOC– 68926); **São Paulo**: Ilha Bela, Ilha de Búzios, 1 male 16.X–04.XI.1963, Expedição Departamento Zoologia (MZUSP), Salesópolis, Estação Biológica de Boracéia, 1 male, 23–28.I.2002, S. A. Casari & G. I. M. Santos (MZUSP). **São Paulo:** Guapiara, Pq. Intervales, 13.i.2019, Viviani, V., (UFSCar–1704); **Santa Catarina**: Seara (=Nova Teutônia), 4 males, 300–500m, 21.xii.1948, xi.1946 and xii.1950, F. Plaumann col. (MZUSP); Paraguay: Hohenau: xii.1957, no collector’s name (SMNH); **Argentina**: Misiones (MNHN).

#### *Bituca miltoni* Lima & Silveira sp. nov.

(Fig 22)

ZooBank LSID: urn:lsid:zoobank.org:act:A091D0D8-B4C4-4165-B884-A19E403ED677

Diagnostic description: Male: pygidium (Fig 22B) with anterior margin emarginate, posterior margin rounded, sternite VIII (Fig 22C) with posterior margin emarginate; sternite IX (Fig 22D–D2) with posterior margin evenly rounded; aedeagus (Fig 22E–E2) with phallobase bearing a sagittal line not reaching the apical margin, apical margin deeply emarginate (u-shaped); phallus with apical 1/3 nearly straight, dorsal plate with keel incomplete (basal); paramere with apex curved ventrally. **Female:** unknown

**Etymology:** The specific epithet honors the Brazilian singer, composer, and multi-instrumentalist Milton Nascimento, recognized worldwide as one of the most influential and talented artists of Brazilian Popular Music.

Remarks: The new species can be distinguished from *Bituca besckei* **comb. nov.** by the following combination of characters: labrum with anterior margin round (straight to weakly emarginate in *B. besckei* **comb. nov.**) (Fig 22A1); sternum VII with lantern bipartite as two lateral circles (entire in *B. besckei* **comb. nov.**) (Fig 22A1) and sternum IX with posterior margin evenly rounded (indented in *B. besckei* **comb. nov.**).

Distribution: Brazil (Pernambuco and Sergipe) (Fig 23).

Type material: HOLOTYPE (1 ♂, dissected and stored in microvial, MZSP), label data: **Brazil, Pernambuco**, Igarassu Refúgio Charles Darwin, 08.III.1997, R. C. Moura col., MZSP 49242. PARATYPE (1♂, pinned, INPA), label data: **Brazil, Sergipe**, 2014–08. 19.vi.2014, Pensilvânia, LMT, APMS, ACD, WRMS.

## 4. Discussion

Our study used a thorough phylogenetic analysis of 97 morphological characters, based on the most comprehensive sampling of Cratomorphini taxa to date (N = 35). In addition to the targeted ingroup, we included representatives from all other tribes of Lampyrinae to test Cratomorphini’s monophyly. Our results corroborate previous findings challenging the monophyly of Cratomorphini based on genetic [2] and morphological [24,27] evidence – despite differences in taxon sampling among these studies.

We recovered *Aspisoma* sister to (*Pyractomena*, *Aspisomoides*), but distantly related to *Cratomorphus* **sensu nov.** (Fig 2). This topology is consistent with those of Martin *et al.* [2,25], which recovered (*Micronaspis* (*Pyractomena*, *Aspisoma* sp1) – with *Aspisoma* sp2 instead sister to *Lamprocera* [25] and *Pyractomena* sister *Aspisoma* [2]. We suspect that *Aspisoma* sp2 in Martin *et al.* [25] actually corresponds to *Nyctocera* **gen. nov.**, with which it can be easily confused based on external traits (consider, for example, that *Aspisoma yechae* [Fig 24I–I2] is a synonym of *Nyctocera fuscipennis* **comb. nov.** [Fig 24C–C1]). The absence of *Cratomorphus* in Martin *et al.* [25], and the inclusion of only an unidentified *Cratomorphus* species in Martin *et al.* [2], further limits detailed comparisons. One important topological difference between our study and Jeng’s [24] was that *Cratomorphus* and *Aspisoma* were found nested in the Lamprocerini, in a polytomy with *Alecton* (not included here), while *Pyractomena* clustered with a Lucidotini group that included *Pyractonema*, *Pyropyga*, *Robopus*, and *Photinus* (also represented then by the synonyms *Macrolampis* and *Ellychnia*).

Our findings support an updated diagnosis of deep Lampyrinae lineages previously inferred only from molecular data. We recovered a clade comprising *Bituca*, Cratomorphini **sensu nov.**, Lamprocerini, Lampyrini, and *Pleotomus* (Fig 2), congruent with the sister group of Aspisomini + Lucidotini in Martin *et al.* [2]. Building up from Jeng’s findings [24], we identified five synapomorphies supporting this clade: (i) apex of the paramere with a pointy, membranous tip (char. 88: 0); (ii) dorsal plate narrower than paramere (char. 72: 1); (iii) sternum IX medially split in two halves (char. 63: 1); (iv) spiracles ventrally placed (reversed in *Cratomorphus* **sensu nov.** (char 41: 0); (v) radial cell segment between r3 and r4 at least 1/5 wider than r4 length (char. 34:1). Observing many of these traits requires dissection, which stresses the importance of thorough anatomical investigation for understanding lampyrid evolution and refining their classification.

The phylogenetic evidence presented here has important implications for the classification and trait evolution of Cratomorphini and Lampyrinae at large. Several taxa previously classified as Cratomorphini were found distantly related within Lampyrinae, rather than being sister to or even closely related to *Cratomorphus*, as previously suggested. This result called for a re-evaluation of the Lampyrinae tribes Cratomorphini and Lamprocerini, and supports the proposal of a new tribe, Aspisomini **trib. nov.**.

Although relationships among *Bituca* **gen. nov.**, Cratomorphini **sensu nov.**, and parts of Lampyrini and Pleotomini remain unresolved within the basal polytomy recovered in our analyses, the taxonomic changes proposed herein are based on the recognition of monophyletic, morphologically diagnosable, and internally cohesive lineages. This lack of resolution invites further scrutiny of these relationships. The proposal of the *Bituca* improves lampyrine taxonomy by refining the boundaries of *Cratomorphus*, therefore ameliorating the diagnosability of these overall similar lampyrine taxa. Retaining the previous broader circumscriptions would obscure the morphological and evolutionary diversity recovered in our analyses.

### 4.1 Systematics of Cratomorphini and Cratomorphus

Our analyses consistently recovered Cratomorphini *sensu* McDermott [1] as polyphyletic (Fig 2), as recovered in [2] with phylogenomic data. Four distinct clades heretofore classified as Cratomorphini were recovered scattered across the Lampyrinae: (i) *Cratomorphus* (partim: the type species, *C. splendidus*, and associated species) + *Erythrolychnia*; (ii) *Cratomorphus* (partim; transferred here to ***Nyctocera*** **gen. nov.** [Lamprocerini]); (iii) *Cratomorphus* (partim; transferred here to ***Bituca*** **gen. nov.** [Lampyrinae *incertae sedis*]) and (iv) Aspisomini trib. nov.: (*Micronaspis* ((*Aspisomoides, Pyractomena*) *Aspisoma*)).

The polyphyly of Cratomorphini and of *Cratomorphus sensu* McDermott [1] stems from overreliance on a few external features – especially those of the pronotum (i.e., anteriorly round, bearing vitreous spots on dorsal surface), mouthparts (i.e., mandibles slender and conspicuously exposed above), and abdomen (i.e., abdominal spiracles dorsal and laterally projected tergites) – for classification [11,70]. For instance, Cratomorphini *sensu* McDermott [1] has been distinguished from Lamprocerini by the position of the spiracles, which are dorsal in Cratomorphini and ventral in Lamprocerini. However, our results indicate that ventral spiracles are likely plesiomorphic in this broader Lampyini+Cratomorphini+Lamprocerini lineage, as seen in the ventral spiracles of *C. picipennis,* and were subsequently reversed to a dorsal position in *Cratomorphus* **sensu nov.** + *Erythrolychnia* (which represents the ancestral condition for all lampyrids).

Historically, the diagnosis of *Cratomorphus* did not include genitalic features, which are known to yield high diagnostic value [12,68,71]. Such traditional external characters were recovered here as homoplastic, reflecting repeated convergence among distantly nocturnal lineages (e.g., *Aspisoma* spp. and *Cratomorphus* spp.) subject to similar selective pressures organs involved with sexual signaling (e.g., head sensors and lanterns – see 72). A similar pattern of convergence has also been reported in other phylogenetically distant taxa, such as Lampirini and Lucidotini [25,27,28,72–74]. Although these labile and rapidly evolving characters are often recovered homoplastic at deeper phylogenetic levels across different phylogenies, they are however useful for identification and can contribute to improved resolution in combined morphological-molecular analysis, especially at shallower nodes.

The position of *Erythrolychnia bipartita* was unstable in our results. We argue that its nested placement within *Cratomorphus* **sensu nov.** (supported byBI and MPIW–K1) is most plausible, contrasting with its recovery as a sister group under higher values of K (>3) and MPEW. These conflicting topologies are mostly driven by how much relative weight is placed on traits of the sensory organs (*viz.* eyes, antennae), spiracles, and genitalia. When *E. bipartita* is recovered nested in *Cratomorphus* **sensu nov.** (MPIW–K1), larger eyes with a deep vertex, filiform antennae, and a sternum VII bearing a lantern are considered ancestral, then reversed and convergent, in *E. bipartita*. In contrast, when *E. bipartita* is sister to *Cratomorphus* **sensu nov**. (K > 3) and MPEW, the shallow head vertex, serrate antennae, and lack of lanterns of the former is reconstructed as plesiomorphic in the context of Lampyrinae. Our study suggests that *E. bipartita* could be a highly modified *Cratomorphus* **sensu nov.**, but future studies with a broader sampling of *Erythrolychnia* – including the type species, which could not be included here – are needed to flesh out whether the condition of sensory organs in *Erythrolychnia* represent ancestral or convergent features in Cratomorphini.

Among the homoplasies that support the status of Cratomorphini **sensu nov.**, the dorsal plate of the phallus with paired longitudinal keels (char. 77:1) (Fig 12I) and the dorsal plate of the phallus with a translucent window (char. 82:1) (Fig 13D) stand out. The phallus with paired longitudinal keels is interpreted here as having evolved convergently between *Cratomorphus* **sensu nov.** and the sister group to *Micronaspis*, i.e., the clade including the remaining Aspisomini **trib. nov.** (Fig 2). Likewise, the phallus with a translucent window is interpreted as having evolved convergently between *Cratomorphus* **sensu nov.** and an inner group of *Aspisoma* species (*A. physonotum*, *A. buyssoni*, *A. laetum*, *A. lineatum*, *A. sticticum*, and *A. maculatum*). We presume that the emergence of paired latitudinal keels on the dorsal plate of the phallus may act as a stabilizing mechanism during copulation dynamics. We hypothesize that these keels potentially help anchor and/or facilitate the sliding movement of the phallus within the female. Furthermore, the presence of a translucent window (char. 82:1) on the dorsal plate of the phallus could represent a structurally flexible region which aids movement of the phallus during copulation. The homoplastic status of these characters is surprising and deserves further examination.

*Cratomorphus* **sensu nov.** is supported by one non-homoplastic synapomorphy: the incomplete, transverse sternum VI lantern (char. 44:2). The biological meaning of this character is unknown, but the presence of large lanterns that cover the entire sternite is associated with species that produce flashes, while smaller lanterns are associated with glows [27; our pers. Obs.]. Likewise, both *C. cossyphinus* and *C. splendidus* have lanterns that do not occupy the entire surface of the sternite and have a bioluminescence pattern characterized by a continuous glow [2; Silveira, pers. obs.]. Therefore, other *Cratomorphus* species with comparatively smaller lanterns may emit glows as well. All other traits supporting *Cratomorphus* **sensu nov.** are homoplastic, mostly stemming from the abdomen – particularly terminalia and genitalia – but also from other body parts, such as the depressed head vertex (char. 5:1), and the presence of a tooth in the anterior claws of proleg (char. 36:1), mesoleg (char. 37:1), and metaleg (char. 37:1) (Fig 2). The toothed claws may not be related to copulation, as they are observed in both sexes. Instead, these teeth may increase grip (e.g., *Scissicauda disjuncta*, [see 29]), particularly for maintaining stability on rough surfaces of underwater vegetation, where females typically lay their eggs.

A moderately supported clade within *Cratomorphus* **sensu nov.** includes *C. bifenestratus*, *C. cossyphinus*, *C. signativentris, C. dorsalis*, *C. splendidus, C. diaphanus,* and *C. albomarginatus* – and often includes *E. bipartita* (see above)*.* When *E. bipartita* is included, this group is supported by two non-homoplastic synapomorphies: a basally depressed dorsal plate of phallus (char. 76:1), and a truncate basal projection of the parameres (char. 94:0). Among the *Cratomorphus* groups proposed by Olivier, 1895, only Group I – represented here by the clade (*C. splendidus + C. diaphanus* + *C. albomarginatus*) – was recovered as monophyletic (MP–EW, MP–IW, BI) (Figs 2, 3). This clade was supported by two non-homoplastic synapomorphies: projection of the posterior margin of the sternum VIII reaching almost half the length of sternum VIII (char. 49:2) and offset (char. 50:1). The specialized male-specific sternite VIII projection may enhance stability during copulation, thus avoiding being dislodged by competitors, as found elsewhere among fireflies (see, e.g., [75]). These two non-homoplastic synapomorphies are in fact the same taxonomic characters that Olivier [31] used to delimit group I in his classification. The identity of Olivier’s groups II, III, and IV must be clarified on an analysis of a broader taxonomic coverage of *Cratomorphus*.

The redefinition of *Cratomorphus* **sensu nov.** involved transferring some species to *Nyctocera* **gen. nov.** and *Bituca* **gen. nov.** (Fig 2). These were heretofore considered members of *Cratomorphus* because they present some former diagnostic traits of this genus, such as: concave head apex, well-developed eyes, and pronotal translucent spots – all of which convergent. These two new genera have abdominal spiracles in a ventral position, a trait also seen in other tribes within Lampyrinae, such as Lamprocerini and Pleotomini, which raised questions about their placement in Cratomorphini in previous analyses [1,12]. Jeng [24] showed an overlap in taxonomic boundaries between Lamprocerini and Cratomorphini, showing that Lamprocerini can only be considered monophyletic after the inclusion of Cratomorphini. Our findings (Fig 2) supported a redefinition of these two tribes (see above).

### 4.2 Terminalia and genitalia provide a wealth of relevant traits of congruence and stability for lampyrine systematics

Terminalic and genitalic morphology are robust sources of phylogenetic information that have repeatedly contributed to the resolution of boundaries across the Lampyridae [7,12,29,75]. Our results show that this signal is informative across node depths, with traits of various levels of homoplasy, from moderate to non-homoplastic (S3, chars. 58–96 average RI min: 0.5, max: 1; mode of L min: 0, L max: 6). These traits are on average more stable than both Signaling and Somatic traits (see Results above), and add a wealth of informative traits to the phylogenetic reconstruction of Cratomorphini taxa, and for the lampyrine at large. Although terminalia and genitalia characters have long been recognized as more informative of lampyrid relationships than those involved in signaling [7,12,29,75], our work is the first to statistically test and confirm the significance of differences in homoplasy levels between these trait groups.

Despite the systematic relevance of these traits, our dataset also highlights substantial gaps in the availability of genitalic data across Lampyridae. Many Cratomorphini taxa are still known only from external morphology (e.g., *Cassidomorphus*) or their terminalia and genitalia are insufficiently described and/or illustrated (e.g., *Paracratomorphus*), and male and female genitalia remain undocumented for a large proportion of lineages. Yet, the functional morphology of these traits in copula are completely unstudied. These limitations affect our ability to fully assess whether observed patterns reflect functional convergence, shared ancestry, or a combination of both. Adding female data may contribute to the resolution to pervasive polytomies such as at the base of Lampyrini, Cratomorphini, Pleotomini, Lamprocerini, and *Bituca.*

### 4.3 Lamprocerini taxonomy revisited

Our study was primarily targeted at Cratomorphini *sensu* McDermott [10], but the phylogenetic position of several species formerly placed in *Cratomorphus* led us to consider the phylogeny of the Lamprocerini as well. Our study covered five of six Lamprocerini genera *sensu* McDermott [10], and recovered Lamprocerini **sensu nov.** as monophyletic upon the addition of *Nyctocera* **gen. nov.** species, three of which formerly assigned to *Cratomorphus*. Males in this new genus stand out from the other Lamprocerini for having reduced, simpler antennae and well-developed eyes, unlike those seen for example in the closely related *Lucio* and *Lamprocera*. That male head shape is suggestive of a nocturnal lifestyle that is more reliant on light signals [76], similar to those of other confirmed “glower” taxa, such as *Lampyris* or *Lamprohiza*. Given (i) the lack of adult lanterns (at least those on sterna VI and VII which are typical of flashers and glowers) on the more basal Lamprocerini *Tenaspis*, *Lychnacris*; (ii) the diurnal habits of *Lucernuta savignii* (despite the functional adult lanterns; see [12]); and (iii) the elaborate biflabellate antennae of *Lucio* and *Lamprocera*, and diurnal habits (at least in *Lucio pictum* [77] and *Lamprocera latreillei* [L. Silveira pers. ob.], it is very likely that *Nyctocera* **gen. nov.** represents an evolutionary transition to a nocturnal lifestyle and a light-based sexual communication rather than pheromones.

Three non-homoplastic synapomorphies support *Nyctocera* **gen. nov.** as sister to (*Lucio*, *Lamprocera*): the broad apical maxillary palpomere (char. 12:1), bifid tips of the proendosternites’ arms (char. 25:1), and the strongly asymmetrical sternum IX (char. 65:1). The latter is interpreted as potentially enhancing the grip mechanism during copulation, stabilizing sperm transfer while simultaneously supporting the apex of sternum IX in the ovipositor [75,78]. Two other non-homoplastic synapomorphies support this clade as sister to *Lychnacris*: dorsal plate of phallus strongly sinuose (char. 70:1) and bearing a basal median keel (char. 80:1), which we suggest may provide greater flexibility and more precise maneuvering of the phallus within the female reproductive tract, and structural reinforcement, respectively. One non-homoplastic synapomorphy supports this clade as sister to *Tenaspis*: the pygidium with sides divergent posteriorly. The function of this trait is unknown, but we speculate it would offer a broader attachment to the female abdomen, thus improving grip during copula (Fig 2).

Within *Nyctocera* **gen. nov.**, *N. blattina* **sp. nov**. and *N. discorufa* **comb. nov.** share a non-homoplastic synapomorphy: the presence of a transverse keel on the posterior margin of sternite IX (char. 62:1). One of the putative functions of this trait is to act as a locking device during copulation or provide structural stiffness during sperm transfer [see 76]. Distinct terminalia structures, such as those suggested to play a role in enhancing male grip, are generally species-specific. Copulatory clamps are found in Luciolinae (e.g., *Pteroptyx* [see 78]) and Lampyrinae: Lucidotini (e.g., *Scissicauda* [see 29]; *Haplocauda* [see 30], and *Luciuranus* [see 76]). Furthermore, the bulging on the posterior margin of sternum IX may enhance or maintain alignment and physical connection between males and females, preventing disengagement or misalignment. This clade forms a polytomy with the two other *Nyctocera* species, *N. sinuaticolle* and *N. fuscipennis* (Fig 2).

The monophyly of *Bituca* **gen. nov.** was also well-supported, but its affinities remain elusive (Figs 2, 3). Given the lack of well-supported close phylogenetic affinities with *Cratomorphus* **sensu nov.** and also with any other tribe in our analysis, we have transferred it to Lampyrinae *incertae sedis*, pending further molecular investigation. The lack of distinctive genitalic features in the context of lampyrine suggest a more basal placement, as often recovered in Maximum Parsimony reconstructions (Fig. 3B).

The placement of the monotypic genera *Cassidomorphus* and *Paracratomorphus* in Cratomorphini is tentative and warrants reevaluation in forthcoming phylogenetic studies. *Cassidomorphus* is only known from a female holotype (Fig 24A–A1), and *Paracratomorphus* is known only from the male holotype (Fig 24B–B2), and collecting more specimens is an important step to infer their phylogenetic relationships.

### 4.4 Aspisomini trib. nov. and its evolutionary relationships

Since *Aspisoma*, *Aspisomoides*, *Micronaspis*, and *Pyractomena* were recovered as a distinct clade distantly related to Cratomorphini **sensu nov.** (i.e., not forming a monophylum), we proposed a new tribe, Aspisomini **trib. nov.** (Fig 2). These findings are also in agreement with previous studies [2], which found *Aspisoma* and *Pyractomena* in a clade distant from *Cratomorphus*. Two key traits shared between Aspisomini **trib. nov.** and Cratomorphini **sensu nov.** are the reduced mandibles with a distinct stylet, and the dorsal placement of the spiracles. Our analyses reconstruct the reduced mandibles of Aspisomini **trib. nov.** as synapomorphic of Aspisomini **trib. nov.** and Cratomorphini **sensu nov.**, in contrast to analyses that found Aspisomini **trib. nov.** sister to Lucidotini [2]. In the latter topology, the considering reduced mandibles as plesiomorphic for Lampyrinae (with a reversal in Lucidotini) or convergent in Aspisomaini and in the broad node that includes Cratormophini, Lamprocerini, and Lampyrini, would be equally parsimonious. Reduced, but differently shaped mandibles are otherwise found in the Amydetinae *Photoctus* McDermott, 1961 [79], which shows that a convergent reduction of mandibles is plausible. The dorsal placement of the spiracles is regarded here as a reversal in Cratomorphini **sensu nov.** from the plesiomorphic ventral state of the node that includes Cratormophini, Lamprocerini, and Lampyrini. The plesiomorphic dorsal spiracles are still seen in Aspisomini **trib. nov.** and Lucidotini.

Two important non-homoplastic synapomorphic traits supporting Aspisomini **trib. nov.** stem from the metendosternite (chars. 28:1 and 29:1), a structure rarely considered in phylogenetic studies of the Lampyridae. The biological meaning of these characters is still uncertain, but may relate to flight muscle attachment and hence improve stability during flight [80,81]. In addition, two homoplastic synapomorphies support this clade: the emarginate basal margin of the phallobase (char. 68:1) and the flat base of the phallic dorsal plate (char. 68:1), and may reflect changes in aedeagal movement and/ or fit during mating. Another remarkable shared trait of Aspisomini **trib. nov.** is the aquatic or semi-aquatic larval habitats, otherwise uncommon in New World lampyrids (a notable exception being the bromeliad-dwelling *Psilocladus costae* larva; [82]). *Micronaspis* monophyly had been supported before based on larval characters, while adult characters supporting this genus were unknown [5]. Our study found one non-homoplastic synapomorphy based on adult morphology supporting *Micronaspis* monophyly: the strongly emarginate anterior margin of the prosternum (char. 24:2). In our analysis, *Micronaspis* emerged as a sister to the other Aspisomini **trib. nov.** genera [25,28].

The clade comprising (*Aspisomoides* (*Pyractomena* + *Aspisoma*)), which is recovered as sister to *Micronaspis* within Aspisomini **trib. nov.** shares two non-homoplastic synapomorphies previously undocumented in the literature: the obliteration of the frontoclypeo-labral suture (char. 7:1) and the acute dorso-basal projection where the parameres meet (char. 89:1).

*Aspisomoides* was included here for the first time in phylogenetic studies of Lampyridae, and was found sister to *Pyractomena* (Fig 2). This clade is supported by two non-homoplastic synapomorphies: an anteriorly indented labrum (char. 8:1), and sides of phallobase apically indented (char. 66:1). In previous phylogenetic studies, *Pyractomena* was recovered sister to *Aspisoma* [2,5,26,27] or in Lucidotini [24]. *Pyractomena* is supported by two non-homoplastic synapomorphies described for the first time, as follows: the dorsal plate of the aedeagus with apex emarginate (char. 71:4) and the presence of a ventral plate in the aedeagus (char. 85:1) – a character very rare in Lampyrinae outside of Lucidotini (none of which sampled here [83,84]). Among the diagnostic characteristics of *Aspisomoides* highlighted by Zaragoza-Caballero [18], the presence of longitudinal elytral costae (char. 30) and parameres with the presence of small subapical hooks (char. 90:1) stand out. While the elytral costae was recovered as a non-homoplastic synapomorphy of this genus, the parameral subapical ventral tooth is shared with *Pyractomena*, and it is convergent with *Cratomorphus distinctus.* Similar hook-like structures on the apical or subapical portions of the parameres have also been reported in closely related families, such as Elateridae [85], suggesting that these structures may be more widespread within Elateroidea than currently recognized.

This is the first phylogenetic study to infer the relationships among *Aspisoma* species. One interesting non-homoplastic synapomorphy of *Aspisoma* is the grooved inner surface of the parameres. The relationships within *Aspisoma* were overall labile and poorly supported, except for a group including (*A. ignitum* (*A. buyssoni*, *A. physonotum*, *A. laetum*, *A. lineatum*, *A. stictium*, *A. maculatum*)), which was supported by several homoplastic synapomorphies (Figs 2, 3). This group is particularly interesting because it includes the type species of this genus. The clade consisting of *A. physonotum*, *A. buyssoni*, *A. laetum*, *A. lineatum*, *A. sticticum*, and *A. maculatum* was also well supported, but poorly resolved. This clade is supported by a non-homoplastic synapomorphy: a pronotum with posterolateral angles projected backwards (more posterior to the posterior angles of the disc). It is plausible that the posterolateral angles of the pronotum play a role in the turtle-like defensive stance typical of the *Aspisoma*, which is similar to that of the tortoise beetles (Chrysomelidae: Cassidinae), bringing it closer to the substrate and shielding the head [see [86]].

## 5. Conclusions

Cratomorphini *sensu* McDermott [1] was consistently found polyphyletic, with species split in three lineages – one clustered with the type species, one deeply nested in Lamprocerini, and one of more uncertain affinities but often at a more basal position in a clade that included Lampyrini, Cratomorphini, and Lamprocerini. These results supported updated definitions of Lamprocerini and Cratomorphini, the description of two new genera and a new tribe, and several new combinations. Our study confirmed that traits involved in sexual signaling such as eye size, pronotal vitreous spots, and lantern presence and development are extensively homoplastic in Lampyrinae, as demonstrated by their low retention indices. While these labile traits are poor indicators of deep phylogenetic relationships, they remain informative at the species level when analyzed in combination. In contrast, our novel terminalic and genitalic characters and states are substantially more stable, providing synapomorphies that define the tribal backbone of Cratomorphini **sensu nov**. and Aspisomini **trib. nov..** By incorporating this broader set of morphological data, including both key traits of the terminalia and novel internal characters (e.g., endosternites), we provide more robust diagnoses and improve the morphological delimitation of species, enabling more accurate taxonomic identifications in the future. These results emphasize the need for a deeper evaluation of both functional and structural traits showing that integrating stable genitalic transitions with plastic signaling traits is essential to resolve the complex interplay between morphology, sexual selection, and lineage diversification in Lampyrinae.

## Supporting information

S1 File A simplified morphological matrix in nexus format with 97 morphological characters used in the cladistic analysis. (NEX)

S2 File Implied-weighting parsimony analyses were conducted using unevenly spaced K values (K  =  1, 3, 5, 10, 20) and symmetric resampling with 1,000 replicates. A: Maximum parsimony under implied weighting with K = 1; B: Maximum parsimony under implied weighting with K = 3; C: Maximum parsimony under implied weighting with K = 5; D: Maximum parsimony under implied weighting with K = 10; E: Maximum parsimony under implied weighting with K = 20.
(PDF)

S3 File Character Retention Indices (RI), and extra steps (L) summary statistics calculated from 120 equally most parsimonious trees (MPTs) under equal-weights maximum parsimony (MP-EW). Acronym: SD = Standard deviation.
(XLSX)

S4 File Model selection used as input for Bayesian inference based on an unpartitioned scheme, retrieved from IQ-TREE. (IQTREE)

S5 File Partition scheme and model selection obtained from IQ-TREE based on homoplasy-level partitioning. (ZIP)

S6 File Results of the steppingstone sampling analysis used for Bayes Factor comparison of the two partitioning schemes (unpartitioned vs. homoplasy‑partitioned). (ZIP)

S7 File Compressed file with the MrBayes input script (in nexus format) and all output files from the non-partitioned analysis. (ZIP)
