# Supplementary figures and images for: Systematics of giant neotropical fireflies and their kin (Lampyridae: Lampyrinae)
Source: PLoS One. 2026 Aug 3;21(8):e0354465. doi: 10.1371/journal.pone.0354465 (PMC13432145; doi:10.1371/journal.pone.0354465)

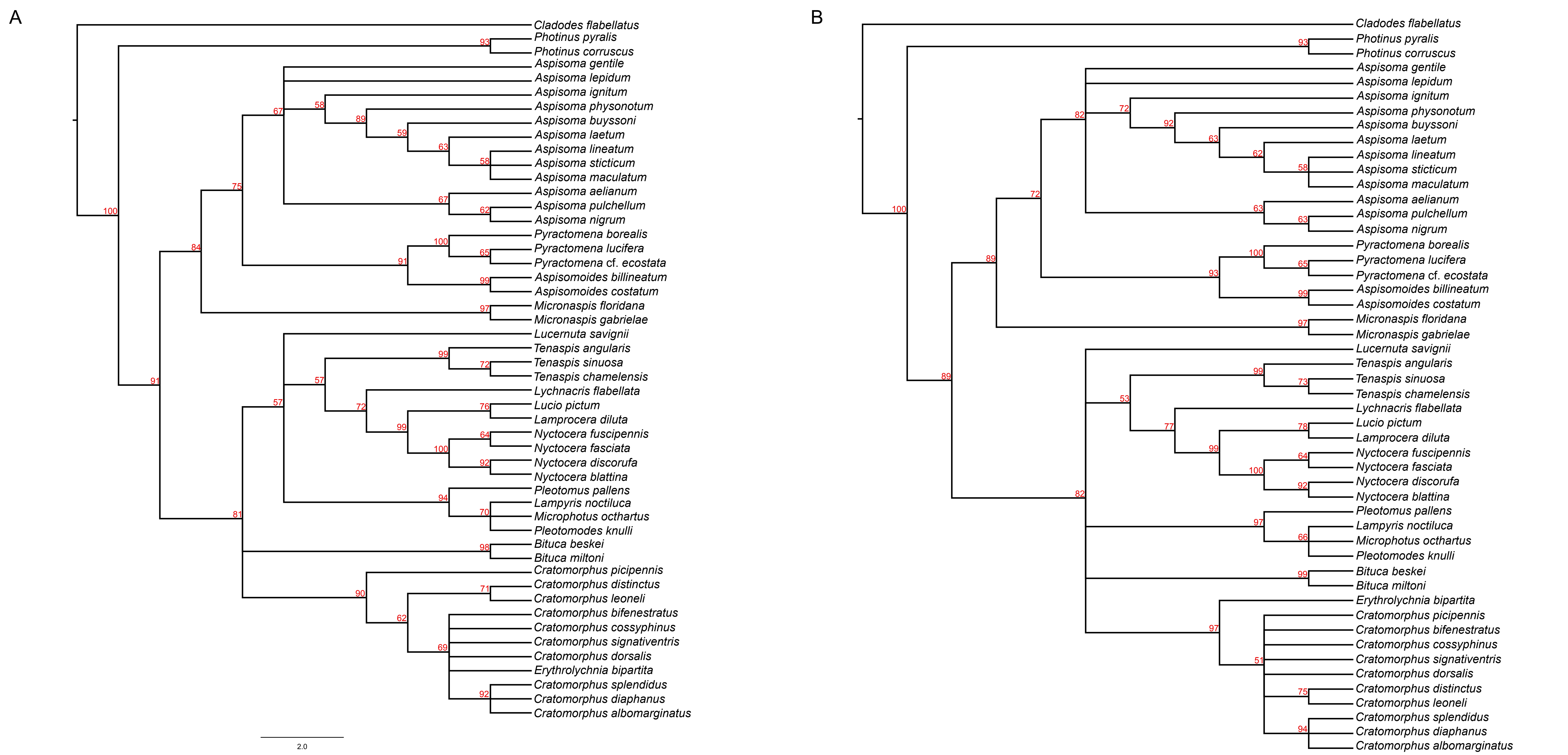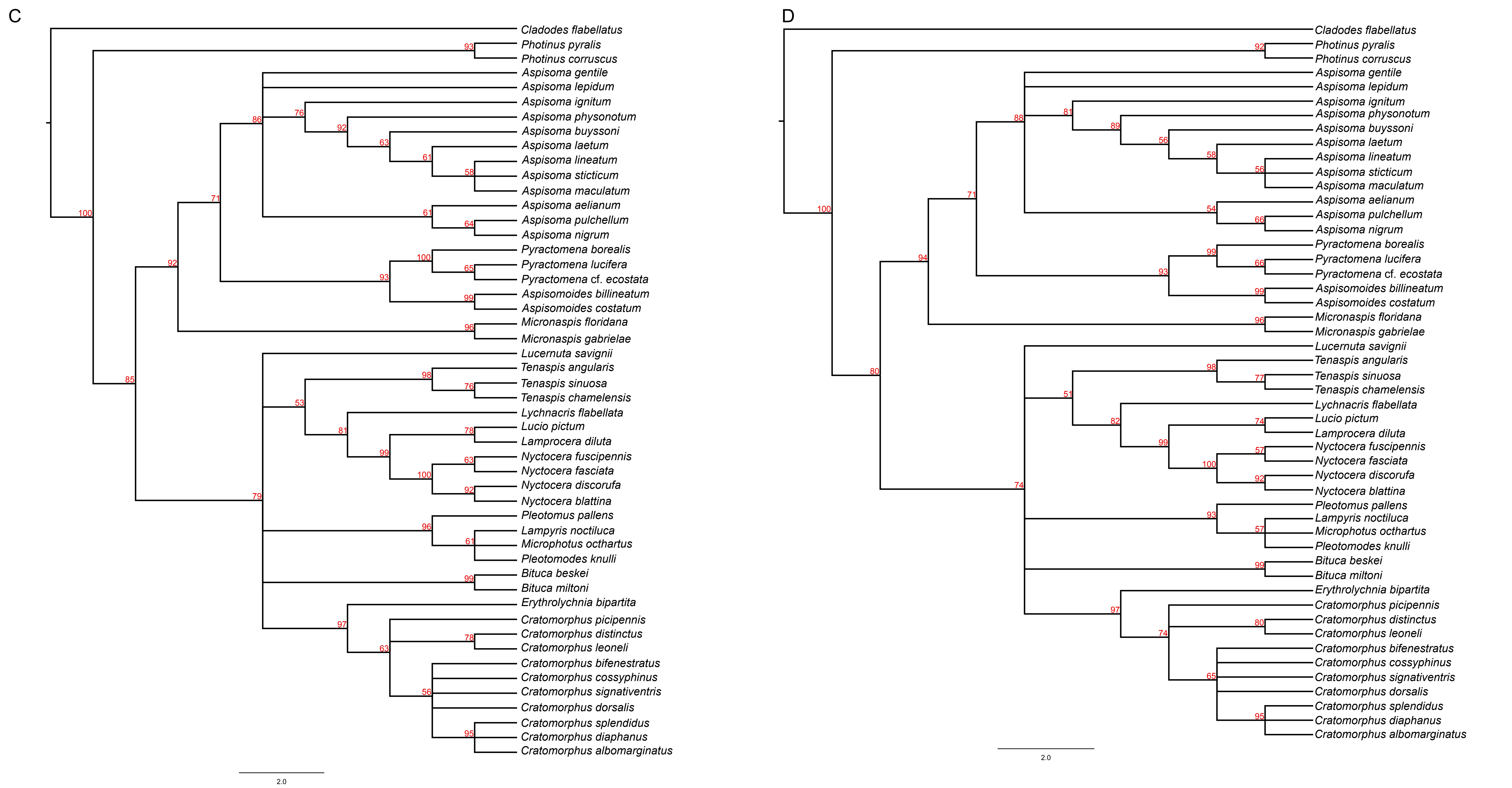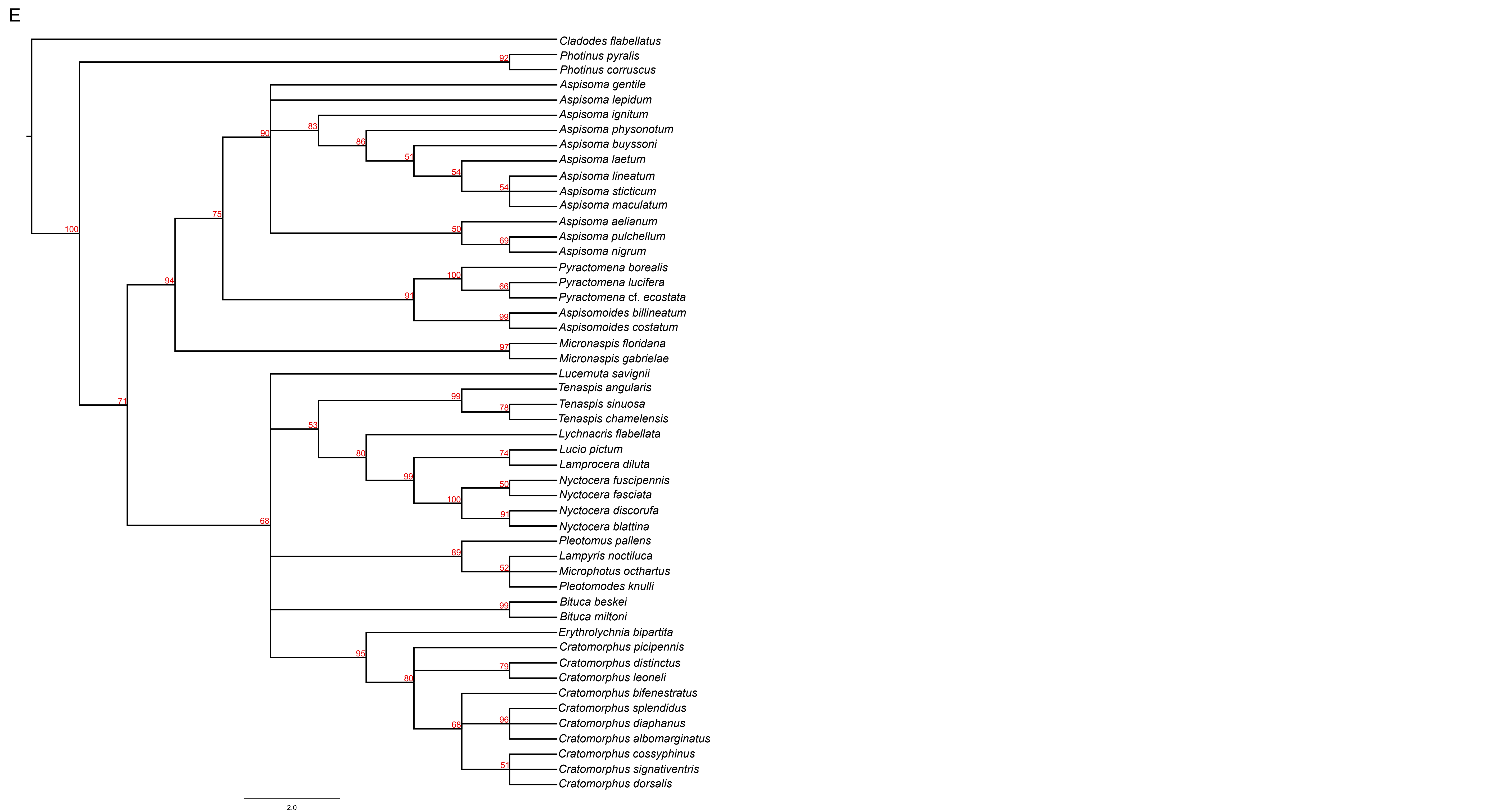

Supplement: S2 File — A: Maximum parsimony under implied weighting with K = 1; B: Maximum parsimony under implied weighting with K = 3; C: Maximum parsimony under implied weighting with K = 5; D: Maximum parsimony under implied weighting with K = 10; E: Maximum parsimony under implied weighting with K = 20. (PDF) [file pone.0354465.s002.pdf]
